# Genetic effects on molecular network states explain complex traits

**DOI:** 10.15252/msb.202211493

**Published:** 2023-07-24

**Authors:** Matthias Weith, Jan Großbach, Mathieu Clement‐Ziza, Ludovic Gillet, María Rodríguez‐López, Samuel Marguerat, Christopher T Workman, Paola Picotti, Jürg Bähler, Ruedi Aebersold, Andreas Beyer

**Affiliations:** ^1^ Excellence Cluster on Cellular Stress Responses in Aging Associated Diseases University of Cologne Cologne Germany; ^2^ Lesaffre Institute for Science and Technology, Lesaffre Marcq‐en‐Baroeul France; ^3^ Department of Biology Institute of Molecular Systems Biology, ETH Zürich Zürich Switzerland; ^4^ Institute of Healthy Ageing and Department of Genetics, Evolution & Environment University College London London UK; ^5^ Department of Biotechnology and Biomedicine Technical University of Denmark Lyngby Denmark

**Keywords:** complex traits, network effects, PKA signaling, QTL mapping, TOR signaling, Genetics, Gene Therapy & Genetic Disease, Proteomics, Signal Transduction

## Abstract

The complexity of many cellular and organismal traits results from the integration of genetic and environmental factors via molecular networks. Network structure and effect propagation are best understood at the level of functional modules, but so far, no concept has been established to include the global network state. Here, we show when and how genetic perturbations lead to molecular changes that are confined to small parts of a network versus when they lead to modulation of network states. Integrating multi‐omics profiling of genetically heterogeneous budding and fission yeast strains with an array of cellular traits identified a central state transition of the yeast molecular network that is related to PKA and TOR (PT) signaling. Genetic variants affecting this PT state globally shifted the molecular network along a single‐dimensional axis, thereby modulating processes including energy and amino acid metabolism, transcription, translation, cell cycle control, and cellular stress response. We propose that genetic effects can propagate through large parts of molecular networks because of the functional requirement to centrally coordinate the activity of fundamental cellular processes.

## Graphical abstract

Genetic variant effects can be described according to the spread of affected molecules across the cellular molecular network. Integrative multi‐omics analyses show how variants can shift network states in a global manner while other variant effects remain local or regional.

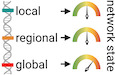

## Introduction

The genetic complexity of traits such as human body size and disease susceptibility has been well known for many years. Still there is a lack of understanding about how the ensemble of trait‐associated genetic variants and environmental factors are integrated at the molecular level. Recent work has proposed an “omnigenic” instead of a “polygenic” model for the genetic architecture of complex traits in humans, in principle stating that modulation of any gene that is expressed will have effects on all traits associated with a given tissue (Boyle *et al*, 2017). Central to the omnigenic model is the notion that genetic variants can affect “peripheral genes” and, via mediation by the cellular gene‐regulatory network, ultimately affect trait‐determining “core genes” (Liu *et al*, 2019). However, the mechanistic basis for the transmission of such effects across the network has remained unclear. In contrast, work focusing on the modular sub‐structure of molecular networks favors the view that specific sub‐networks or network modules are associated with disease phenotypes (Chen *et al*, 2008; Han, 2008; Schadt, 2009; Vidal *et al*, 2011; Peters *et al*, 2017). Fundamental to the modularity of a molecular network is the notion that cellular functions require particular stoichiometries between molecular components. This concept facilitates mechanistic understanding of the propagation of genetic variant effects since members of a network module are often subject to co‐regulation. In this latter framework however, stoichiometry and co‐regulation appear largely confined to the boundaries of individual network modules and consequently, variant effects would have limited reach. So far, no concept has been established that explains coordination across separate functional modules as the basis for genetic variant effects.

Here we propose a model that explains complex and far‐reaching genetic effects as alterations of cellular network states. We define a network state as a particular molecular configuration of a cell, that is, its specific composition of transcripts, proteins, other (small) molecules, and their molecular states as defined by, for example, protein post‐translational modifications. By “network” we refer to the entire ensemble of interactions occurring between these components within a cell. Our model is based on the assumption that only a small fraction of all theoretically possible network states are physiologically feasible and favorable. The cellular network is globally constrained by basic biophysical principles, such as the need to balance anabolic and catabolic activities to preserve homeostasis and by cellular limits such as maximum proteome size, protein cost, molecular crowding, or the availability of membrane space (Molenaar *et al*, 2009; Frei *et al*, 2020; Hu *et al*, 2020; Mori *et al*, 2021; Kleijn *et al*, 2022). Similar to requirements acting within functional modules, global constraints can result in coordinated changes across the entire network. Such network behavior has been demonstrated by studies on proteome allocation in *Escherichia coli* (You *et al*, 2013; Basan *et al*, 2015; Mori *et al*, 2021; Wu *et al*, 2022).

Mechanisms that shift network states within the boundaries of viability have evolved to provide efficient modulation of cellular function. While environmental alterations may be a driving force to establish network state regulation in microorganisms (Balakrishnan *et al*, 2021), cells in adult multicellular organisms reside in relatively stable environments. Still, these cells undergo elaborate developmental paths during which cellular states have to be adjusted. Importantly, basic biochemical pathways such as the carbohydrate metabolism and developmental processes are functionally coupled and subject to genetic variation (ERECTA; Keurentjes *et al*, 2008). The circadian clock represents another network state‐regulatory mechanism (Sun *et al*, 2021), which is also modulated through genetic variation (Kerwin *et al*, 2011; Jones *et al*, 2019).

Network states can change through sensing the cell's environment or internal molecular state. Control mechanisms exemplified by signaling pathways ensure that the network remains in a viable state while adjusting a multiplicity of cellular functions to a particular environment. Genetic variants can shift network states within the boundaries of viability and can act on network state‐modifying mechanisms in different ways: by affecting sensing or signaling, but also by modifying the state of (intermediate) metabolites being sensed. Using this concept as the basis for our model, we propose a new classification of genetic effects: (i) variants affecting only a very small part of a network, such as a single protein or protein complex (subsequently called “local effects”), (ii) variants affecting single or grouped modules of the network, such as individual signaling, regulatory or metabolic pathways (subsequently called “regional effects”), and (iii) variants affecting the balance between network modules that reside in regions of the network which are less connected to each other by (macro)molecular interactions. The latter variants change particular aspects of the molecular configuration of a cell, which require a far‐reaching adjustment of the cellular metabolism and corresponding module activities (subsequently called “global effects”). Here, we present examples of all three types of genetic variants.

We set out by applying this paradigm to a complex cellular phenotype, namely the ability of yeast to efficiently overcome temporary temperature stress. Heat stress is among the best‐studied perturbations in yeast (Gasch *et al*, 2000). Many aspects, such as organization of the proteome into liquid phases (Wallace *et al*, 2015), are conserved in human cells (Franzmann & Alberti, 2019). Hence, it remains of great interest to understand how genetic variation influences thermotolerance and cellular stress resistance in general. Using a collection of yeast segregants, we studied the genetic contribution to efficient outgrowth after temporary heat stress, which we refer to as “heat resilience.” Transcriptomic, proteomic, and phosphoproteomic measurements were employed to comprehensively chart the molecular network state in each segregant strain (Grossbach *et al*, 2022). We found genetic variants with effects on specific, heat‐stress‐related proteins and others that determine resilience through a broad cellular program that is closely related to PKA and TOR signaling. The network state that results from these signaling activities (PKA/TOR‐related or PT network state) can be summarized as a single quantitative trait. We characterize the global quantitative alterations of transcripts, proteins, phosphorylation, and metabolic features that constitute changes of this network state across a wide range of environmental and genetic influences. Finally, we show that the PT network state is conserved in fission yeast, which evolved aerobic alcohol fermentation in parallel to budding yeast over an estimated evolutionary distance of more than 200 million years (Rhind *et al*, 2011).

## Results

### Genetic and molecular mapping of heat resilience

Previous studies of thermotolerance in yeast found genetic determinants that convey advantages for growth under persisting high temperature (Steinmetz *et al*, 2002; Sinha *et al*, 2006, 2008; Yang *et al*, 2013; Caspeta *et al*, 2016; Weiss *et al*, 2018; Abrams *et al*, 2022) and for heat shock survival (Gibney *et al*, 2013; Jarolim *et al*, 2013). Here, we aimed to understand which factors determine the time required to re‐establish maximum growth rates following a short, sub‐lethal heat stress episode (heat‐induced lag).

**Figure 1:**
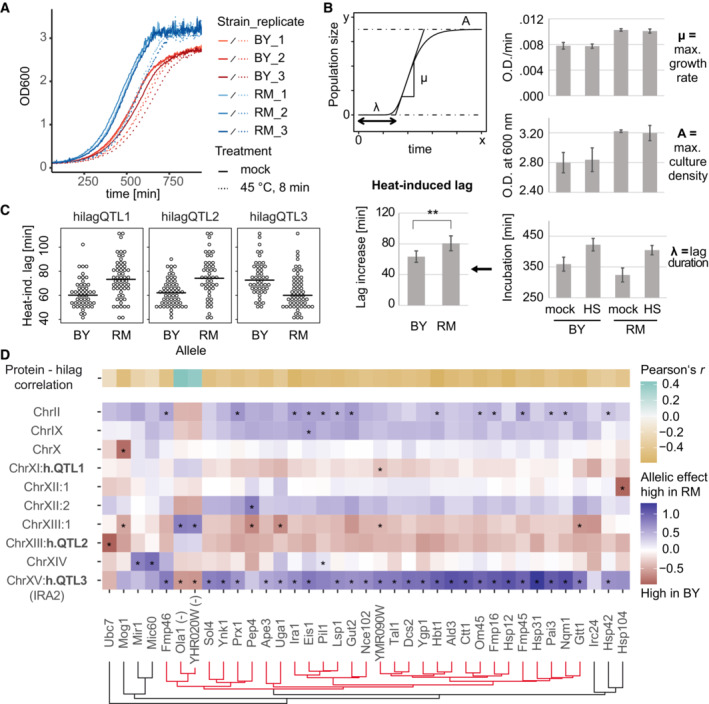
Identification of genetic and molecular determinants of heat‐induced lag Growth curves recorded for BY4716 and RM11‐1a after transient heat stress treatment (45°C, 8 min) and dilution into fresh growth medium.Quantification of growth parameters. Heat‐induced lag is calculated as the difference in lag duration (λ) between heat‐treated (HS, 45°C, 8 min) and mock‐treated samples for each strain (error bars indicate mean ± SD; *n* = 3 biological replicates, Student's *t*‐test; **P < 0.01).Distribution of hilag measurements for strains carrying opposing parental alleles at significant loci identified by QTL mapping (15% FDR, see Fig EV1C).Mapping of heat‐induced lag to protein abundances and overview of corresponding pQTL. 37 proteins passing a 20% FDR threshold as predictors of heat‐induced lag are shown. The top row shows correlation of the protein's abundance with heat‐induced lag. Fields in all other rows show allelic effects (difference in mean protein abundance between strains carrying the RM allele compared to the BY allele at the indicated loci) and asterisks indicate significant pQTL‐target relations as described in (Grossbach *et al*, 2022). Columns or proteins were ordered according to hierarchical clustering. Abundance scaling of Ola1p and YHR020W (marked by “(−)”) was inverted across the segregants for clustering. h.QTL, hilagQTL. Source data are available online for this figure.

**Figure EV1:**
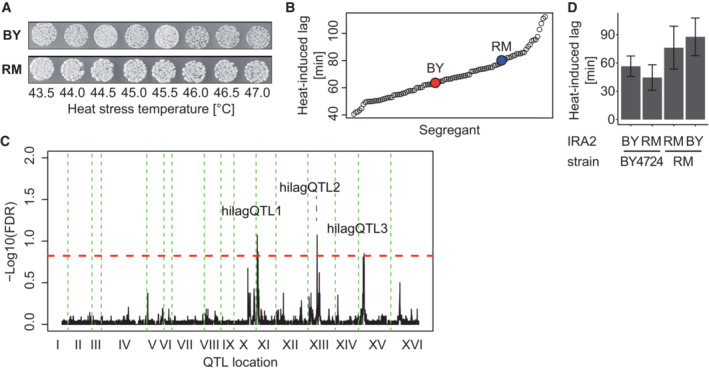
Supporting experiments and QTL mapping result for heat‐induced lag Viability test for heat stress treatment. Samples from exponentially growing cultures of BY or RM were subjected to transient heat stress treatment (ramping from 25°C to indicated temperature at 1 K/s, followed by 8 min exposure at constant peak temperature) and spotted onto YPD plates following appropriate dilution. Spots were photographed after 1.5 days at 30°C.Distribution of heat‐induced lag measurements across 100 segregants and the parental strains as indicated.QTL mapping result for heat‐induced lag. Dashed red line represents 15% FDR threshold based on comparison between mapping of true against permuted trait values. Loci that passed the threshold are indicated.Heat‐induced lag measurements (*n* = 4 biological replicates) in BY4724, which is closely related to the parental strain BY4716, and in RM11‐1a as well as in derivatives after allele‐swapping of *IRA2* (Smith & Kruglyak, 2008). Error bars indicate mean ± SD.

To find genetic determinants of the thermotolerance trait, we made use of a well‐studied cross between isogenic haploid derivatives of the common lab strain S288c, BY4716, and the vineyard isolate strain RM11‐1a (BYxRM collection; Brem *et al*, 2002). We exposed exponentially growing cultures of the parental strains and of 100 segregants to transient heat stress at 45°C for 8 min, which we confirmed to be sub‐lethal (Fig EV1A). Following heat stress or mock treatment, samples of the cultures were diluted into fresh medium and growth curves were recorded (Fig 1A). From these curves, strain‐specific growth characteristics were inferred and used to estimate the heat‐induced lag (Fig 1B).

Both parental strains scored close to the median (66 min) of all measurements of heat‐induced lag across the collection (BY: 64 min, RM: 81 min, Fig EV1B), consistent with a transgressive pattern of segregation and presumably, a polygenic basis. To localize genetic factors that contribute to the variation of heat‐induced lag, we performed mapping of quantitative trait loci (hilagQTL) using a Random Forest‐based approach (Michaelson *et al*, 2010). QTL are genomic loci (regions) whose genetic variation across a population (here: the BYxRM cross) correlates with a quantitative trait (here: heat‐induced lag). Three loci passed a threshold of 15% FDR (Fig EV1C) and showed variable effect directions with comparable effect size (Fig 1C), partially accounting for transgressive segregation. An additive random‐effects model based on the most significant markers at these loci explained up to 35% (adjusted *R* ^2^) of trait variability across the strains. Based on the parental replicate measurements, we estimated the heritability of heat‐induced lag to be 46% (adj. *R* ^2^), leaving about a quarter of the estimated heritability unexplained by effects at these QTL. Regarding potential mediators of the QTL effects, we noted that the locus on Chromosome XV (hilagQTL3) contained *IRA2*, which is known to induce widespread changes of transcriptional and cellular traits in this yeast cross (Smith & Kruglyak, 2008; Nguyen Ba *et al*, 2022). Replacing *IRA2* by the RM allele in the BY background indeed approximated the effect of the QTL (Fig EV1D).

To identify molecular mechanisms mediating the effects of genetic variation on the heat stress trait, we compared protein abundances recorded for each strain (Grossbach *et al*, 2022) to trait differences across the collection. Specifically, we trained a Random Forest model predicting heat‐induced lag as a function of protein abundance. Setting a permutation‐based false‐discovery rate (FDR) threshold of 20%, we identified 37 predictive proteins (“hilag predictor proteins”; Fig 1D). This protein set contained candidates that are well‐known contributors to heat shock survival, such as Hsp104 (Cherkasov *et al*, 2013) and Ctt1 (Davidson *et al*, 1996). We further asked which genetic loci would be associated with variation in these 37 hilag predictor proteins. We mapped protein abundance QTL (pQTL) for this set of proteins, which revealed loci with strong effects on few, individual proteins. For example, a pQTL on Chromosome XII affected levels of Hsp104, presumably in *cis*, since it was found close to the *HSP104* locus. Mog1, a nucleotide‐release factor for the Ran GTPase Gsp1 that participates in the osmotic stress response (Lu *et al*, 2004), was the sole significant target of a pQTL on Chromosome X.

On the other hand, a subset of 30 out of the 37 hilag predictor proteins exhibited highly correlated changes across the strain collection (red cluster in Fig 1D), 28 of which shared a significant pQTL at the *IRA2* locus (hilagQTL3). We further noticed that genetic loci often had opposing allelic effects across many of these proteins. Notably, the strains with the RM allele at hilagQTL3 had elevated levels of these proteins, whereas the RM alleles at the loci hilagQTL1 and hilagQTL2 reduced levels of the same proteins. These effects were consistent with the effect directions of the three hilagQTL on heat‐induced lag (Fig 1C).

Taken together, our mapping results suggested that differences in the extent of heat‐induced lag and its transgressive segregation in this cross can be explained both by variants that change the abundance of individual proteins such as Hsp104, as well as by variants that affect a broader spectrum of proteins. In particular, mapping the trait to protein abundances indicated that the *IRA2* locus impacted a set of proteins with coordinated expression, which was additionally modulated by hilagQTL1, hilagQTL2, and potentially other loci.

### Quantification of a PKA/TOR‐related program of gene expression

**Figure EV2:**
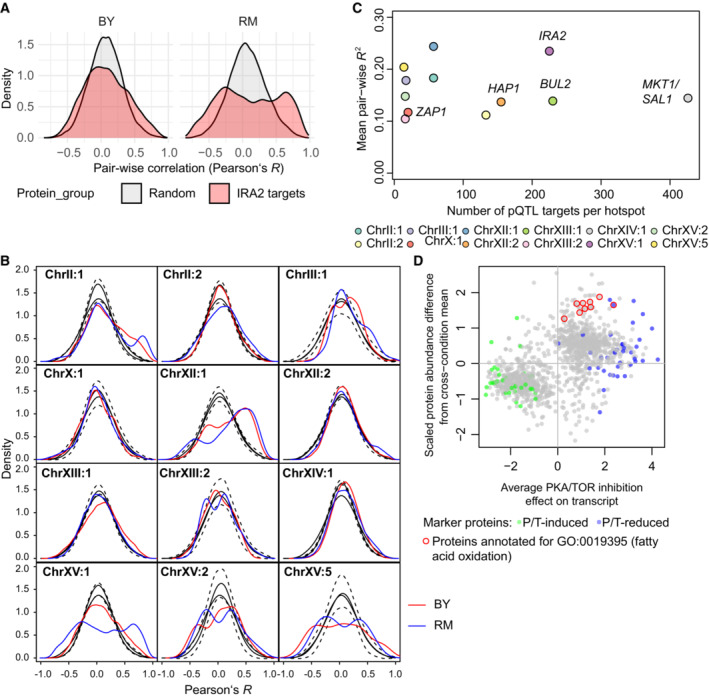
Coordinated expression among targets of pQTL hotspots and illustration of proteomic state of samples grown on oleate as carbon source Test for coordinated expression among protein abundance targets of the *IRA2* pQTL hotspot. Pair‐wise correlation among 225 proteins shown separately for sets of strains carrying either the BY or the RM allele at the marker corresponding to the *IRA2* gene. The gray‐colored area represents a representative example of the distribution of pair‐wise correlations in abundance‐matched but otherwise random sets of proteins. The average distribution as well as standard deviation of the frequency distribution (200 bins) are shown in (B).Same test for coordination as in (A) but for each of 12 pQTL hotspots as described in (Grossbach *et al*, 2022). Pair‐wise correlations between protein abundance targets of the indicated hotspot were calculated in sets of strains split by the corresponding most significant marker (BY: red curve, RM: blue curve). Black solid lines show the average distribution of pair‐wise correlations in random sets of proteins that were matched in abundance to the targets of the respective hotspot. These pair‐wise correlations were calculated in the same sets of strains as used for the actual targets, represented by two different solid lines. Dashed lines show the maximum and minimum among 1,000 random samples of proteins, calculated for 200 bins across the range from −1 to +1.Comparison of coordinated expression of pQTL hotspot targets (average of all pair‐wise correlations as shown in (B)) and the number of targets for each hotspot.Comparison between protein abundances differences (scaled and centered across 10 carbon sources) for the sample grown on oleate from (Paulo *et al*, 2016) to the average effect of PKA and TOR inhibition (20 min, Kunkel *et al*, 2019) on the corresponding transcript. PKA (P) and TOR (T)‐induced and TOR (T)‐reduced marker proteins are highlighted in green and blue, respectively.

The *IRA2* locus (coinciding with hilagQTL3) is a pQTL hotspot, that is, a genomic region affecting the levels of significantly more proteins than expected by chance (Grossbach *et al*, 2022). Our analysis above suggested that target proteins of this locus are subject to a common regulatory program. In order to corroborate this notion, we tested for coordinated expression of all 225 *IRA2* target proteins (pQTL targets at 10% FDR) within groups of strains defined by their allele at the *IRA2* locus. Thus, we removed effects of the *IRA2* allele and partially removed effects of neighboring variants in linkage disequilibrium (LD). We speculated that, if *IRA2* target proteins were commonly affected by additional loci, there should be a remaining correlation that is greater than random. This was indeed the case, as abundance‐matched but otherwise random sets of proteins exhibited lower average pair‐wise correlations (mean *R* ^2^ = 0.06) than targets of the *IRA2* locus (mean *R* ^2^ = 0.16, empirical *P*‐value < 1E−3, Fig EV2A) after removing effects of the *IRA2* locus itself. We applied the same test for coordination among their targets to 11 other hotspots of protein abundance regulation (Fig EV2B). The degree of coordination among targets of the *IRA2* hotspot was larger than for 4 other hotspots with more than 100 targets (Fig EV2C). In sum, hotspots often affected network modules that remained coordinated across multiple genetic perturbations. However, the *IRA2*‐related program encompassed an exceptionally large set of strongly coordinated proteins.

Ira2 is a GTPase‐activating protein (GAP) that negatively regulates Ras1/2, which are upstream regulators of the PKA signaling pathway. The RM allele of *IRA2* encodes a protein with higher activity compared to the BY allele (Smith & Kruglyak, 2008; Nguyen Ba *et al*, 2022). Hence, the effects of the *IRA2* locus on protein abundances likely result from genetic influence on PKA activity. The remaining coordination among *IRA2* targets in strains with identical alleles at this locus suggested that more loci could affect the same set of proteins. For example, a region on Chromosome XIII, which includes the *BUL2* gene, had strong and opposite effects across many *IRA2* targets (“ChrXIII:1,” Fig 1D). Bul2 is involved in the endocytosis of amino acid permeases (Abe & Iida, 2003; Merhi & Andre, 2012) and thereby likely affects TORC1 signaling (here referred to as TOR signaling for simplicity; Kwan *et al*, 2011). Strong crosstalk between the TOR and PKA pathways has been shown repeatedly (Chen & Powers, 2006; Soulard *et al*, 2010; Ramachandran & Herman, 2011; Zhang *et al*, 2011). They also converge on common downstream effectors such as Rim15 (Reinders *et al*, 1998; Pedruzzi *et al*, 2003; Swinnen *et al*, 2006; Lee *et al*, 2013) and a range of transcription factors (Reinders *et al*, 1998; Pedruzzi *et al*, 2003; Swinnen *et al*, 2006; Lippman & Broach, 2009; Lee *et al*, 2013; Kunkel *et al*, 2019).These observations led us to hypothesize that a large part of the protein abundance changes explaining the extent of heat‐induced lag was under the common control of the PKA and TOR pathways.

**Figure 2:**
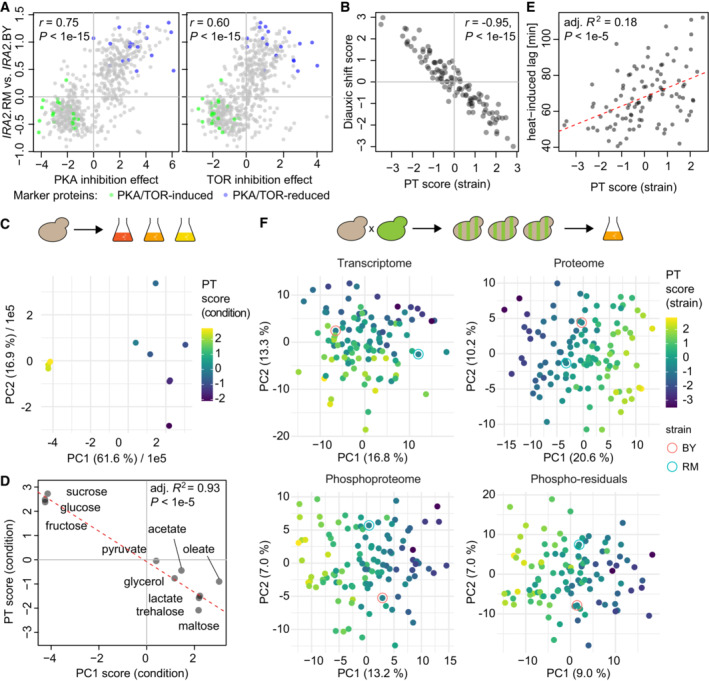
Scoring of a PKA‐ and TOR‐signaling‐dependent network state Comparison of the effects of chemical inhibition of PKA (left) and TOR (right, both after treatment for 20 min, data from Kunkel *et al*, 2019) on transcripts to difference according to parental *IRA2* allele on the corresponding protein abundances in the BYxRM collection. Assignment of transcripts to PT‐induced and PT‐reduced sets is indicated by green and blue dots, respectively. The PT score is calculated as the difference between the medians of marker protein abundance (scaled and centered across the BYxRM collection) in the two sets.Comparison of a score based on transcript level changes during the diauxic shift (Brauer *et al*, 2005) to the PT score for all strains of the BYxRM collection.PCA of proteomic data for BY4742 grown on different carbon sources (Paulo *et al*, 2016). Coloring by PT score for each sample, calculated based on protein abundances after scaling and centering across conditions, yielding a comparison to the global mean for the studied range of carbon sources. Fractions of total variance explained by each PC are indicated.Comparison of sample scores on PC1 (panel C) to the PT score. Dashed red line shows the linear regression model.Correlation of PT score with heat‐induced lag measurements per strain of the BYxRM collection. Dashed red line shows the linear regression model.PCA for molecular variability between strains of the BYxRM collection based on transcript, protein, and phosphopeptide abundances as well as phospho‐residual levels (see Materials and Methods). Coloring by PT score. The PT score explained up to 86, 80, and 68% variability (adjusted *R* ^2^) between segregant scores along PC1 of PCAs based on protein, phosphopeptide, and phosphopeptide‐residual abundances, respectively. For the transcriptome, the PT score correlated more strongly with the second PC (adj. *R* ^2^ = 0.44). Source data are available online for this figure.

In order to corroborate the relevance of PKA and TOR signaling in this context, we compared our collection‐wide protein abundance data to transcript abundance changes following chemical inhibition of the PKA and TOR pathways (Kunkel *et al*, 2019). Indeed, we observed that the effect of the RM allele of *IRA2* in our dataset was highly correlated to the effect of chemical PKA and TOR inhibition (Pearson's *r* = 0.75 and 0.60, respectively, both significant at *P* < 1E−15, Fig 2A). We next sought to quantify the status of PKA/TOR (PT) signaling in a given population of yeast cells. We therefore conceived a score that summarizes overlapping outcomes of PKA and TOR activity (“PT score”) based on a set of 47 PT‐induced and 44 PT‐repressed marker genes selected by thresholding and filtering the chemical inhibition data (see Materials and Methods). We then made use of the overlap between these markers and our proteomic data for the BYxRM cross to select 18 and 22 abundance markers for induced and repressed PKA/TOR activity, respectively. The PT score is the difference between the median abundance of proteins in the PT‐induced set and the median abundance of those in the PT‐repressed set in a specific yeast strain (Fig 2A). This score quantifies relative differences in PT activity between strains.

To further establish the PT score as a measure of combined activities of the PKA and TOR pathways, we compared it to a similar score based on the changes in molecular configuration of the cell that occur at the shift from glucose to ethanol consumption (diauxic shift). Further, we applied the PT score to yeast samples grown on a range of carbon sources. Both scenarios involve concerted changes in the PKA and TOR pathways (Pedruzzi *et al*, 2003; Broach, 2012). First, we extracted a set of markers based on transcript abundance changes during the diauxic shift (31 diauxic shift‐induced and 30 shift‐reduced proteins; Brauer *et al*, 2005). The difference in medians between these marker sets showed almost perfect correlation with the PT score across BYxRM segregants (Fig 2B, *r* = −0.95, *P* < 1E−15). Next, we analyzed a proteomic dataset generated for a strain derived from the common lab strain S288c (BY4742) grown on 10 different carbon sources (Paulo *et al*, 2016). We calculated PT scores for each condition based on the abundances of 40 and 39 proteins that overlapped with the PT‐induced and PT‐reduced marker sets, respectively. As expected, the highest scores were attributed to populations grown on glucose, sucrose, and fructose (Paulo *et al*, 2016), while other carbon sources led to lower PT scores. To analyze the extent to which proteomic changes under these different carbon sources correlated with PT activity, we performed principal component analysis (PCA), which reduced the complexity of the proteomic changes to a low‐dimensional space (Fig 2C). We then compared PT scores calculated for the individual growth conditions to their respective placement along the first principal component (PC1). We observed a strong correlation between the positioning of the growth conditions along PC1 and their PT scores. The predictive accuracy of the PT score for PC1 scores in a linear model across conditions reached up to 93% (adj. *R* ^2^, *P* < 1E−5, Fig 2D). Since PC1 explained more than 60% of the proteome variation in the data, changes of PT activity under these conditions are associated with a major reorganization of the proteome. A noticeable exception was the sample grown on oleate, which deviated most from the observed correlation between PC1 and the PT score. Comparison of the proteomic state in this sample to the results of chemical inhibition of PKA and TOR still showed good agreement (linear model adj. *R* ^2^ = 0.28, *P* < 1E−25, Fig EV2D). However, proteins annotated for fatty acid oxidation (GO:0019395) were more strongly increased than expected. Together with the deviation of this sample from the global trend, this suggests that growth on lipids could result in an alternative cellular configuration, which is not entirely captured in the PT score. Taken together, these comparisons indicate that the PT score captures a mode of cellular re‐configuration that is related to the diauxic shift and to the changes occurring between a broad range of carbon sources, which agrees with the known roles of PKA and TOR signaling.

Consistent with the direction of effect of the *IRA2* allele (hilagQTL3, the less active allele in BY increases PKA activity, Fig 1C), the PT score was positively correlated with heat‐induced lag and explained up to 18% variability of this trait (linear model adjusted *R* ^2^, *P* < 1E−5, Fig 2E), exceeding the effect of any single hilagQTL, including the *IRA2* locus itself. Further, the PT score and heat‐induced lag were still correlated even after correcting for all three hilagQTL (partial correlation *R*' = 0.19, *P* = 0.06) indicating that additional variability in the molecular network that determines heat‐induced lag was captured by the PT score. As noted above, we observed inverse effects of the other hilagQTL on *IRA2* targets. Consistently, hilagQTL1 and hilagQTL2 had significant and inverse effects on the PT score (mean difference between RM and BY allele‐carrying strains: +0.59 and +0.67, respectively, both *P* < 0.05) as compared to hilagQTL3 (−1.19, *P* < 1E−5).

We next asked to what extent the PT score was predictive for proteome differences caused by genetic variation. We found that the PT score correlated with a major fraction of overall proteome variation in the BYxRM collection. For example, the PT score strongly correlated with the first dimension in a PCA of the BYxRM proteomes (Fig 2F). Overall, linear models using the PT score reached proteome‐wide significance (FDR <0.05) for 622 of 1,862 (33%) protein abundances, explaining on average up to 22% of their total variation (mean adj. *R* ^2^ in a linear model). Beyond protein abundance, the PT score was also predictive for substantial variation in transcriptome and phospho‐proteome data from the same strain collection (Fig 2F). Hence, the PT score captured major parts of molecular differences across the BYxRM collection in a single quantitative value. Conversely, the configuration of the cellular molecular network was shifted along an axis defined by this scalar, which we refer to as the “PT network state” in the following.

### Effect of PT network state differences on functional modules

**Figure 3:**
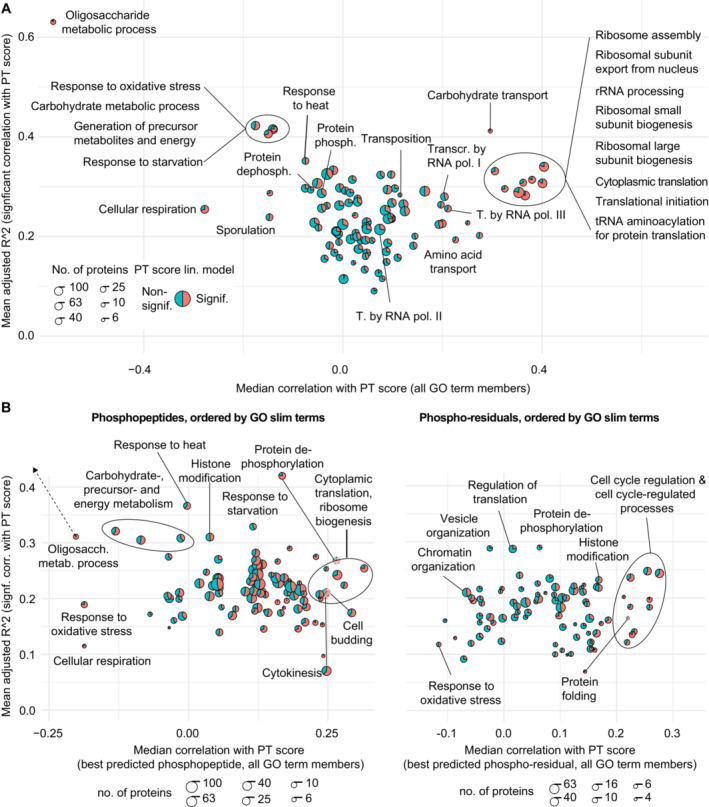
Correlation between PT score and functional modules (GO slim terms) Illustration of linear model properties using the PT score as predictor for protein abundances, separated by GO slim terms (Saccharomyces Genome Database). The size of each circle represents the number of proteins per GO term (logarithmic scaling) as indicated. Red and blue segments correspond to the proportion of proteins for which the PT score is or is not a significant linear predictor (FDR < 0.05), respectively. Diagram created using R package “scatterpie.”Same representation of slim GO terms as in A but based on best predicted phosphopeptide (left panel) or phosphopeptide‐residual (right panel) for each GO‐annotated protein (see text for details). The GO term “oligosaccharide metabolic process” was moved inside the diagram boundaries for visibility. Some terms were moved outside of grouped term boundaries for clarity, as indicated.

The analyses above were consistent with the coordination of a large diversity of cellular processes by the PKA/TOR‐related regulatory program. In order to characterize the association between the PT score and cellular processes and functions, we first assigned proteins in our dataset for the BYxRM collection and corresponding phosphopeptides to a curated set of Gene Ontology (GO) slim terms (Cherry *et al*, 2012). We then correlated the individual proteins and peptides in each of these GO terms with the PT score. Each GO term is represented by an individual pie chart that shows the proportion of significantly predicted proteins and is positioned according to the average direction and accuracy of prediction by the PT score across the members of the term (Fig 3). When applied to protein abundance data, the PT score was a significant linear predictor (FDR <0.05) for more than half of the assigned proteins in close to a third of all tested GO terms (29 of 95 terms with at least 3 members; Fig 3A). Strong and highly directional correlations with the PT score comprised well‐known targets of PKA and TOR. Hence, our analysis confirms that a great diversity of cellular processes is affected by alterations in PT signaling (Conrad *et al*, 2014).

This conclusion was further corroborated by correlating the PT score with protein phosphorylation data obtained for the same strain collection (Fig 3B). Intensity changes of phosphopeptides in proteomics data may result from two phenomena: either the abundance of the “host protein” containing the phosphopeptide may change or the phosphorylation rate of the respective residue may change. In order to distinguish those two cases, we have used two different measures to quantify protein phosphorylation: first the “raw” abundance measurement of a phosphopeptide and second protein abundance‐corrected residuals (“phospho‐residuals,” Grossbach *et al*, 2022). Although the former reflects a combination of protein abundance changes and changes in protein phosphorylation, the latter measure is corrected for changes in protein abundance and hence solely quantifies changes in phosphorylation. When several phosphopeptides were measured per protein, we considered the peptide that was best predicted by the PT score (highest adj. *R* ^2^) to reflect the functional state of the corresponding protein (see Material and Methods for a discussion of potential bias).

Appendix Text 1 contains an extensive discussion of the observed correlations of cellular processes with PT network differences elicited by natural genetic variation. Dataset EV1 contains data for individual transcripts, proteins, and phosphopeptides in our dataset. These alterations span large distances in the molecular network and affect a diversity of cellular processes, including but not limited to well‐known targets of PKA and TOR signaling such as cytoplasmic translation, ribosome biogenesis, oxidative phosphorylation, and stress responses as illustrated in Fig 3.

### Genetic variants affecting the PT network state have global network effects

**Figure 4:**
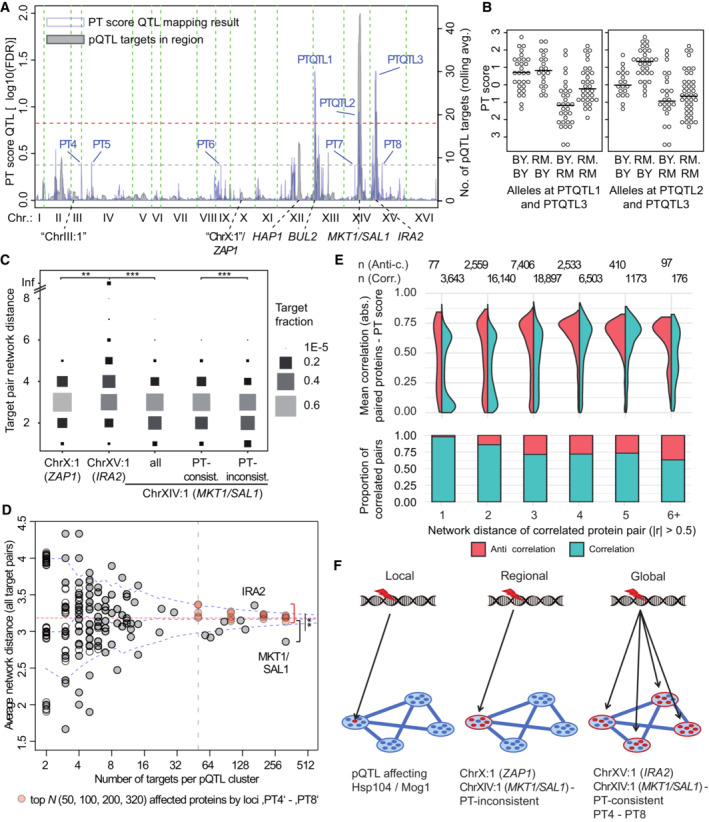
Genetic effects on the PT score and network state changes QTL mapping of the PT score (blue curve) and overlay of the number of pQTL targets by marker (gray curve; rolling average of pQTL number across windows comprising 20 genetic markers). Dashed red line represents 15% FDR threshold for QTL mapping and dashed gray line represents the 42% FDR threshold applied for selection of PT4–PT8. Other PTQTL falling below this threshold were excluded by setting a threshold on the number of pQTL (0.67 percentile of the rolling average of pQTL, see panel A). Known variants are indicated.Distribution of PT scores for strains carrying indicated combinations of parental alleles at significant QTL for the PT score.Distribution of network distances (shortest path; STRING database physical interaction‐based graph) for all pair‐wise combinations of targets (or target subgroups as indicated) of the indicated pQTL hotspots. Some targets of pQTL hotspot ChrXV:1 (close to *IRA2*) could not be connected on this graph (indicated by a line at the top of the plot) and were assigned the highest distance of other pairs (8). A two‐sided Poisson test was applied to evaluate differences between the distributions of network distances (confidence levels: **P < 0.01, ***P < 0.001).Comparison of network distances for all pair‐wise combinations of targets of 265 clustered pQTL (see Materials and Methods) to the number of targets. In some cases, clusters had identical numbers of targets as well as mean network distance. For these clusters, “empty” circles with slight variation on the y‐axis were added for illustration. Dashed blue lines show the distribution of average pair‐wise network distances for random samples of various sizes (0.05, 0.5, and 0.95 percentile). The dashed red line represents the mean distance of all protein pairs in the network (3.188). Red data points represent network distances among variable numbers of most affected proteins (top *N* = 50, 100, 200, or 320) of the loci PT4 to PT8. *T*‐tests were applied to test for a significant difference at each “top *N*” against averages of network distance among targets of 7 clusters with more than 50 targets (right of the dashed vertical line, but excluding IRA2; **P < 0.01).(Upper panel) Comparison of individual correlations of proteins in correlated protein pairs with the PT score across the BYxRM collection and their distribution across network distance bins (distances greater than 6 were collapsed into the 6^th^ bin). Protein pairs were selected for correlation (|*r*| > 0.5) and split according to the sign of the correlation as indicated. A high (absolute and sign‐corrected) mean correlation of the individual proteins in a pair with the PT score indicates association of the protein pair with the PT network state. (Lower panel) Proportion of correlated versus anti‐correlated protein pairs (|*r*| > 0.5) across individual network bins.Proposed classification of genetic variant effects according to the network position of affected molecules.

The analysis above revealed strong influence of PT network state alterations on the molecular configuration of strains in the BYxRM collection. Consequently, we sought to identify genetic variants that affected the PT network state. No segregant showed a PT score that fell between those of the parental strains (BY: −0.68, RM: −0.69), indicating highly transgressive segregation of this trait. Remarkably, the composition of the proteome and phosphoproteome of the parental strains also showed little difference after dimension reduction (Fig 2F). We performed QTL mapping as above (Fig EV1C) using the PT score as a target trait and detected three regions containing predictive markers (PTQTL at 15% FDR, Fig 4A). The RM allele at PTQTL3 on Chromosome XV, which was close to *IRA2* (< 10 kb), was associated with a lower mean PT score (−1.41, Student's *t*‐test *P* < 1E−8). This is consistent with the known role of Ira2 in Ras/PKA signaling and the stronger PKA‐inhibitory effect of the RM variant (Smith & Kruglyak, 2008), suggesting that *IRA2* mediated the effect at this locus. The allelic effect of PTQTL3 was counteracted by the effects of PTQTL1 and PTQTL2 (PTQTL1: +0.58, PTQTL2: +0.63, *P* < 0.05). PTQTL1 on Chromosome XII coincided with *BUL2* (Kwan *et al*, 2011). The PTQTL2 region contains known variants in *MKT1* and *SAL1*, which contribute to mitochondrial genome instability and consequently, a higher proportion of petite cells in populations of BY compared to RM (Dimitrov *et al*, 2009). There was considerable epistatic interaction between these PTQTL, which underlines the complexity of the PT network state (Fig 4B). Specifically, the effect size of PTQTL1 (close to *BUL2*) was stronger in strains carrying the RM allele at PTQTL3 (close to *IRA2*, +1.10) than in strains carrying the BY allele (+0.21, *P* < 0.05 for the interaction term), whereas the effect size of PTQTL2 (close to *MKT1*/*SAL1*) was stronger in strains carrying the BY allele (+1.14) than in those carrying the RM allele (+0.24, *P* < 0.05 for the interaction term) at PTQTL3. Potentially related interactions between major pleiotropic effect loci in determining cellular fitness traits have been described recently (Nguyen Ba *et al*, 2022).

When combining these three PTQTL together with the PT score in a linear model, we still observed significant partial correlation between the PT score and the abundance of many proteins. Specifically, 511 proteins showed significant partial correlation with the PT score in the combined linear model as compared to 622 proteins that correlated with the PT score alone (FDR <0.05). Taken together with the previous analysis of cellular processes (Fig 3), this suggests that other genetic variants altering the PT network state also cause a widespread reorganization of cellular physiology: in order to maintain cellular homeostasis yeast cells seem to adapt multiple processes that are under the common control of PKA and TOR signaling. Thus, a variant changing one process may cause coordinated change of another process that is distant in the molecular network, if that process needs to be adapted to maintain cellular homeostasis. Based on this notion, we are proposing a classification of genetic variants into “local,” “regional” and “global” effects. Genetic effects are defined as “local” if they pertain to single proteins (or complexes) and as “regional” if the effect spreads across individual or strongly related functional “modules.” Genetic effects are called “global” if they lead to coordinated change of distant functional modules to meet, for example, basic molecular or evolutionary constraints. The *IRA2* locus in the BYxRM cross serves as a paradigmatic example of global effects. Importantly, we do not claim that such global network effects are limited to alterations of the PT signaling state. Instead, alterations of other central signaling pathways may also exert effects on a broad range of coordinated cellular functions.

**Figure EV3:**
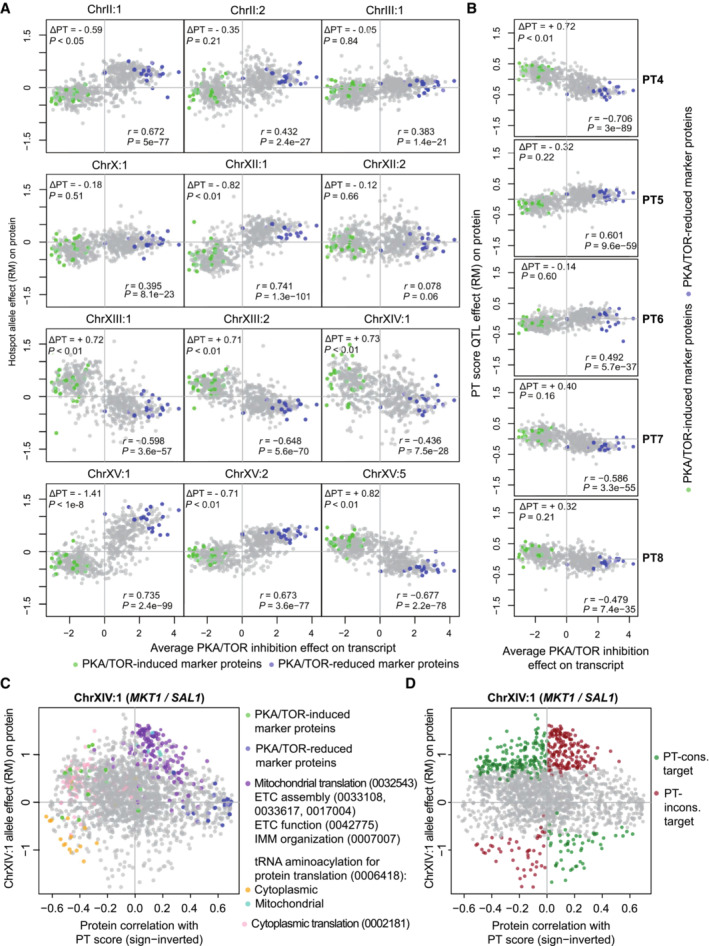
Proteome‐wide comparisons of pQTL effects to the effects of chemical inhibition and functional delineation of effects at pQTL hotspot ChrXIV:1 Comparison of protein abundance changes associated with allele differences at 12 pQTL hotspots to the effect of chemical inhibition of PKA and TOR signaling pathways on transcript abundance (Kunkel *et al*, 2019). The effect of chemical inhibition as shown here represents the average effect following 20 min inhibition of either pathway. Marker genes used for calculation of the PT score are highlighted.Same comparison as in panel A for genetic loci with strong PT score mapping signals and without accumulation of pQTL (see Fig 4A and main text).Comparison of protein abundance changes due to allelic differences at hotspot ChrXIV:1, which spans the *MKT1* and *SAL1* loci, to global correlation of respective protein abundances with the PT score across BYxRM segregants. The x‐axis shows the beta coefficient of the PT score as a predictor of protein abundance in a combined linear model with the genotype at the hotspot ChrXIV:1 locus to correct for the effect of the locus itself. Analysis by partitioning of proteins into GO terms as indicated.Same analysis as in panel C but coloring according to consistent (green) or inconsistent (red) direction of protein abundance change with allelic difference at the ChrXIV:1 locus compared to the expected direction based on the PT score difference at this locus.

To test this proposed classification, we aimed to assess the spread of genetic variant effects by comparing distances of pQTL target proteins in a physical protein interaction network (STRING, Szklarczyk *et al*, 2021). As a proof‐of‐concept, we focused on a pQTL hotspot on Chromosome X (“ChrX:1”), which did not show a significant PT score difference (−0.18, *P* = 0.5, Fig EV3A). Among 20 proteins with a significant pQTL linking to this hotspot, several were reported to be targets of the zinc‐responsive transcription factor Zap1 (Adh4, Tsa1 and Lap3; Lyons *et al*, 2000; Wu *et al*, 2008). *ZAP1*, which coincided with the hotspot, contains 9 missense variants between BY and RM. The hotspot hence likely affected a functional module related to the sensing of zinc while PKA and TOR signaling remained unaffected. To quantify the spread of these genetic effects, we computed distances between all pairs of target proteins of the *IRA2* and *ZAP1* loci as the shortest path by which these proteins are connected in the interaction network (Fig 4C). These distances were measured as the number of “edges” or reported protein–protein interactions. Network distances between targets of the *ZAP1* locus were on average shorter (3.02 edges per pair, 153 pairs) than those between targets of the *IRA2* hotspot (3.46, 19,503 pairs, *P* < 0.01 assuming a Poisson distribution for inter‐node distances, Fig 4C). Thus, although variants at the *ZAP1* locus affected many proteins, those effects remained confined to a specific network module and were therefore classified as “regional.”

Since the spread of the effect of a genetic variant across the network might be related to effect strength or the number of targets, we next compared pair‐wise distances for the targets of 265 pQTL clusters across the genome (see Materials and Methods) to the respective number of targets. Additionally, we determined the distribution of network distances by repeatedly drawing random samples of various sizes (Fig 4D). The average network distance for all 7 clusters with more than 50 targets (except *IRA2*) fell below the median of the distribution of average network distance for the respective random sample size. This is consistent with the expectation that the targets of an individual QTL are functionally related and therefore should be comparably close to each other in a network. However, the cluster containing the *IRA2* locus was an exception: the mean network distance among its 165 targets was 3.36 edges, which was greater than expected based on random samples of the same size (0.95 percentile for 10,000 samples: 3.28 edges). Hence, consistent with its classification as “global,” the *IRA2* locus affected distant parts of the molecular network. The opposite was true for all other clusters with many targets: We concluded that, as expected, most pQTL hotspots affected proteins in relatively close proximity, prompting classification of their effects as “regional.”

On the other hand, 8 of 12 pQTL hotspots (as detected in Grossbach *et al*, 2022) showed significant PT score differences (*P* < 0.05, Student's *t*‐test) and their proteome‐wide effects often correlated with the effects of chemical inhibition of PKA and TOR (Fig EV3A). Hence, significant shifts of the PT network state and other central signaling pathways may result as a secondary consequence of these strong perturbations. Particularly striking in this regard was the *MKT1/SAL1* locus (PTQTL2). The average network distance among 320 targets of the respective pQTL cluster was only 2.86 edges (0.05 percentile of random samples: 3.07). The higher PT score in strains carrying the RM allele (+0.63) at this locus was expected to result in a lower abundance of mitochondrial proteins (cf. GO slim term “cellular respiration,” Fig 3A) but instead, it was accompanied by a relative increase (Fig EV3C). This prompted us to split the proteins affected by this locus into “PT‐consistent” and “PT‐inconsistent” groups (Fig EV3D). The PT‐inconsistent targets were located in closer proximity in the network than PT‐consistent targets (Fig 4C), suggesting a primary regional effect on mitochondria and a secondary global effect via PKA/TOR signaling. The proteomic differences observed at this pQTL hotspot are further detailed in Appendix Text 2 and Fig EV3C and D.

Next, we asked whether global effects were limited to strong perturbations, that is, loci with many molecular targets. To address this question, we quantified the spreading of effects in the network emerging from genetic perturbations with small effect sizes. When the FDR threshold of the QTL mapping was relaxed to 0.42 five loci with low numbers of pQTL could be identified (“PT4” ‐ “PT8,” Fig 4A). Even after grouping similar markers with almost identical alleles across the individual strains (grouping markers in Linkage Equilibrium above 0.8), no more than six proteins were found to be significantly associated with any of these loci. Thus, PT4 ‐ PT8 can be regarded as small effect loci. However, subtle changes across the proteome were elicited by allelic differences at PT4 ‐ PT8 and these were found to be correlated with the effects of chemical inhibition of PKA and TOR (Fig EV3B). This suggested that these loci affected a similar set of proteins as the *IRA2* PTQTL, albeit in a more subtle manner. We computed the average network distance between a variable number of most affected proteins (top 50, 100, 200, or 320; irrespective of their statistical significance) at each locus. As illustrated in Fig 4D, the average distance between these proteins mostly fell above the median for random samples of the same size (16 of 20 data points) and it exceeded the 0.95 percentile in two cases (50 most affected proteins at PT5 and PT7). The average distances among targets of pQTL hotspots (7 clusters with more than 50 targets, except *IRA2*) were significantly lower than the average distances between PT4 ‐ PT8 target proteins (*P* < 0.01 for individual Student's *t*‐tests performed for each “top N”). Together, this indicates that loci with low or moderate effects on the PT score can have subtle, but “global” effects on the molecular network.

The global character of PTQTL effects suggested that two proteins that are under the common control of the PT network state can be correlated even if they are only distantly related in the network. To test this, we computed pair‐wise correlations among all proteins and binned all pairs by network distance. Next, we quantified to what extent a correlated pair of proteins (|*r*| > 0.5) was under the control of (or associated with) the PT network state. To quantify this, the correlation of the individual proteins with the PT score was calculated. For anti‐correlated pairs, one of the correlation coefficients was sign‐inverted. Finally, the absolute value of the mean of both individual correlations served as a measure of “compatibility” with PT network state alteration as a driver of the correlation for each protein pair. This analysis revealed that correlated protein pairs falling into low distance bins (bins 1–3) were less correlated with the PT score (|mean (sign‐corrected) *r* of individual (paired) proteins with PT score| > 0.5 for 46% of 48,722 pairs) than protein pairs in higher distance bins (71% of 10,892 pairs, *P* < 1e−15, Fisher's exact test). Furthermore, anti‐correlated protein pairs were more often associated with PT score differences than correlated protein pairs and anti‐correlation was more prominent in higher network distance bins than in lower network distance bins (Fig 4E). Hence, the proportions of correlation and anti‐correlation among targets of a QTL might be helpful to characterize genetic effects in terms of their spread across the network.

In sum, we were able to differentiate the effects of pQTL according to the relatedness or distance of their targets in a physical interaction network. Specifically, comparison of the network localization of proteome‐wide effects to changes in the PT network state allowed us to develop mechanistic hypotheses for the occurrence of global as compared to regional and local network effects caused by genetic variation (Fig 4F). We concluded that substantial changes of the PT score were associated with changes in diverse functional modules and a re‐organization of many basic cellular functions (Fig 3). This re‐organization was in part due to anti‐correlated changes among distant protein pairs (Fig 4E).

### Gene–environment interactions shape the PT network state

Previous studies have noted widespread gene–environment interaction for variants that influence gene expression in the BYxRM cross (Smith & Kruglyak, 2008). To evaluate environmental influences on network effects of genetic variation, we investigated PT score changes in the BYxRM cross across two extreme growth conditions: growth on glucose against growth on ethanol.

**Figure 5:**
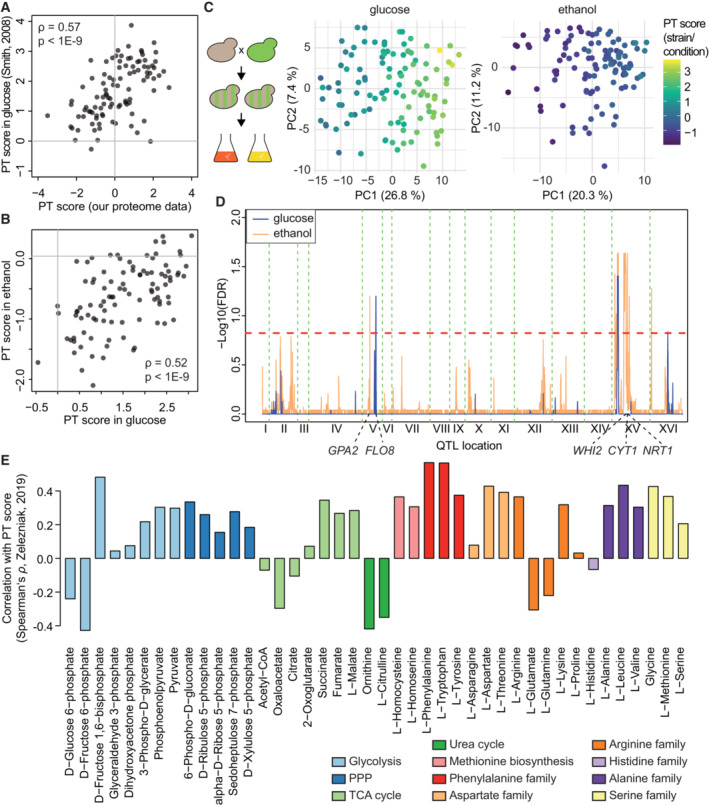
The PT network state is subject to gene–environment interaction Comparison of PT score based on our proteome data and PT score based on transcriptome data from (Smith & Kruglyak, 2008) for 99 strains grown on glucose and present in both datasets.Distribution of PT scores for 109 strains grown in glucose (left) or ethanol (right) based on transcriptome data from (Smith & Kruglyak, 2008).PCA of transcriptome variability for 109 strains grown in glucose or ethanol as indicated. Calculation of Eigenvalues, coloring by PT score, and labeling as in Fig 2A.QTL mapping of PT score for 99 strains grown in glucose (blue) and ethanol (orange). 15% FDR threshold indicated by dashed red line.Correlation of PT score based on proteomic data for several kinase KO strains with metabolite measurements (Zelezniak *et al*, 2018). Source data are available online for this figure.

First, we re‐analyzed transcriptome data generated previously for strains of the BYxRM collection grown on glucose and on ethanol (Smith & Kruglyak, 2008) and calculated a PT score based on marker transcripts (see Materials and Methods) for each of 109 strains in both conditions. Across 99 strains for which proteome data was available in our own dataset, PT scores showed good agreement (Spearman's *ρ* = 0.57, *P* < 1E−9, Fig 5A), confirming that the PT score was consistent across independent studies and different molecular modalities. Notably, PT scores of strains grown in glucose and ethanol were mostly positive and negative, respectively, indicating a strong effect of the growth condition (Fig 5B), while PT scores of the same strains were correlated between the two conditions (*ρ* = 0.52, *P* < 1E−9). Further, transcriptome variability was correlated with the PT score under both growth conditions: PCA analysis of transcriptomes across 109 strains revealed a strong correlation between PC1 scores and PT scores in glucose (adj. *R* ^2^ = 0.91, Fig 5C). The correlation was slightly reduced for strains grown on ethanol (adj. *R* ^2^ = 0.86) and PC1 reflected a smaller proportion of total transcriptome variance (Fig 5C), in line with the PT scores spanning a smaller range compared to the glucose condition (Fig 5B).

We next performed QTL mapping for PT scores of strains grown on glucose and ethanol as above. The locus close to the *IRA2* hotspot harbored a significant PTQTL in both conditions (Fig 5D). However, its effect was significantly weaker during growth on ethanol (*P* < 0.01 for the interaction term in a combined linear model), consistent with previous reports (Smith & Kruglyak, 2008). Another PTQTL for strains grown on glucose was found within 3 kb to the *FLO8* gene on Chromosome V that carries a null mutation in the BY progenitor strain S288c (Liu *et al*, 1996). Several PTQTL for strains grown on ethanol were close to genes that are known to be relevant for PKA or TOR signaling and cellular fitness, especially in conditions of nutrient scarcity. These include the *GPA2* gene on Chromosome V and *WHI2*, *CYT1*, and *NRT1* on Chromosome XV (Fig 5D). Whi2 negatively regulates TORC1 and Ras/PKA activity under nutritional stress (Leadsham *et al*, 2009; Chen *et al*, 2018; Teng & Hardwick, 2019), cytochrome C1 (gene *CYT1*) is essential for respiration and finally, *NRT1* encodes a high‐affinity nicotinamide riboside transporter (Belenky *et al*, 2008). In sum, this analysis demonstrated that the PT score correlated with molecular network effects of genetic variation in multiple growth media in a condition‐dependent manner and that large‐scale transcriptome variation was associated with the PT score under those conditions.

### The PKA/TOR network state correlates with an anabolic pattern in kinase KO strains

Our analysis of proteomic changes associated with PT state differences indicated strong links to the central energy and amino acid (AA) metabolism (Fig 3A), which aligns with the known functions of PKA and TOR signaling. PKA activity is subject to sensing of external and internal metabolic cues, most notably the levels of glucose and cAMP, respectively (Conrad *et al*, 2014), whereas the activity of TOR is strongly dependent on the sensing of intracellular and extracellular AA levels (Hara *et al*, 1998; Shimobayashi & Hall, 2016; Gonzalez & Hall, 2017). In order to further investigate associations between the PT network state and the metabolic configuration of the cell, we re‐analyzed proteomic and metabolomic data for 22 kinase KO strains (datasets 2 and 3 in Zelezniak *et al*, 2018) and compared PT scores calculated based on proteomic data to metabolite levels (Fig 5E). We observed that 15 out of the 18 proteinogenic AAs measured showed positive correlation with the PT score, with notable exceptions being the levels of glutamate (Glu, *ρ* = − 0.31) and glutamine (Gln, *ρ* = − 0.22). This may seem surprising, since addition of Gln promotes strong activation of TOR signaling (Duran *et al*, 2012; Oliveira *et al*, 2015). However, our analysis differs from studies employing the addition or removal of extracellular Gln as a nitrogen source in that it investigates the metabolic state across strains in which PKA and TOR activity differ due to genetic perturbation. Since Glu and Gln are essential nitrogen donors for the synthesis of both nucleotides and AAs, they will be rapidly used in highly proliferating cells. Thus, in this setting, intracellular concentrations of Gln and Glu might be lower in strains with high PT scores due to more active proliferation. Our observation is also consistent with earlier reports that TOR activity represses Glu and Gln biosynthesis via inhibition of the retrograde signaling pathway (Dilova *et al*, 2004). A particularly strong correlation was observed between the PT score and fructose‐1,6‐bisphosphate (*ρ* = +0.48), which is in line with the decisive regulatory role of this metabolite for Ras/PKA and as an indicator of glycolytic flux (Peeters *et al*, 2017; Zhang & Cao, 2017; Tanner *et al*, 2018). Finally, we observed positive correlation of the PT score with all intermediates of the pentose phosphate pathway (*ρ* = +0.15 to +0.28). In sum, our analysis confirmed a tight association between the PT network state and cellular metabolism. In particular, PT activity seemed to be linked to an anabolic and proliferative state of the cell.

### The PKA/TOR network state explains molecular and phenotypic diversity in distantly related yeast species

We next asked whether a similar network state exists in different species and focused first on the distantly related fission yeast *Schizzosaccharomyces pombe*. We previously observed differences in stress resistance and longevity between a natural isolate, Y0036 (Y0) and the common *S. pombe* lab strain Leupolds968 (L9) (Clement‐Ziza *et al*, 2014). We also noticed increased stress resistance across several conditions in the industrial strain DBVPG2812 (DB, unpublished observation). To explore the genetic underpinning of that phenotypic diversity, we established a three‐way cross between these parents (R1 = Y0 × L9, R2 = L9 × DB, and R3 = DB ×Y0) with 43–45 segregants per cross and collected transcriptomic data for the parental strains and each segregant during exponential growth on standard medium. Furthermore, we also collected these data for populations grown in the presence of 0.5 mM hydrogen peroxide (H_2_O_2_) for 1 h. Oxidative stress constitutes a naturally occurring stress that plays a decisive role for the network state re‐organization at the transition to respiratory growth (Tran *et al*, 2019).

**Figure 6:**
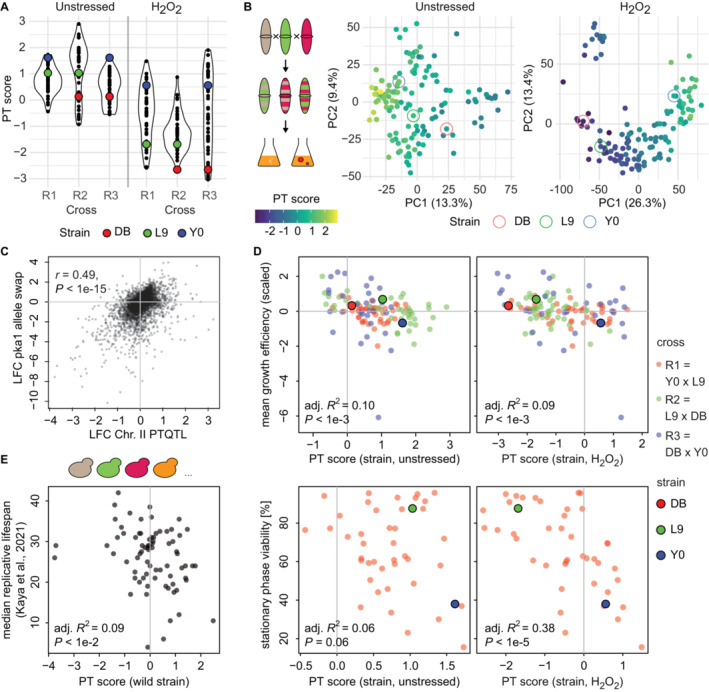
PT score explains molecular variability and cellular fitness traits in distant yeast species Range of PT score values across pair‐wise crosses (R1 = Y0 × L9; R2 = L9 × DB; R3 = DB × Y0) and in two conditions. Parental strains highlighted as indicated.PCA based on transcriptome variability for 127 segregants and 3 parental strains as indicated, in unstressed (left) or H_2_O_2_‐stressed condition (right). Coloring by PT score and labeling as in Fig 2A.Comparison of pka1 DB‐allele effect in an L9 allele‐swapped strain to differences due to the DB allele at the PTQTL on Chromosome II in the 3‐way cross for all transcripts detected in both settings.Correlation between growth efficiency and PT scores for 114 strains (upper panels) and comparison between stationary phase viability and PT scores for strains in the R1 cross (lower panels). PT scores were determined for samples of each strain grown either in the presence or absence of H_2_O_2_. Coloring by cross and parental strains enlarged as indicated.Comparison between median replicative lifespan and PT score for 75 wild isolate yeast strains based on data from (Kaya *et al*, 2021). Source data are available online for this figure.

The PT score for each segregant strain was calculated based on orthologs of the budding yeast PT score marker genes, during both unperturbed growth and in the presence of H_2_O_2_. The range of the PT score varied strongly between the individual pair‐wise crosses (R2 > R1 ≈ R3). Segregants in the R2 cross were assigned the highest median PT score (1.57) in the unstressed condition but the lowest median score (−1.27) in the H_2_O_2_ stress condition (Fig 6A). As previously observed in budding yeast, the PT score correlated with segregant scores along PC1 of the transcriptome in both conditions (adj. *R* ^2^ = 0.72 and 0.82, both *P* < 1E−15, Fig 6B).

**Figure EV4:**
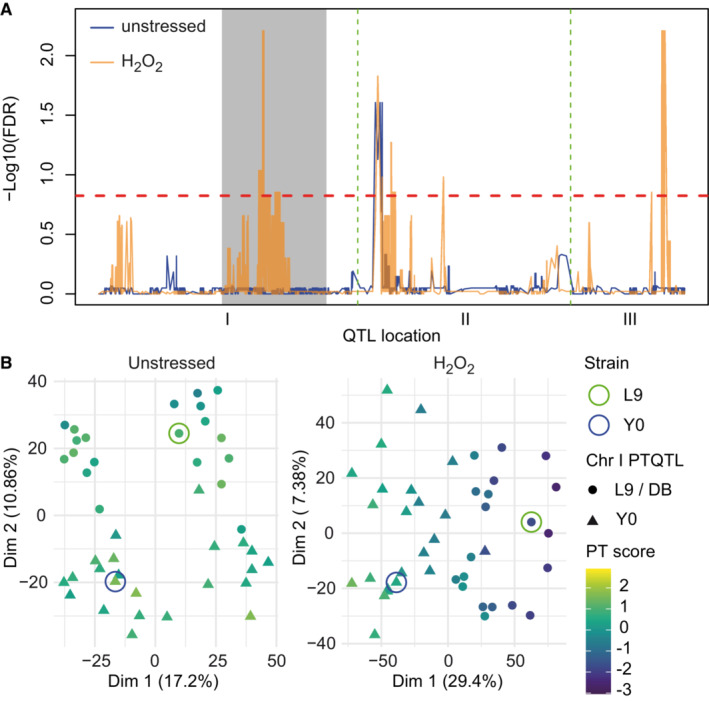
Supporting results for PT score‐based analysis of the fission yeast three‐way cross QTL mapping of the PT score in the fission yeast 3‐way cross in two conditions, as indicated. 15% FDR indicated by red dashed horizontal line. Shaded area represents inverted region of Chromosome I in the Y0 parental strain.PCA based on transcriptome variability for 43 segregants between the L9 and Y0 parental strains (cross R1, parental strains highlighted) in two conditions. Strains are colored by PT score and shape represents allele identity at the most significant marker of PTQTL1 for strains in H_2_O_2_.

Next, we performed QTL mapping to identify genetic determinants of the PT network state in fission yeast. In the unstressed condition, the strongest QTL was detected at an FDR of 0.15 (Fig EV4A). We investigated this locus and found a polymorphism at position 1,073 in the *pka1* allele of the DB parent that led to an amino acid exchange from cysteine to phenylalanine (C358F) compared to the other parents. Indeed, swapping the *pka1* allele in the lab strain L9 for the DB allele resembled the effects of this locus on the transcriptome (*r* = 0.49, *P* < 1E−15, Fig 6C). We still observed strong correlation between the PT score and segregant scores along PC1 in the group of segregants with the DB allele (adj. *R* ^2^ = 0.63, *P* < 1E−8). Hence, additional genetic variants likely contributed to variation of the PT network state in the unstressed condition but were beyond our limit of detection. QTL mapping in the H_2_O_2_ stress condition revealed significant signals in six distinct regions (Fig EV4A). One of these QTL coincided with the *pka1* locus and consistently, the *pka1* allele swap also partially reproduced the effects of this QTL across the transcriptome in the presence of H_2_O_2_ (*R* = 0.25, *P* < 1E−15). The strongest signal for PT score changes in H_2_O_2_ was attributed to a region on Chromosome I, within a large genomic inversion in the Y0 parental strain. The Y0 allele at the most significant marker led to higher PT scores in the presence of H_2_O_2_ (+1.43, *P* < 1E−6), but not during unperturbed growth (+0.13, *P* = 0.4) and contributed to the dominance of PT score‐related differences in global transcriptome variability of R1 segregants specifically in the H_2_O_2_ condition (Fig EV4B). This effect again illustrated the importance of gene–environment interactions underlying PT network state differences.

To evaluate the relevance of the PT score for cellular fitness in fission yeast, we recorded growth efficiency (final OD after 36 h of growth in unperturbed conditions) for 114 strains across the collection. This measure correlated significantly with both the strains' PT scores in unperturbed growth conditions as well as with PT scores for strains grown in the presence of H_2_O_2_ (Fig 6D, adj. *R* ^2^ = 0.10 and 0.09, respectively, both *P* < 1E−3). We further evaluated the predictive capacity of PT scores in fission yeast using stationary phase viability measurements obtained in a previous study for the R1 cross (Clement‐Ziza *et al*, 2014). As for the previous trait, we compared these measurements to PT scores from the unperturbed and oxidative stress conditions. In contrast to growth efficiency, we observed a strong negative correlation between stationary phase viability and the PT scores calculated from populations grown in the presence of H_2_O_2_ (adj. *R* ^2^ = 0.38, *P* < 1E−5) but only weak correlation with the PT scores based on unperturbed samples (adj. *R* ^2^ = 0.06, *P* = 0.06). As described above, we detected a major QTL that dominated PT score differences among strains in the R1 cross exclusively in the presence of H_2_O_2_. We speculate that such condition‐specific effects of genetic variance can contribute to fitness differences by eliciting PT network state differences. Such effects would be limited to fitness traits that are related to the conditions in which the genetic variant effects are penetrant.

Motivated by the successful application of the PT score as a predictor of cellular fitness traits in fission yeast, we applied it to replicative lifespan measurements recorded for 75 wild isolate strains of the yeast species *Saccharomyces paradoxus* and *S. cerevisiae* (Kaya *et al*, 2021). Based on the same sets of markers derived from chemical inhibition experiments as before, we calculated PT scores using the reported transcriptomic data and compared these scores to the replicative lifespan of the corresponding strains (Fig 6E). Indeed, PT scores were weakly, but significantly predictive of replicative lifespan (adj. *R* ^2^ = 0.09, *P* < 0.01) across these wild isolates.

We conclude that the PT network state explains long‐range network effects and fitness consequences of genetic variants across a range of growth conditions and in distantly related species of yeast.

## Discussion

In this study, we have investigated changes of “multi‐omics” molecular network states as a result of genetic variation in three yeast species during exponential growth across different environmental conditions. We observed that substantial fractions of transcriptome, proteome, and phospho‐proteome variability among genetically different yeast strains were correlated to a score based on markers of PKA and TOR pathway activity (PT score). The proteomic composition of strains with variable PT scores illustrated the widespread reorganization of the cellular molecular network by these central signaling pathways. We studied the genetic basis of the PT network state in combination with the network distance between QTL target proteins. This analysis showed that genetic variants associated with PT score differences caused (global) changes to distant parts of the network. Our work suggests a rationale for the conditions under which genetic effects have long‐range consequences in molecular networks: whenever distant network modules require a specific balance to maintain cellular homeostasis, long‐range effects can ensue. This concept will aid future genetic mapping and association studies because it provides a mechanistic explanation for why certain genetic effects are pleiotropic. Notably, our concept differentiates between pleiotropy in terms of effect size and pleiotropy in terms of the spread of genetic effects across the network. Although the state of PT signaling was the dominant network state alteration in the yeast crosses analyzed here, we do not imply that it would be the only regulatory mechanism explaining global effects of genetic variants.

PKA and TOR signaling have been established as central switches in cellular physiology across most eukaryotes (Gonzalez *et al*, 2020). In yeast, the PKA and TORC1 signaling pathways jointly coordinate the transition from fermentative to respiratory metabolism and regulate the activity of a wide range of cellular functions beyond growth, including intermediate and energy metabolism, transcription, mitochondrial and cytoplasmic translation, spore formation, and autophagy (Pedruzzi *et al*, 2003; Conrad *et al*, 2014; Workman *et al*, 2016; Gonzalez *et al*, 2020; Plank, 2022). Earlier studies have already provided examples for genetic variants that affect the PKA/TOR‐dependent metabolic balance during exponential growth, including mutations in fission yeast pyruvate kinase (Kamrad *et al*, 2020), but have not systematically explored its genetic basis. We did not specifically investigate the role of TORC2 (Plank, 2022) or AMPK/Snf1 signaling (Zaman *et al*, 2009; Kingsbury *et al*, 2015; Malecki *et al*, 2020) for the program reflected by the PT score but do not exclude a potential influence. Our analysis of the association of different cellular functions (GO terms) with the PT network state at the proteome and phosphoproteome level confirmed the coordinated adjustment of a wide range of functional modules to the metabolic state of the cell. Importantly, here we did not study the targets of a specific pathway or perturbation but rather correlations with a global network state, shifted by natural genetic variation.

It is remarkable that the molecular patterns of the two parental strains in the BYxRM collection are very similar despite widespread effects of the *IRA2* locus. Our analysis shows that other pQTL hotspots including *BUL2* and variants with smaller effects together balance the effect of the *IRA2* locus. This observation provides exciting testable hypotheses for future research: drugs (such as rapamycin) should be able to balance global effects as caused by the *IRA2* variant and therefore reverse a great diversity of genetic effects and possibly even disease phenotypes. Our model helps explaining the joined effect of multiple genetic variants and quantification of the PT network state even enabled the prediction of outcomes of genetic configurations without pinpointing individual variants. For example, a large, poorly resolved genetic region led to PT network state differences in a fission yeast cross (Y0036 × L968) under oxidative stress. PT network state differences in turn mediated the effects of genetic variation on a wide spectrum of cellular traits, including stress resistance (heat stress, oxidative stress) and replicative lifespan. This provides a new perspective on the integration of metabolic regulation with other cellular functions in the context of genetic variability, which has become a major focus of studies in the field of proteostasis and aging (Ottens *et al*, 2021).

We were able to discriminate between loci that caused changes to distant parts of the molecular network by modifying the PT network state and loci that had more confined consequences. Examples for the latter type of loci included pQTL with local effects on the abundance of individual proteins such as the effect on Hsp104 and a pQTL hotspot with regional effects on several downstream targets of the Zap1 transcription factor but without concomitant reorganization of multiple functional modules. The average distance across the molecular network between proteins regulated by genetic hotspots was in most cases smaller than observed in random samples, consistent with the expectation that most hotspots have strong effects on specific functional modules. In contrast, the PT network state serves as an example of a—genetically controlled—network state associated with a global re‐organization of the cell. We detected several loci with comparably weak effects (showing a low number of significantly affected protein targets) but detectable differences in the PT network state. The most affected proteins at these loci displayed larger network distances than the targets of most hotspots. Hence, weak effects can be pleiotropic in the sense that they result in changes in distant parts of the molecular network. Effect size and network spread are therefore not strictly coupled.

Among proteins whose abundance correlated with the PT network state, anti‐correlation was more frequent than among proteins which correlated independently of the PT network state. Network‐based statistics such as “betweenness” could further serve as quantifiable criteria to determine the type of genetic effects in an *a priori* manner when individual variants and hence, variant genes can be identified. Unfortunately, the genetic resolution of our data was insufficient to confidently assign sufficient numbers of QTL to individual variants or genes to allow further analysis in this direction. It is also important to note that there may be no objective, quantifiable boundaries separating local from regional and regional from global effects. Instead, we expect a continuum of network changes and the terms “local,”, “regional,”’ and “’global”’ should help to conceptualize the different nature of QTL effects on molecular networks. For example, we propose that far‐reaching genetic effects, such as those postulated in the concept of “omnigenic inheritance” (Boyle *et al*, 2017), may originate from adjustments in cellular physiology, caused by genetic variants with global effect components.

Our work provides a schema for causally linking genetic variation to general physiological programs: it begins with the identification and quantification of network states that serve as low‐dimensional, biologically interpretable representations of complex cellular states, proceeds by characterizing their cellular function, analyzing genetic and environmental mechanisms affecting them, and ultimately synthesizing that information to explain differences between individuals. By developing molecular signatures that faithfully capture a particular network state, we will be able to interpret transcriptomic data (such as those collected by the GTEx consortium) in quantitative physiological terms and to explain the convergence of hitherto seemingly unrelated genetic variation on more general regulatory programs. Exemplarily, we confirmed conservation of the PT network state between *Saccharomyces cerevisiae* and *Schizzosaccharomyces pombe* despite millions of years and different paths of evolution that separate these species (Rhind *et al*, 2011). In mammalian cells, the transition between respiratory and glycolytic metabolism is known as the “Warburg effect” (Liberti & Locasale, 2016), which was first observed in carcinogenesis but has since been described in many other circumstances such as cellular differentiation (Zhang *et al*, 2018; Bhattacharya *et al*, 2020) and aging‐associated disease (Traxler *et al*, 2022). For example, we have recently detected an association between ERK signaling (the mammalian equivalent of the PKA pathway) and the co‐occurrence of aging‐associated diseases (Fraser *et al*, 2022). Here, we have shown the utility of quantifying network states for explaining the consequences of genomic variability for cellular traits. We propose to test other regulatory mechanisms that exert developmental or metabolic control for network state‐regulating properties and to chart their natural genetic variation. This framework will help to better understand (i) the polygenicity of complex traits, (ii) the propagation of genetic effects in molecular networks, and thereby (iii) pleiotropic effects.

## Materials and Methods

### Heat‐stress experiments

The BYxRM yeast strain collection was originally derived from a cross between the two parental strains BY4716, an S288C derivative (*MAT*α lys2Δ0) and RM11‐1a (*MAT*a leu2Δ0 ura3Δ0 ho::KAN; Brem *et al*, 2002). Experiments to determine thermotolerance were performed for a subset of 100 segregants, which were selected based on non‐flocculent growth and by the availability of genotype data from our previous study (Grossbach *et al*, 2022), and for the parental strains. Experiments on the parental strains were performed alongside segregant experiments on three separate days to assure consistent results over the entire course of the experiments. Pre‐cultures in synthetic complete medium with 2% glucose (FORMEDIUM) were inoculated from YPD plates grown for 3 days after thawing of yeast stocks. Over‐night cultures in the same medium were inoculated from the pre‐cultures and were grown with constant shaking at 30°C in glass flasks. Heat treatment was applied to samples of cells taken at OD_600_ between 0.7 and 0.8 in PCR tubes in a PCR cycler. Temperature was increased from 25 to 45°C by 0.1 K/s with subsequent incubation at 45°C for 8 min. As a control, samples from the same cultures were incubated at 25°C. 60 μl of the samples were then diluted 1:30 into fresh medium in 24‐well plates. Growth curves following dilution after mock or heat treatment were recorded in a Thermo Fisher Varioskan Flash instrument with further incubation and shaking at 30°C. Growth curves were fitted using the R package “grofit” (Kahm *et al*, 2010) with a parametric fit following a Richard's law model to infer growth characteristics.

### QTL mapping and trait mapping based on molecular data in budding yeast

QTL mapping to identify genetic determinants of heat‐induced lag and the PT score in budding yeast was performed using Random Forest (RF)‐based machine learning as described previously (R package “RFQTL” and Grossbach *et al*, 2022). We used a similar procedure to determine protein abundances that have predictive power for heat‐induced lag. In brief, scaled protein abundance values were used as continuous predictor variables to grow RF models of heat‐induced lag measurements from 102 strains, including averaged triplicate measurements for the parental strains. Statistical significance of the results was assessed by permutation of heat‐induced lag measurements across the strains to generate a null distribution of variable importance for each predictor. *P*‐values were adjusted for multiple testing using a Benjamini‐Hochberg procedure.

QTL mapping of the PT score based on data reported in (Smith & Kruglyak, 2008) was performed on a set of 99 segregants, which we previously genotyped based on our own RNA‐Seq data (Grossbach *et al*, 2022).

### PT score calculation

We defined sets of PT‐regulated transcripts by setting a threshold of at least two standard deviations for the effects of chemical inhibition (Kunkel *et al*, 2019). Here, we included data at both the 20 and 150 min time points for both PKA and TOR inhibition. After exclusion of transcripts that changed abundance in the opposite direction in any of these treatments, we retained 47 (of 65) transcripts as a PT‐induced set and 44 (of 90) transcripts as a PT‐reduced set. In our data, abundance measurements for 22 and 18 corresponding proteins were available. A PT score was then calculated for each of 112 strains for which proteomic data was available (Grossbach *et al*, 2022) as the difference between the median abundance of proteins in the induced set to the median in the reduced set using protein abundances after normalization and centering across all strains. Thus, a higher PT score corresponds to higher median abundance of PT‐induced proteins relative to PT‐reduced proteins. The same procedure was applied to calculate PT scores for 109 segregants based on transcript abundance data reported in (Smith & Kruglyak, 2008), for 96 kinase KO strains based on proteome data reported in (Zelezniak *et al*, 2018) and for transcriptome data for 133 strains from our three‐way cross in fission yeast.

### Analysis of phospho‐proteome correlations

Since in many cases several phosphopeptides were measured per protein, we considered the peptide that was best predicted by the PT score (highest adj. *R* ^2^) to reflect the functional state of the corresponding protein. This decision introduces a bias for proteins with high numbers of phosphopeptides and might in some cases overestimate the “net correlation” between a specific protein and the PT score. To mitigate the second and potentially more severe bias, we tested each protein for inconsistent predictions by the PT score. For 97 proteins that contained more than one significantly predicted peptide (FDR < 0.05), inconsistent predictions were found in only 13 cases (13.4%). This fraction was only slightly affected by the choice of the FDR threshold (15.8% inconsistent among 120 proteins at FDR < 0.25). We did not exclude these proteins from the following analysis, since they are unlikely to affect correlations across entire GO terms.

### Network distance analysis and clustering of pQTL

To evaluate the distance of a pair of proteins across the molecular network, we applied network graph analysis using the R package “igraph” (version 1.4.2, Csardi & Nepusz, 2006). The network graph was based on STRING database physical interactions at medium confidence level (score > 0.400; Szklarczyk *et al*, 2021). The graph was simplified by removal of multiple edges. Shortest path distances between protein pairs were calculated using the “distances” function from the igraph package. For the comparison of target network distances to the number of targets across pQTL, an adjacency‐restricted clustering algorithm described in (Ambroise *et al*, 2019) as implemented in the R package “adjclust” (version 0.6.6), was used to group pQTL. The number of clusters (k = 294) was chosen to result in an LD of 0.9 or higher within each cluster. Lead markers were defined for each cluster based on the number of targets assigned to each marker within the cluster. Clusters were further merged if the LD between any lead marker between two adjacent clusters exceeded 0.9. This resulted in a final set of 265 clusters.

### Generation of a three‐way fission yeast cross

Parental strains for the three‐way cross in fission yeast were chosen based on differences in their resistance to oxidative stress in preliminary experiments. The three parental strains were JB50, a strain closely related to the reference strain JB22 (Leupolds968, L968), JB759 (Y0036), a strain with increased sensitivity to oxidative stress compared to JB50, and JB760 (DBVPG2812), a strain that is more resistant to oxidative stress than JB50. We used a double selection strategy to obtain diploid hybrids between each pair of parental strains. Specifically, we deleted the *ade6* locus in each parental strain and differentially replaced it with resistance genes for either kanamycin (KAN, in JB50) nourseothricin (NAT, in JB759) or hygromycin B (HB, in JB760). Plates containing both fungicides corresponding to a pair of parental strains were used to select for diploid hybrids carrying both resistance genes. F1 haploid recombinants were obtained straight from the selected diploid hybrids by tetrad analysis. F2 haploid recombinants were obtained by performing tetrad analysis of F2 diploid hybrids obtained from a mass mating among F1 haploid segregants. The cross included 150 strains in total.

### RNA‐seq analysis of fission yeast strains

Samples for transcriptomic analysis were grown in 50 ml YES medium to an OD_595_ of 0.4–0.5 at 32°C. These samples were either harvested before or after the exposure to 0.5 mM H_2_O_2_ for 1 h. In total, the transcriptomes of 286 samples, corresponding to 130 strains in two conditions were quantified. RNA isolation, library preparation, and sequencing were performed as described previously (Clement‐Ziza *et al*, 2014). We mapped reads against the Schizosaccharomyces pombe reference genome using Bowtie with the following parameters: ‐C ‐n 3 ‐e 100 ‐best (v.0.12.7, Langmead *et al* (2009)). Read group information was added, and BAM files were sorted using Picard utilities (http://broadinstitute.github.io/picard). Further processing of the RNA‐seq data was performed with the GATK pipeline (v3.4‐46‐gbc02625), according to best practice guidelines (Van der Auwera *et al*, 2013). Variants at the polymorphic sites were then called with UnifiedGenotyper. If the GATK score for a given site was below 20, the position was considered as unknown. We excluded polymorphic sites for which (i) the samples of the parental strains could not be called, (ii) or more than 50% of the segregants could not be called, or (iii) the minor allele frequency was less than 10% in our cross. We also excluded genetic markers if they were called differently than both closest neighbors (i.e. the nearest upstream marker and the nearest downstream marker) and those markers were within 50 kb. When possible, missing genotypes were inferred through the neighboring markers if they had an identical segregation patterns and were within 50 kb. In these cases, the marker was assigned the same allele as the two flanking markers. VCFs were converted to strain‐specific FASTA files using the GenomeGenerator tool (Clement‐Ziza *et al*, 2014).

For quantification, we mapped reads with Bowtie (v.0.12.7, Langmead, 2010) against the strain‐specific genomes we generated based on the RNA‐seq data using the following options: ‐C ‐‐best ‐m 1. Genes to which no reads were mapped in at least 50% of samples were dismissed. To correct for differences in library size, we compute a correction factor for each sample. We determined the 20% and 80% quantiles for each sample i and computed the median M_i_ for all counts between these quantiles. The counts for gene j and sample i were then multiplied with the ratio of the median of the counts for this subset of genes and the mean of these medians across all samples to correct for different library sizes. For replicate measurements, counts were averaged.

### Growth profiling of fission yeast strains

Growth profiling of 150 strains of the three‐way fission yeast cross was performed using the bioLector system (m2p‐labs, Germany) as described previously (Clement‐Ziza *et al*, 2014). The growth of each sample was determined by light scattering in 3‐min intervals for at least 25 h and then converted to optical densities with a linear model. Growth efficiency was calculated as the difference between the initial and final OD. The strains were distributed over 28 batches. We corrected for batch effects by subtracting the mean of all measurements for the batch from each of these measurements. Afterwards, we employed a step‐wise procedure to remove batches that showed much more variation than the rest of the batches. First, we computed the variance of all trait values per batch. Second, if any batch had a variance that was 2.5 times as large as the average variance of all batches, we removed the batch with the highest variance. Then we repeated this step until the variance for no batch exceeded the set threshold above the variance of the remaining batches. We removed four batches with this procedure, resulting in at least one measurement of growth efficiency for 114 strains. Repeated measurements per strain were averaged for analysis.

### Generation of a pka1 allele‐replacement strain in fission yeast strain JB50

To investigate the effects of the polymorphism in pka1 (C358F between JB50 and JB760), we generated an allele replacement strain. This strain was identical to JB50 aside from position 1,073 in the coding sequence of pka1, changing the respective codon from UGU, coding for cysteine, to UUU, coding for phenylalanine. The strain was generated using a CRISPR/Cas9 method as described before (Rodriguez‐Lopez *et al*, 2016) using the primer pair 5′‐acataacctgtaccgaagaaAGCAACTGTTGTACTCTTTGgttttagagctagaaatagc‐3′ (forward) and 5′‐gctatttctagctctaaaacCAAAGAGTACAACAGTTGCTttcttcggtacaggttatgt‐3′ (reverse). The replacement strain is referred to as PKA1Rep in the following. We used Sanger sequencing to validate successful introduction of the JB760‐allele of pka1 in the JB50‐background. To measure the effects of the pka1 allele‐replacement on the transcriptome and proteome, we grew three replicates each of JB50 and PKA1Rep under normal growth conditions and three replicates, for each strain, which were exposed to increased oxidative stress (0.5 mM H_2_O_2_) for 1 h before the samples were harvested. Each sample was separated into a fraction for RNA extraction and one for protein quantification. The fraction for protein quantification was centrifuged and the pellets washed with cold PBS and snap‐frozen in liquid nitrogen for transport.

### Transcriptome quantification for the JB50 pka1 replacement strain

RNA was extracted with the hot phenol method described in (Lyne *et al*, 2003). RNA was further purified with Qiagen RNAeasy columns, and DNAse treatment was performed in the columns (as suggested by manufacturer) prior to library preparation. RNA quality was assessed with a Bioanalyzer instrument (Agilent, United States), and all samples had a RIN (RNA Integrity Number) > 9. cDNA libraries were prepared with the Illumina TruSeq stranded mRNA kit, according to the manufacturer's specifications, by the Cologne Centre for Genomics (CCG) facility. The samples were sequenced on a single lane of an Illumina Hiseq4000 to produce stranded 2 × 75 bp reads. Reads were trimmed with Trimmomatic (v0.36, Bolger *et al*, 2014), with the following parameters differing from default settings: LEADING:0 TRAILING:0 SLIDINGWINDOW:4:15 MINLEN:25. The reference genome was indexed with bowtie2‐build with default settings. Paired reads were aligned to the reference genome using bowtie2 with default settings (v2.3.4.1, Langmead & Salzberg, 2012). In the case of the allele replacement strain, the reference genome was edited to reflect the base substitution within pka1. Aligned reads were counted using intersect from the bedtools package (v2.27.1, Quinlan & Hall, 2010), with the parameters ‐wb ‐f 0.55 ‐s ‐bed. Identical reads were only counted once. Read counts were tested for differential expression between strains using DESeq2 v1.18.1 with default settings (Love *et al*, 2014). We tested differential expression between strains separately with or without addition of H_2_O_2_.

## Author contributions

**Matthias Weith:** Conceptualization; formal analysis; funding acquisition; investigation; visualization; writing – original draft; writing – review and editing. **Jan Grossbach:** Software; investigation; methodology; writing – original draft; writing – review and editing. **Mathieu Clément‐Ziza:** Conceptualization; investigation; writing – original draft. **Ludovic Gillet:** Data curation. **María Rodríguez‐López:** Resources. **Samuel Marguerat:** Investigation; methodology. **Christopher T Workman:** Conceptualization; formal analysis; supervision; investigation; writing – original draft. **Paola Picotti:** Resources; supervision; writing – original draft. **Jürg Bähler:** Conceptualization; resources; supervision; writing – original draft. **Aebersold Rudolf:** Conceptualization; resources; supervision; writing – original draft. **Andreas Beyer:** Conceptualization; supervision; funding acquisition; methodology; writing – original draft; project administration; writing – review and editing.

## Disclosure and competing interests statement

The authors declare that they have no conflict of interest.

## Supporting information



Appendix

Expanded View Figures PDF

Dataset EV1

PDF+

Source Data for Figure 1

Source Data for Figure 2

Source Data for Figure 5

Source Data for Figure 6

## Data Availability

RNA sequencing data for the three‐way cross in fission yeast are available at the ArrayExpress repository with accession E‐MTAB‐12930 (http://www.ebi.ac.uk/arrayexpress/experiments/E‐MTAB‐12930).
