# Supplementary material for: Genetic effects on molecular network states explain complex traits
Source: Mol Syst Biol. 2023 Jul 24;19(8):e11493. doi: 10.15252/msb.202211493 (PMC10407735; doi:10.15252/msb.202211493)
Supplement: Supplementary file 1 — Appendix [file MSB-19-e11493-s009.pdf]

Appendix Text 1

We conducted an analysis of correlations between the PT score and proteins sorted by GO slim terms to leverage the availability of extensive -omics data on the collection of yeast strains derived from the BY and RM parental strains. The analysis charts the wide-spread adjustment of the molecular network along the axis defined by the PT score, which we consider to represent a fundamental adjustment of the network between the poles of fermentative versus respiratory metabolic preference.

Strong and highly directional correlations of protein abundance levels with the PT score comprised well-known targets of PKA and TOR signaling (Conrad et al., 2014), including cytoplasmic translation, ribosome biogenesis and related processes among positively correlated terms (Figure 3A). Carbohydrate transport and to a lesser extent amino acid transport were also positively correlated with the PT score, consistent with the role of glucose transport capacity for glycolytic activity (Elbing et al., 2004). We noted that transcription by RNA polymerase I and III but not II were among the positively correlated terms. Similar correlations have been reported recently for fission yeast cells grown on different carbon sources (Kleijn et al., 2022) and RNA pol III transcription seems to be required for glycolysis indicating a potential feedback loop with PKA or TOR signaling (Szatkowska et al., 2019). Moreover, we found relatively high prediction accuracy but variable directionality of correlation with proteins annotated for protein phosphorylation and dephosphorylation. These correlations may yield valuable insight into the regulation of kinases and phosphatase subunits.

Negative correlation was predominant within GO terms such as response to starvation and oxidative stress, carbohydrate metabolism, generation of precursor metabolites and energy, and sporulation. Cellular respiration was also among the terms that contained proteins with predominantly negative correlations with the PT score. However, the average predictive accuracy of the PT score for the abundance of these proteins was less than in other negatively correlated terms. This likely reflects the influence of strong genetic effects, such as those at the *MKT1*/*SAL1* locus (Figure EV1A, E and F) but also the effect of the *HAP1* hotspot (Brem et al., 2002) on mitochondrial genes in the BYxRM collection. While the different parental alleles at the *MKT1*/*SAL1* locus led to a highly significant change in the PT score (Figure EV3A, E and F), allelic differences at the *HAP1* hotspot did not display any directional effect on the PT score. Hence, a primary effect on mitochondrial function may or may not lead to a reorganization of the cellular configuration in terms of the PT network state, dependent on the timing, reach and character of the perturbation.

The strong negative correlation with the term “oligosaccharide metabolic process” included low levels of trehalose synthetase subunits in strains with high PT score, as expected. However,

we observed that isoforms of phosphoglucomutase differed in this regard: While Pgm2p was reduced with high scores, as expected (Howard et al., 2006), Pgm1p was significantly increased (LM beta = 0.49, q < 1E-14), hinting to an unknown role of this enzyme during growth on glucose. We also noted overall slightly positive correlation of the PT score with proteins annotated for transposition. Among these, RPC40, an RNA polymerase III subunit known for its role in transposon insertion (Bridier-Nahmias, 2015), was strongly associated with high PT scores (LM beta = +0.56, q < 1E-18).

We also analyzed the correlation of the PT score with protein phosphorylation data sorted by GO slim terms (Figure 3B). The most positive correlations between the PT score and absolute peptide phosphorylation levels were again found in terms related to cytoplasmic translation and ribosome biogenesis, indicating concurrent abundance and phosphorylation increase in this process. Phosphopeptides from proteins involved in cell budding (mean adj. R^2 = 0.21) and cytokinesis (mean adj. R^2 = 0.21) were more strongly correlated with the PT score than the respective protein abundances (mean adj. R^2 = 0.14 and 0.13, respectively). Consistently, we found that phospho-residuals belonging to proteins annotated with regulation of the cell cycle as well as processes known to show fluctuating phosphorylation through the cell cycle (Campbell et al., 2020), were positively correlated with the PT score. Interestingly, phospho-residuals of a number of proteins involved in dephosphorylation showed the highest mean correlation with the PT score.

The strong coverage of phosphorylation events across segregants in our dataset and interpretation in light of the PT score provided a wealth of information about many regulatory processes. As an example, we further scrutinized strong correlations between the PT score and phosphorylation events in proteins that are annotated for functions in histone modification. We noticed that several proteins involved in deposition, reading and removal of histone H3 lysine 36 (H3K36) methylation contained phosphopeptides that were strongly predicted by the PT score (Figure 3B). It has been reported that H3K36 trimethylation is important to prevent bi-directional (cryptic) transcription within gene bodies. In turn, the expression of nutrient-sensitive as well as stress response genes depends on the presence of the histone methylase Set2 (McDaniel and Strahl, 2017; Separovich et al., 2022). Set2 mediates co-transcriptional histone modification, which was further shown to depend on the cyclin-dependent kinase Bur1 and the Bur2 cyclin (Hossain et al., 2013). The process initiated by Set2p further involves recruitment of the Rpd3S histone deacetylase complex (Reim et al., 2020). Set2 interacts with the histone chaperone Spt6 and the Iws1/Spn1 component of the RNA polymerase II elongation complex and this interaction is crucial for the expression of highly transcribed mRNAs such as those encoding ribosomal proteins (Reim *et al.*, 2020). Conversely, demethylases Jhd1p, Jhd2p, Rph1p, and Gis1p remove H3K36 marks (Separovich *et al.*, 2022). We found strong correlations between the PT score and phosphopeptides in the BUR

kinase subunit Sgv1, Bur2, Set2, Iws1/Spn1 and its binding partner Spt6, the Sin3 component of the Rpd3S complex, Rph1 as well as Gis1. Furthermore, we found strong correlations with phosphopeptides in Chd1, a subunit of the SAGA complex, which recognizes H3K4 methylation via its chromodomain (Pray-Grant et al., 2005). Chd1 was also shown to bind to H3K36 methylated sites (Smolle et al., 2012) and loss of Chd1 alone or in combination with loss of Iws1/Spn1 resulted in cryptic transcription (Quan and Hartzog, 2010). Our observation that Chd1 phosphorylation strongly correlated with PT score variability supports recent reports that SAGA complex recruitment may be regulated in a TORC2-dependent manner (Cohen et al., 2022) and provides another intriguing link to transcriptional fidelity.

Appendix Text 2

Pairwise network distances between protein abundance targets of the cluster containing the *MKT1/SAL1* locus (coinciding with PTQTL2, Figure 4A) were surprisingly low given the significant change in the PT score at this locus (Figure EV3A and EV3D). Protein abundance changes due to the RM allele at hotspot ChrXIV:1 were globally anti-correlated with PKA and TOR inhibition ($r = -0.44$, $p < 1e-27$, Figure EV3A). However, we observed that mitochondrial proteins were not reduced as expected based on the PT score increase associated with the RM allele (Figure EV3E). Given the lower PT score in strains with the BY allele at this locus, we speculated that mitochondrial defects due to BY alleles of *MKT1* and *SAL1* (Dimitrov et al., 2009) led to reduced PKA or TOR activity following AMPK/Snf1 activation (Kingsbury et al., 2015; Malecki et al., 2020) or similar feedback mechanisms. Thus, we interpreted the effect of this hotspot as a combination of a regional effect on mitochondrial function and an additional effect via PKA or TOR signaling on a different set of cellular processes. To elucidate these effects, we partitioned the pQTL targets of this hotspot into two groups, depending on whether their abundance change was consistent ("PT-consistent", 216 proteins) or inconsistent ("PT-inconsistent", 210 proteins) with the difference in PT score due to the allele at this locus (Figure EV3F). Many (108/210) of the proteins in the PT-inconsistent group belonged to a limited set of functional modules including mitochondrial translation and electron transport chain function (Figure EV3E). Next, we calculated pair-wise shortest path distances within the PT-consistent and -inconsistent groups. The average distance between PT-consistent targets was larger (2.85 edges) than the average distance in the PT-inconsistent group (2.61 edges, $p < 0.001$, Figure 4C), suggesting that the former group of proteins belonged to more distant parts of the molecular network. Hence, we propose that the *MKT1/SAL1* locus had two distinct network effects: the first one resulting from long-range effects via the PT network state and a second one directly impinging on mitochondrial function without acting via PKA and/or TOR signaling.

Brem, R.B., Yvert, G., Clinton, R., and Kruglyak, L. (2002). Genetic dissection of transcriptional regulation in budding yeast. Science *296*, 752-755. 10.1126/science.1069516.

Campbell, K., Westholm, J., Kasvandik, S., Di Bartolomeo, F., Mormino, M., and Nielsen, J. (2020). Building blocks are synthesized on demand during the yeast cell cycle. Proc Natl Acad Sci U S A *117*, 7575-7583. 10.1073/pnas.1919535117.

Cohen, A., Pataki, E., Kupiec, M., and Weisman, R. (2022). TOR complex 2 contributes to regulation of gene expression via inhibiting Gcn5 recruitment to subtelomeric and DNA replication stress genes. PLoS Genet *18*, e1010061. 10.1371/journal.pgen.1010061.

Conrad, M., Schothorst, J., Kankipati, H.N., Van Zeebroeck, G., Rubio-Texeira, M., and Thevelein, J.M. (2014). Nutrient sensing and signaling in the yeast Saccharomyces cerevisiae. FEMS Microbiol Rev *38*, 254-299. 10.1111/1574-6976.12065.

Dimitrov, L.N., Brem, R.B., Kruglyak, L., and Gottschling, D.E. (2009). Polymorphisms in multiple genes contribute to the spontaneous mitochondrial genome instability of Saccharomyces cerevisiae S288C strains. Genetics *183*, 365-383. 10.1534/genetics.109.104497.

Elbing, K., Larsson, C., Bill, R.M., Albers, E., Snoep, J.L., Boles, E., Hohmann, S., and Gustafsson, L. (2004). Role of hexose transport in control of glycolytic flux in Saccharomyces cerevisiae. Appl Environ Microbiol *70*, 5323-5330. 10.1128/AEM.70.9.5323-5330.2004.

Hossain, M.A., Chung, C., Pradhan, S.K., and Johnson, T.L. (2013). The yeast cap binding complex modulates transcription factor recruitment and establishes proper histone H3K36 trimethylation during active transcription. Mol Cell Biol *33*, 785-799. 10.1128/MCB.00947-12.

Howard, S.C., Deminoff, S.J., and Herman, P.K. (2006). Increased phosphoglucomutase activity suppresses the galactose growth defect associated with elevated levels of Ras signaling in S. cerevisiae. Curr Genet *49*, 1-6. 10.1007/s00294-005-0036-z.

Kingsbury, J.M., Sen, N.D., and Cardenas, M.E. (2015). Branched-Chain Aminotransferases Control TORC1 Signaling in Saccharomyces cerevisiae. PLoS Genet *11*, e1005714. 10.1371/journal.pgen.1005714.

Kleijn, I.T., Martinez-Segura, A., Bertaux, F., Saint, M., Kramer, H., Shahrezaei, V., and Marguerat, S. (2022). Growth-rate-dependent and nutrient-specific gene expression resource allocation in fission yeast. Life Sci Alliance *5*. 10.26508/lsa.202101223.

Malecki, M., Kamrad, S., Ralser, M., and Bahler, J. (2020). Mitochondrial respiration is required to provide amino acids during fermentative proliferation of fission yeast. EMBO Rep *21*, e50845. 10.15252/embr.202050845.

McDaniel, S.L., and Strahl, B.D. (2017). Shaping the cellular landscape with Set2/SETD2 methylation. Cell Mol Life Sci *74*, 3317-3334. 10.1007/s00018-017-2517-x.

Pray-Grant, M.G., Daniel, J.A., Schieltz, D., Yates, J.R., 3rd, and Grant, P.A. (2005). Chd1 chromodomain links histone H3 methylation with SAGA- and SLIK-dependent acetylation. Nature *433*, 434-438. 10.1038/nature03242.

Quan, T.K., and Hartzog, G.A. (2010). Histone H3K4 and K36 methylation, Chd1 and Rpd3S oppose the functions of Saccharomyces cerevisiae Spt4-Spt5 in transcription. Genetics *184*, 321-334. 10.1534/genetics.109.111526.

Reim, N.I., Chuang, J., Jain, D., Alver, B.H., Park, P.J., and Winston, F. (2020). The conserved elongation factor Spn1 is required for normal transcription, histone modifications, and splicing in Saccharomyces cerevisiae. Nucleic Acids Res *48*, 10241-10258. 10.1093/nar/gkaa745.

Separovich, R.J., Wong, M.W.M., Bartolec, T.K., Hamey, J.J., and Wilkins, M.R. (2022). Site-specific Phosphorylation of Histone H3K36 Methyltransferase Set2p and Demethylase Jhd1p is Required for Stress Responses in Saccharomyces cerevisiae. J Mol Biol *434*, 167500. 10.1016/j.jmb.2022.167500.

Smolle, M., Venkatesh, S., Gogol, M.M., Li, H., Zhang, Y., Florens, L., Washburn, M.P., and Workman, J.L. (2012). Chromatin remodelers Isw1 and Chd1 maintain chromatin structure during transcription by preventing histone exchange. Nat Struct Mol Biol *19*, 884-892. 10.1038/nsmb.2312.

Szatkowska, R., Garcia-Albornoz, M., Roszkowska, K., Holman, S.W., Furmanek, E., Hubbard, S.J., Beynon, R.J., and Adamczyk, M. (2019). Glycolytic flux in Saccharomyces cerevisiae is dependent on RNA polymerase III and its negative regulator Maf1. Biochem J *476*, 1053-1082. 10.1042/BCJ20180701.
