# Supplementary material for: Genetic effects on molecular network states explain complex traits
Source: Mol Syst Biol. 2023 Jul 24;19(8):e11493. doi: 10.15252/msb.202211493 (PMC10407735; doi:10.15252/msb.202211493)
Supplement: Supplementary file 2 — Expanded View Figures PDF [file MSB-19-e11493-s007.pdf]

Expanded View Figures

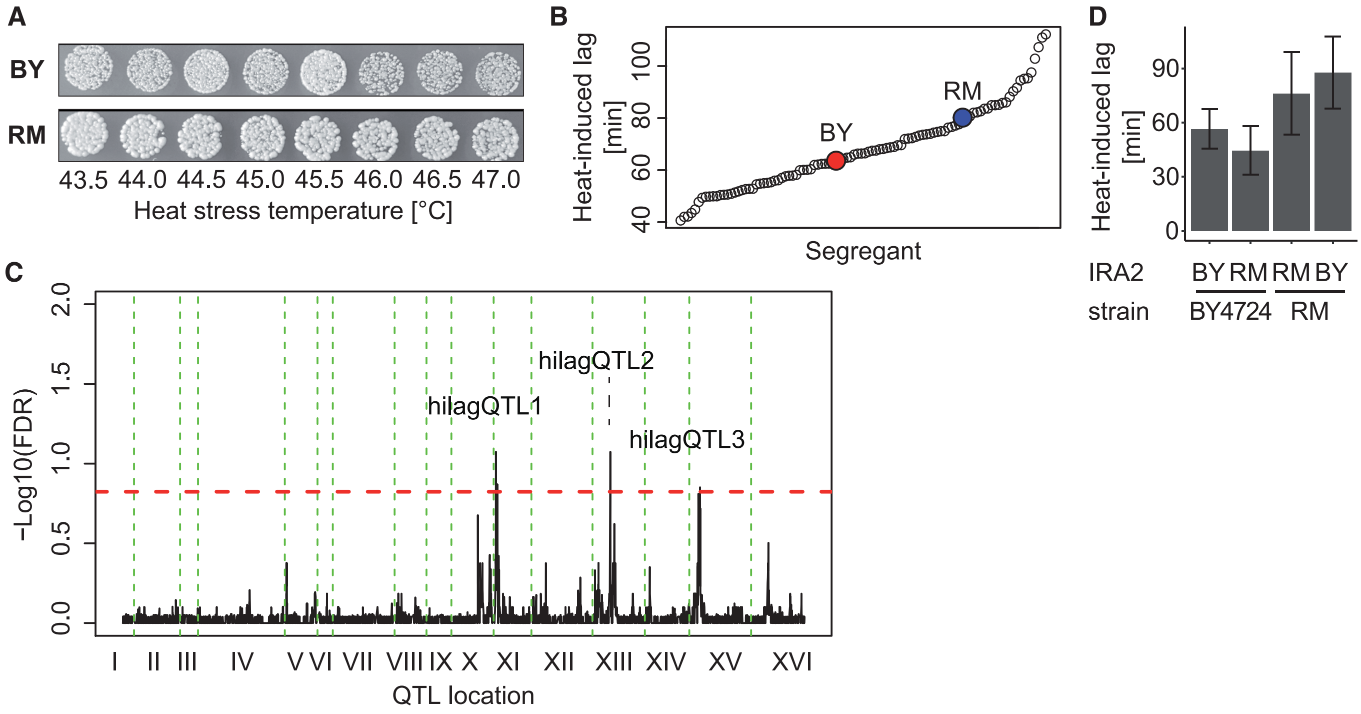

Figure EV1. Supporting experiments and QTL mapping result for heat-induced lag.

A Viability test for heat stress treatment. Samples from exponentially growing cultures of BY or RM were subjected to transient heat stress treatment (ramping from 25°C to indicated temperature at 1 K/s, followed by 8 min exposure at constant peak temperature) and spotted onto YPD plates following appropriate dilution. Spots were photographed after 1.5 days at 30°C.

B Distribution of heat-induced lag measurements across 100 segregants and the parental strains as indicated.

C QTL mapping result for heat-induced lag. Dashed red line represents 15% FDR threshold based on comparison between mapping of true against permuted trait values. Loci that passed the threshold are indicated.

D Heat-induced lag measurements (n = 4 biological replicates) in BY4724, which is closely related to the parental strain BY4716, and in RM11-1a as well as in derivatives after allele-swapping of *IRA2* (Smith & Kruglyak, 2008). Error bars indicate mean ± SD.

 © 2023 The Authors

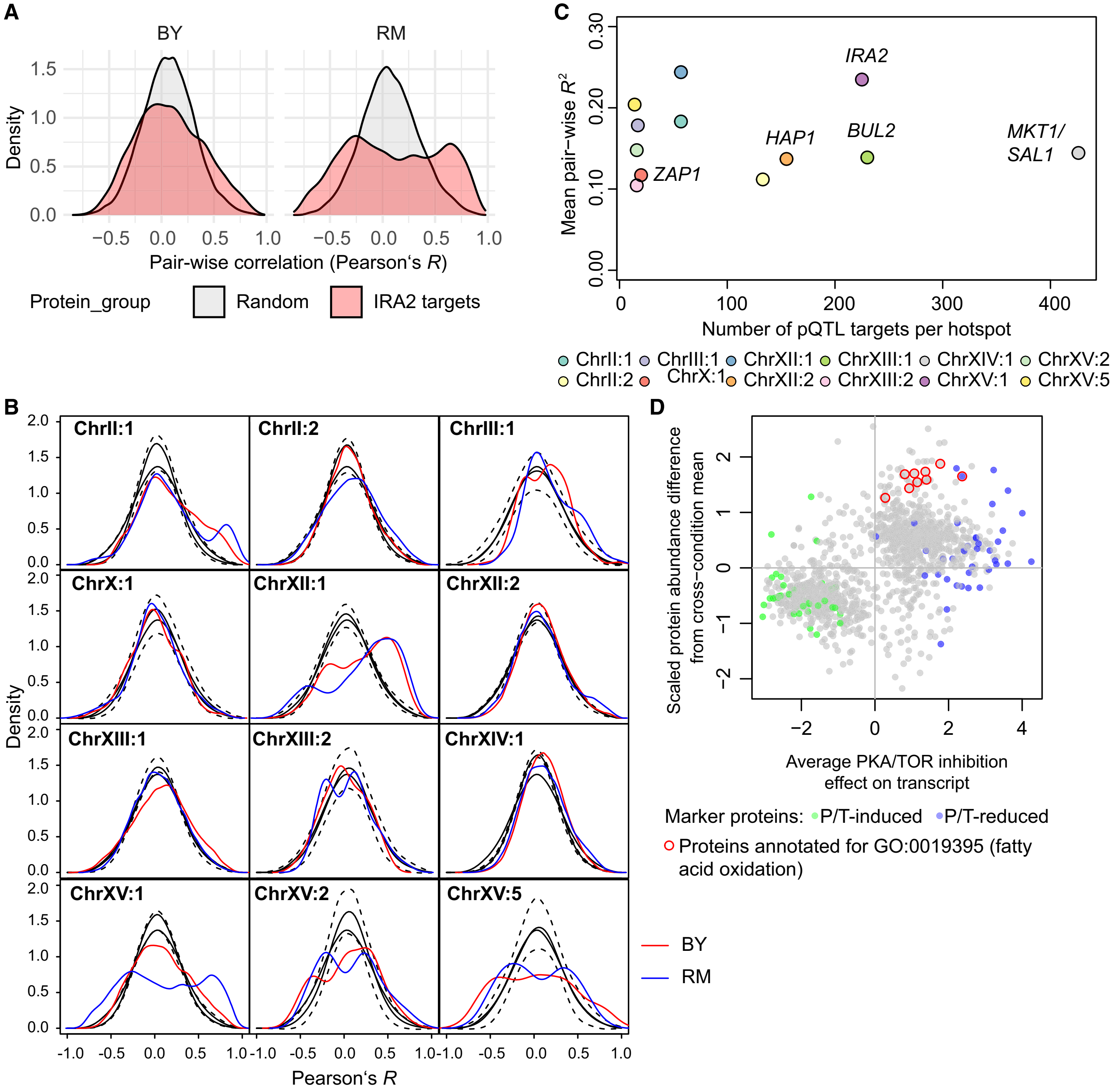

A
BY
RM
Density
Pair-wise correlation (Pearson's R)
Protein_group
Random
IRA2 targets
C
IRA2
HAP1
BUL2
MKT1/ SAL1
ZAP1
Mean pair−wise R^2
Number of pQTL targets per hotspot
ChrII:1 ChrIII:1 ChrXII:1 ChrXIII:1 ChrXIV:1 ChrXV:2
ChrII:2 ChrX:1 ChrXII:2 ChrXIII:2 ChrXV:1 ChrXV:5
B
ChrII:1
ChrII:2
ChrIII:1
ChrX:1
ChrXII:1
ChrXII:2
ChrXIII:1
ChrXIII:2
ChrXIV:1
ChrXV:1
ChrXV:2
ChrXV:5
Density
Pearson's R
BY
RM
D
Scaled protein abundance difference from cross−condition mean
Average PKA/TOR inhibition effect on transcript
Marker proteins: P/T-induced P/T-reduced
Proteins annotated for GO:0019395 (fatty acid oxidation)

Figure EV2.

Figure EV2. Coordinated expression among targets of pQTL hotspots and illustration of proteomic state of samples grown on oleate as carbon source.

A Test for coordinated expression among protein abundance targets of the *IRA2* pQTL hotspot. Pair-wise correlation among 225 proteins shown separately for sets of strains carrying either the BY or the RM allele at the marker corresponding to the *IRA2* gene. The gray-colored area represents a representative example of the distribution of pair-wise correlations in abundance-matched but otherwise random sets of proteins. The average distribution as well as standard deviation of the frequency distribution (200 bins) are shown in (B).

B Same test for coordination as in (A) but for each of 12 pQTL hotspots as described in (Grossbach *et al*, 2022). Pair-wise correlations between protein abundance targets of the indicated hotspot were calculated in sets of strains split by the corresponding most significant marker (BY: red curve, RM: blue curve). Black solid lines show the average distribution of pair-wise correlations in random sets of proteins that were matched in abundance to the targets of the respective hotspot. These pair-wise correlations were calculated in the same sets of strains as used for the actual targets, represented by two different solid lines. Dashed lines show the maximum and minimum among 1,000 random samples of proteins, calculated for 200 bins across the range from −1 to +1.

C Comparison of coordinated expression of pQTL hotspot targets (average of all pair-wise correlations as shown in (B)) and the number of targets for each hotspot.

D Comparison between protein abundances differences (scaled and centered across 10 carbon sources) for the sample grown on oleate from (Paulo *et al*, 2016) to the average effect of PKA and TOR inhibition (20 min, Kunkel *et al*, 2019) on the corresponding transcript. PKA (P) and TOR (T)-induced and TOR (T)-reduced marker proteins are highlighted in green and blue, respectively.

Figure EV3. Proteome-wide comparisons of pQTL effects to the effects of chemical inhibition and functional delineation of effects at pQTL hotspot ChrXIV:1.

A Comparison of protein abundance changes associated with allele differences at 12 pQTL hotspots to the effect of chemical inhibition of PKA and TOR signaling pathways on transcript abundance (Kunkel *et al*, 2019). The effect of chemical inhibition as shown here represents the average effect following 20 min inhibition of either pathway. Marker genes used for calculation of the PT score are highlighted.

B Same comparison as in panel A for genetic loci with strong PT score mapping signals and without accumulation of pQTL (see Fig 4A and main text).

C Comparison of protein abundance changes due to allelic differences at hotspot ChrXIV:1, which spans the *MKT1* and *SAL1* loci, to global correlation of respective protein abundances with the PT score across BYxRM segregants. The x-axis shows the beta coefficient of the PT score as a predictor of protein abundance in a combined linear model with the genotype at the hotspot ChrXIV:1 locus to correct for the effect of the locus itself. Analysis by partitioning of proteins into GO terms as indicated.

D Same analysis as in panel C but coloring according to consistent (green) or inconsistent (red) direction of protein abundance change with allelic difference at the ChrXIV:1 locus compared to the expected direction based on the PT score difference at this locus.

 © 2023 The Authors

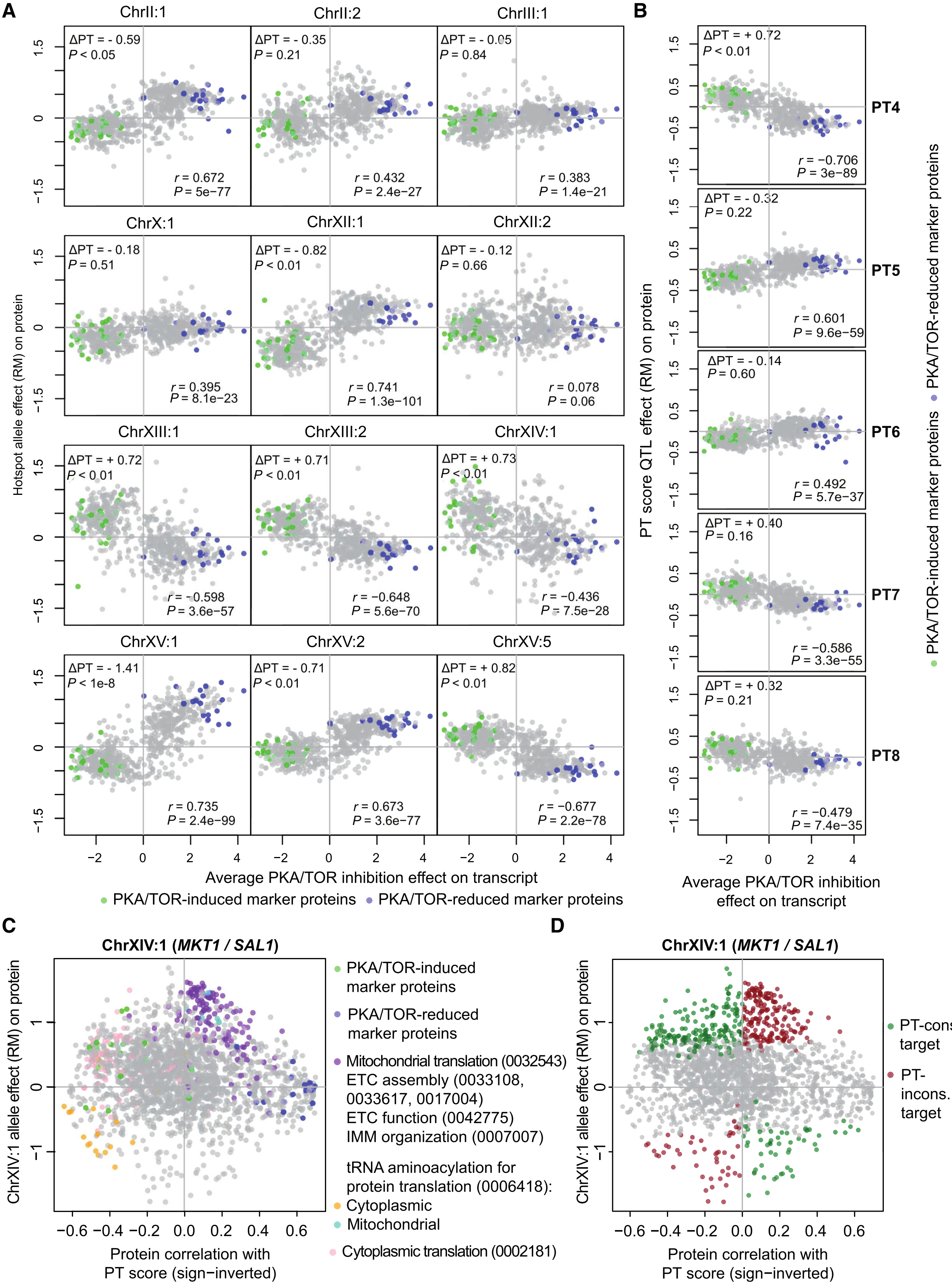
A
ChrII:1
ΔPT = - 0.59
P < 0.05
r = 0.672
P = 5e−77
ChrII:2
ΔPT = - 0.35
P = 0.21
r = 0.432
P = 2.4e−27
ChrIII:1
ΔPT = - 0.05
P = 0.84
r = 0.383
P = 1.4e−21
ChrX:1
ΔPT = - 0.18
P = 0.51
r = 0.395
P = 8.1e−23
ChrXII:1
ΔPT = - 0.82
P < 0.01
r = 0.741
P = 1.3e−101
ChrXII:2
ΔPT = - 0.12
P = 0.66
r = 0.078
P = 0.06
ChrXIII:1
ΔPT = + 0.72
P < 0.01
r = −0.598
P = 3.6e−57
ChrXIII:2
ΔPT = + 0.71
P < 0.01
r = −0.648
P = 5.6e−70
ChrXIV:1
ΔPT = + 0.73
P < 0.01
r = −0.436
P = 7.5e−28
ChrXV:1
ΔPT = - 1.41
P < 1e-8
r = 0.735
P = 2.4e−99
ChrXV:2
ΔPT = - 0.71
P < 0.01
r = 0.673
P = 3.6e−77
ChrXV:5
ΔPT = + 0.82
P < 0.01
r = −0.677
P = 2.2e−78
Hotspot allele effect (RM) on protein
Average PKA/TOR inhibition effect on transcript
PKA/TOR-induced marker proteins
PKA/TOR-reduced marker proteins
B
ΔPT = + 0.72
P < 0.01
r = −0.706
P = 3e−89
PT4
ΔPT = - 0.32
P = 0.22
r = 0.601
P = 9.6e−59
PT5
ΔPT = - 0.14
P = 0.60
r = 0.492
P = 5.7e−37
PT6
ΔPT = + 0.40
P = 0.16
r = −0.586
P = 3.3e−55
PT7
ΔPT = + 0.32
P = 0.21
r = −0.479
P = 7.4e−35
PT8
PT score QTL effect (RM) on protein
Average PKA/TOR inhibition effect on transcript
PKA/TOR-induced marker proteins
PKA/TOR-reduced marker proteins
C
ChrXIV:1 (MKT1 / SAL1)
ChrXIV:1 allele effect (RM) on protein
Protein correlation with PT score (sign−inverted)
PKA/TOR-induced marker proteins
PKA/TOR-reduced marker proteins
Mitochondrial translation (0032543)
ETC assembly (0033108, 0033617, 0017004)
ETC function (0042775)
IMM organization (0007007)
tRNA aminoacylation for protein translation (0006418):
Cytoplasmic
Mitochondrial
Cytoplasmic translation (0002181)
D
ChrXIV:1 (MKT1 / SAL1)
ChrXIV:1 allele effect (RM) on protein
Protein correlation with PT score (sign−inverted)
PT-cons. target
PT-incons. target

Figure EV3.

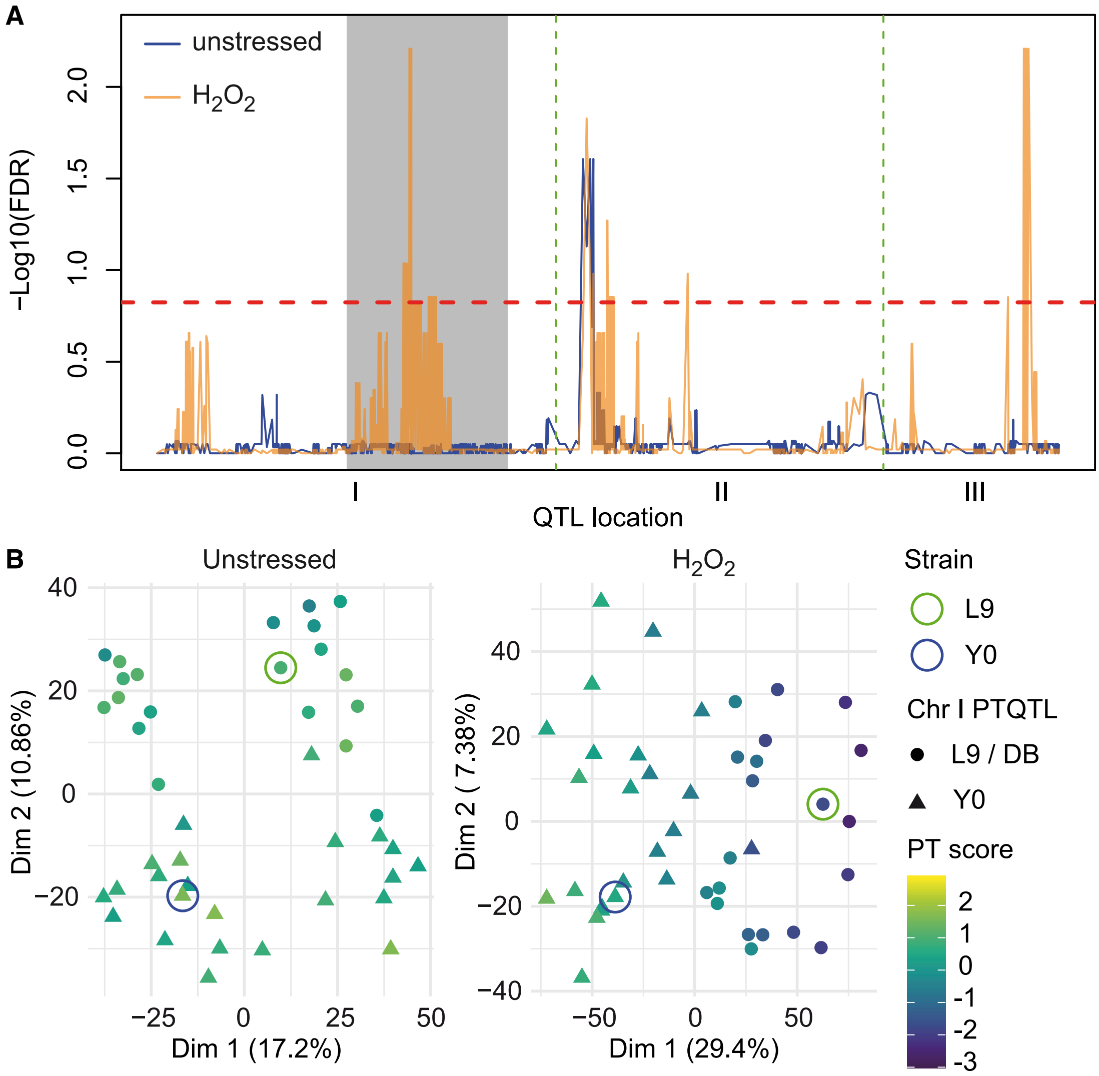

Figure EV4. Supporting results for PT score-based analysis of the fission yeast three-way cross.

A QTL mapping of the PT score in the fission yeast 3-way cross in two conditions, as indicated. 15% FDR indicated by red dashed horizontal line. Shaded area represents inverted region of Chromosome I in the Y0 parental strain.

B PCA based on transcriptome variability for 43 segregants between the L9 and Y0 parental strains (cross R1, parental strains highlighted) in two conditions. Strains are colored by PT score and shape represents allele identity at the most significant marker of PTQTL1 for strains in H_2O_2.

 © 2023 The Authors
