# Supplementary material for: Genetic effects on molecular network states explain complex traits
Source: Mol Syst Biol. 2023 Jul 24;19(8):e11493. doi: 10.15252/msb.202211493 (PMC10407735; doi:10.15252/msb.202211493)
Supplement: Supplementary file 9 [file MSB-19-e11493-s006.pdf]

molecular systems biology

Genetic effects on molecular network states explain complex traits

Matthias Weith, Jan Grossbach, Mathieu Clément-Ziza3, Ludovic Gillet, Maria Rodriguez-Lopez, Samuel Marguerat, Christopher Workman, Paola Picotti, Jürg Bähler, Aebersold Rudolf, and Andreas Beyer
DOI: 10.15252/msb.202211493

Corresponding author(s): Andreas Beyer (andreas.beyer@uni-koeln.de)

Review Timeline:

Submission Date:	2nd Dec 22
Editorial Decision:	30th Jan 23
Revision Received:	8th May 23
Editorial Decision:	30th May 23
Revision Received:	29th Jun 23
Accepted:	4th Jul 23

Editors: Jingyi Hou and Maria Polychronidou

Transaction Report:

(Note: With the exception of the correction of typographical or spelling errors that could be a source of ambiguity, letters and reports are not edited. Depending on transfer agreements, referee reports obtained elsewhere may or may not be included in this compilation. Referee reports are anonymous unless the Referee chooses to sign their reports.)

30th Jan 2023

Manuscript Number: MSB-2022-11493
Title: Large-scale changes of molecular network states explain complex traits
Author: Andreas Beyer
Matthias Weith
Jan Grossbach
Mathieu Clément-Ziza3
Ludovic Gillet
Maria Rodriguez-Lopez
Paola Picotti
Jürg Bähler
Aebersold Rudolf
Samuel Marguerat
Christopher Workman

Dear Prof Beyer,

Thank you for submitting your work to Molecular Systems Biology. I apologize for the delay in sending you an editorial decision, which was due to the late arrival of the reviewers' reports. We have now heard back from the three reviewers who agreed to evaluate your manuscript. As you will see from the reports below, the reviewers acknowledge the potential interest of the study. They raise however a series of concerns, which we would ask you to address in a major revision.

I think the recommendations of the reviewers are rather clear, so there is no need to repeat the points listed below. All the issues raised by the reviewers need to be satisfactorily addressed. As you may already know, our editorial policy allows in principle a single round of major revision, so it is essential to provide responses to the reviewers' comments that are as complete as possible. Please feel free to contact me in case you would like to discuss in further detail any of the issues raised by the reviewers.

On a more editorial level, we would ask you to address the following issues:

- Please provide a .docx formatted version of the manuscript text (including legends for main figures, EV figures and tables). Please make sure that the changes are highlighted to be clearly visible.

- Please provide individual production quality figure files as .eps, .tif, .jpg (one file per figure).

-Please provide a .docx formatted letter INCLUDING the reviewers' reports and your detailed point-by-point responses to their comments. As part of the EMBO Press transparent editorial process, the point-by-point response is part of the Review Process File (RPF), which will be published alongside your paper.

-Please note that all corresponding authors are required to supply an ORCID ID for their name upon submission of a revised manuscript.

-We replaced Supplementary Information with Expanded View (EV) Figures and Tables that are collapsible/expandable online (see examples in http://msb.embopress.org/content/11/6/812). A maximum of 5 EV Figures can be typeset. EV Figures should be cited as 'Figure EV1, Figure EV2" etc... in the text and their respective legends should be included in the main text after the legends of regular figures.

Additional Tables/Datasets should be labeled and referred to as Table EV1, Dataset EV1, etc. Legends have to be provided in a separate tab in case of .xls files. Alternatively, the legend can be supplied as a separate text file (README) and zipped together with the Table/Dataset file.

For the figures and tables that you do NOT wish to display as Expanded View figures, they should be bundled together with their legends in a single PDF file called *Appendix*, which should start with a short Table of Content. Each legend should be below the corresponding Figure/Table in the Appendix. Appendix figures and tables should be referred to in the main text as: "Appendix Figure S1, Appendix Figure S2, Appendix Table S1" etc. See detailed instructions regarding expanded view here: https://www.embopress.org/page/journal/17444292/authorguide#expandedview.

-Before submitting your revision, primary datasets (and computer code, where appropriate) produced in this study need to be deposited in an appropriate public database (seehttp://msb.embopress.org/authorguide - dataavailability https://www.embopress.org/page/journal/17444292/authorguide#dataavailability).

Please remember to provide a reviewer password if the datasets are not yet public.

The accession numbers and database should be listed in a formal "Data Availability" section (placed after Materials & Method) that follows the model below (see also https://www.embopress.org/page/journal/17444292/authorguide#dataavailability). Please note that the Data Availability Section is restricted to new primary data that are part of this study.

Data availability

The datasets (and computer code) produced in this study are available in the following databases:

- RNA-Seq data: Gene Expression Omnibus GSE46843 (https://www.ncbi.nlm.nih.gov/geo/query/acc.cgi?acc=GSE46843)
- [data type]: [name of the resource] [accession number/identifier/doi] ([URL or identifiers.org/DATABASE:ACCESSION])

*** Note - All links should resolve to a page where the data can be accessed. ***

-At EMBO Press we ask authors to provide source data for the main figures. Our source data coordinator will contact you to discuss which figure panels we would need source data for and will also provide you with helpful tips on how to upload and organize the files.

- Our journal encourages inclusion of *data citations in the reference list* to directly cite datasets that were re-used and obtained from public databases. Data citations in the article text are distinct from normal bibliographical citations and should directly link to the database records from which the data can be accessed. In the main text, data citations are formatted as follows: "Data ref: Smith et al, 2001". In the Reference list, data citations must be labeled with "[DATASET]". A data reference must provide the database name, accession number/identifiers and a resolvable link to the landing page from which the data can be accessed at the end of the reference. Further instructions are available at .

- We updated our journal's competing interests policy in January 2022 and request authors to consider both actual and perceived competing interests. Please review the policy https://www.embopress.org/competing-interests and update your competing interests if necessary.

Please use the heading "Disclosure statement and competing interests".

- All Materials and Methods need to be described in the main text. We would encourage you to use 'Structured Methods', our new Materials and Methods format. According to this format, the Material and Methods section should include a Reagents and Tools Table (listing key reagents, experimental models, software and relevant equipment and including their sources and relevant identifiers) followed by a Methods and Protocols section in which we encourage the authors to describe their methods using a step-by-step protocol format with bullet points, to facilitate the adoption of the methodologies across labs. More information on how to adhere to this format as well as downloadable templates (.doc or .xls) for the Reagents and Tools Table can be found in our author guidelines: < https://www.embopress.org/page/journal/17444292/authorguide#researcharticleguide>. An example of a Method paper with Structured Methods can be found here: .

-Regarding data quantification:
Please ensure to specify the name of the statistical test used to generate error bars and P values, the number (n) of independent experiments (please specify technical or biological replicates) underlying each data point and the test used to calculate p-values in each figure legend. Discussion of statistical methodology can be reported in the materials and methods section, but figure legends should contain a basic description of n, P and the test applied.
Graphs must include a description of the bars and the error bars (s.d., s.e.m.).
Please also include scale bars in all microscopy images.

- Please provide a "standfirst text" summarizing the study in one or two sentences (approximately 250 characters, including space), three to four "bullet points" highlighting the main findings and a "synopsis image" (550px width and 400-600 px height, PNG format) to highlight the paper on our homepage.
Here are a couple of examples:
https://www.embopress.org/doi/10.15252/msb.20199356
https://www.embopress.org/doi/10.15252/msb.20209475
https://www.embopress.org/doi/10.15252/msb.209495

When you resubmit your manuscript, please download our CHECKLIST (https://www.embopress.org/pb-assets/embo-site/EMBO%20Press%20Author%20Checklist-1642513524327.xlsx) and include the completed form in your submission. *Please note* that the Author Checklist will be published alongside the paper as part of the transparent process (https://www.embopress.org/page/journal/17444292/authorguide#transparentprocess).

If you feel you can satisfactorily deal with these points and those listed by the referees, you may wish to submit a revised version

of your manuscript. Please attach a covering letter giving details of the way in which you have handled each of the points raised by the referees. A revised manuscript will be once again subject to review and you probably understand that we can give you no guarantee at this stage that the eventual outcome will be favorable.

I look forward to receiving your revised manuscript soon.

Kind regards,
Jingyi

Jingyi Hou, PhD
Scientific Editor
Molecular Systems Biology

We realize that it is difficult to revise to a specific deadline. In the interest of protecting the conceptual advance provided by the work, we recommend a revision within 3 months (30th Apr 2023). Please discuss the revision progress ahead of this time with the editor if you require more time to complete the revisions. Use the link below to submit your revision:

https://msb.msubmit.net/cgi-bin/main.plex

IMPORTANT: When you send your revision, we will require the following items:
1. the manuscript text in LaTeX, RTF or MS Word format
2. a letter with a detailed description of the changes made in response to the referees. Please specify clearly the exact places in the text (pages and paragraphs) where each change has been made in response to each specific comment given
3. three to four 'bullet points' highlighting the main findings of your study
4. a short 'blurb' text summarizing in two sentences the study (max. 250 characters)
5. a 'thumbnail image' (550px width and max 400px height, Illustrator, PowerPoint or jpeg format), which can be used as 'visual title' for the synopsis section of your paper.
6. Please include an author contributions statement after the Acknowledgements section (see https://www.embopress.org/page/journal/17444292/authorguide)
7. Please complete the CHECKLIST available at (https://bit.ly/EMBOPressAuthorChecklist).
Please note that the Author Checklist will be published alongside the paper as part of the transparent process (https://www.embopress.org/page/journal/17444292/authorguide#transparentprocess).
8. When assembling figures, please refer to our figure preparation guideline in order to ensure proper formatting and readability in print as well as on screen:
https://bit.ly/EMBOPressFigurePreparationGuideline
See also figure legend guidelines: https://www.embopress.org/page/journal/17444292/authorguide#figureformat
9. Please note that corresponding authors are required to supply an ORCID ID for their name upon submission of a revised manuscript (EMBO Press signed a joint statement to encourage ORCID adoption).
(https://www.embopress.org/page/journal/17444292/authorguide#editorialprocess)
Currently, our records indicate that the ORCID for your account is 0000-0002-3891-2123.

Please click the link below to modify this ORCID:
Link Not Available

10. At EMBO Press we ask authors to provide source data for the main manuscript figures. Our source data coordinator will contact you to discuss which figure panels we would need source data for and will also provide you with helpful tips on how to upload and organize the files.

The system will prompt you to fill in your funding and payment information. This will allow Wiley to send you a quote for the article processing charge (APC) in case of acceptance. This quote takes into account any reduction or fee waivers that you may be eligible for. Authors do not need to pay any fees before their manuscript is accepted and transferred to the publisher.

EMBO Press participates in many Publish and Read agreements that allow authors to publish Open Access with reduced/no publication charges. Check your eligibility: https://authorservices.wiley.com/author-resources/Journal-Authors/open-access/affiliation-policies-payments/index.html

As a matter of course, please make sure that you have correctly followed the instructions for authors as given on the submission website.

*** PLEASE NOTE *** As part of the EMBO Press transparent editorial process initiative (see our Editorial at https://dx.doi.org/10.1038/msb.2010.72), Molecular Systems Biology publishes online a Review Process File with each accepted manuscripts. This file will be published in conjunction with your paper and will include the anonymous referee reports, your point-by-point response and all pertinent correspondence relating to the manuscript. If you do NOT want this File to be published, please inform the editorial office at msb@embo.org within 14 days upon receipt of the present letter.

Reviewer #1:

In this manuscript, the authors work to investigate the architecture of QTLs influencing a yeast population. They identify a common link to the PKA/TOR pathways and work to link the multi-omic analysis to a single model built on PKA/TOR status in the genotypes arguing that the QTLs are shifting a common cellular state. The introduction provides an image that this is a completely new concept but at some levels, this is very similar to plant multiomic analysis of QTLs and network QTL work (E.g. doi:10.1186/gb-2008-9-8-r129 and 10.1105/tpc.110.082065) indicating different levels of pleiotropy across major QTLs that may partly be shaped by network architecture and there can be a common mechanistic influence. The focus on largely human and a few yeast citations doesn't provide the necessary information to place this work in a broader context nor allow the work to clearly delineate what it may add to our understanding of quantitative genetics.
•
I'm partly struggling with the discussion of parsing loci into global, local and regional. While the mathematical analysis is fairly clear, there is one question that I'm not fully understanding. This may be because they are out of the scope of the paper but some discussion is warranted. What is the clear evidence that it is the mechanistic neighborhood of a causal variant that creates the difference in global/local/regional rather than variation in immediate effect sizes that may cause this difference. For example, a tiny effect variant in TOR may actually appear local if the variation of this effect propagates out such that there is increasing noise with each increasing step in causality. Is there any information that may pertain to this in the yeast mechanistic studies to help support the statistical arguments? I worry that simply building on the statistics to make these claims could be circular and global, local, regional may not be a true network mechanistic reflection.

While cloning and validating every QTL would help to answer this question, that is easily outside of the realm of this work. Maybe analyzing the mechanistic multi-locus epistasis work by the Ehrenrich lab in yeast with this approach could help to support a mechanistic basis. Or alternatively a more cautious discussion on statistical to mechanistic inference.

Part of the above worry is actually illustrated in the graphical abstract. In that abstract, the nutrients are illustrated at upstream of PKA/TOR while downstream is metabolism. But in fact, the downstream metabolism is the self-same upstream nutrients and as such, this is not a linear system but is in fact a circular system. Thus, the idea that local effects are purely downstream doesn't truly fit to the reality.

Line 32 - Is the omnigenic model really in opposition to the polygenic model? From what I have read, the omingenic is an attempt to explain the effect size distributions from the poly/oligogenic observations. Not really a this-or-that concept as indicated by the use of opposition.

Line 61, I'm unsure if the equation of network state constraint to gene purifying selection is fully warranted. There are examples of network states, e.g. plant primary metabolism, where the networks appear constrained in natural accessions but upon crossing accessions, the progeny showed extensive variation. This indicated that the phenotypic constraint is not created by genetic constraint underlying the network state. It is possible to have constraint on the networks phenotypes while allowing the underlying genes to be less constrained.

Line 272, is there a biological reason that oleate may be an outlier? It appears from the figure that metabolites associated with B-oxidation (Pyruate, acetate and oleate) may be creating a different relationship between PT and PC1. For example, is TOR less responsive to variation in TCA/B-oxidation carbon/energy than it is to the sugar related carbon/energy from the other metabolties in Figure 2E. As such, the exclusion of oleate maybe helping to drive the impression of a singular relationship between PT and PC1. It would helpful to be more cautious in the interpretation of the capacity of this singular model to explain the variation in this cross.

Line 586-597, is the S. pombe comparison showing the broader utility of this approach or is it showing that Yeast have a similar niche and thereby similar architecture on TOR. Or is it showing that domestication of yeasts follows similar avenues? The application to another species is very interesting but I'm not sure that the use of S. pombe allows for the breadth of the introduction and the discussion. It is possible that the architecture of the idea is more generally applicable than the focus on TOR. TOR may have something to do with Yeast ecology that does not apply across other species. For example, in line 649, one could argue that yeasts are not a large evolutionary distance.

Reviewer #2:

In this paper, Weith et al. address an important question in complex trait genetics: which genetic perturbations lead to widespread changes in the cellular network versus those that are confined to a small subset of cellular functions? To do so, they introduce the concept of a single "PT" score meant to capture the activity of the PKA and TOR pathways and that is calculated as a weighted average of protein levels (or, later on, transcript levels) of key genes related to these pathways.
To arrive at the score, the authors begin by QTL-mapping the growth response of a yeast segregant panel to a sublethal heat stress and relate variation in heat recovery to proteome data the authors collected earlier. Using a series of analyses in numerous other yeast datasets, the authors show that the PT score captures much of the variation in proteomes in a variety of contexts, suggesting that the score is a useful measure of a critical axis (fermentation vs. respiration) along which cell state varies. The authors go on to describe how genetic variants impact the PT score by either modulating one or few genes, a single module, or variation across the entire cell network. The paper concludes by showing that the PT score is also a useful concept in yeast species other than cerevisiae.

Overall, I enjoyed this paper. It presents a comprehensive and detailed exploration of the authors' ideas and lays out a useful framework for thinking about complex traits. The text is easy to follow, and the figures are clear and attractive. The paper is a little unusual in how it begins with a relatively small QTL mapping experiment, spends most of its text on computational reanalyses of existing data, and then crams a fairly large amount of experimental work (a three-cross, two-condition eQTL mapping experiment in fission yeast) into a single section (out of 9 sections). However, this unusual structure ends up working well in weaving together the different elements of the paper.

I only have a few minor comments:

1. The paper is quite long, and I suggest the authors find ways to shorten the main text. It probably isn't necessary to describe each of the many results in as much detail as currently presented. For example, there are a lot of results on the PT score, where exactly it maps, which proteomes it correlates with and how, etc. This could probably be streamlined - after all, it is not surprising that a score derived from protein abundances will, for example, map where the protein abundances map (Figure 2F / section 4), and correlate with protein abundances in other datasets. Some detail in this and other sections can probably be moved to Supplement.

2. Line 272: the callout to Figure 2E does not seem to be correct, or rather, the panel labels in Figure 2 may not be correct. In the figure, "E" seems to label the phosphoproteomics results rather than the PC1/PK score panel, which seems to have no dedicated label. Rather than using "2D lower panel", I'd just use more lettered panels.

3. Line 281: Figure 2F does not show PT score correlations with "the first dimension in a PCA of the BYxRM proteomes" - it shows QTL traces. Mislabeled panel?

4. Line 319: callout to 2G should be to 2F?

5. Line 330: there is no panel 2H

6. Did panel 2E (the actual 2E, showing phosphoproteome results) ever get mentioned in the text?

7. L. 361: it is not clear to me across which data these correlations were computed. A given gene is either assigned to a given GO term or not - where does abundance variation come into play here?

8. L. 400: I don't understand the use of "adaption" of cellular processes here. Adaptation in what sense? Presumably not evolutionarily? Do the authors just mean "change", or "shifts"? The same comment applies to line 20 in the graphical abstract and line 414. There probably are more. Please change or explain what is meant here.

Reviewer #3:

In this study, Weith et al. perform a series of physiological and multi-omic analyses in a mapping population of yeast strains (the BYxRM cross). They conclude that a "network state" corresponding to PKA/TOR signaling activity (the PT state) is the dominant axis of variation driving multiple physiological processes, including heat-induced lag and environmental changes in carbon sources. They summarize the PT state using a one-dimensional PT score, which they then genetically map. Much of the results section is devoted to motivating, defining, and characterizing the PT state. Their essential conceptual assertion is that genetic variants in this cross effectively cluster into three groups, which they call local, regional, and global. Global genetic effects are those that have a large influence on the PT score, which encodes complex constraints on the molecular physiology. Regional and local effects, by contrast, have detectable effects as protein abundance quantitative trait loci (pQTLs), but have only small effects on the PT score. In support of this claim, they perform a bioinformatic network analysis quantifying the network distances

of the targets of pQTL hotspots. They show that pQTL hotspots that colocalize with QTLs for PT state (i.e. global effects) have more distant target genes than hotspots that do not correspond to PT state QTLs (i.e., local and regional effects). They interpret this result to mean that global effects that integrate up to cellular physiology must necessarily target a set of functionally distant genes.

Overall, I found the presentation challenging and had to dig through too many details to understand their essential point. In particular, there is an inconsistency in the writing between advocating for a novel conceptual model for genetic effects (local, regional, and global) vs. arguing for the importance of PT network state per se. The genetic analysis of low-dimensional representations of multi-omic data is not new, and their data show that PT state strongly correlates with principal components (PCs) of several omics signatures. Thus, the paper could be substantially streamlined by removing the detailed description and characterization of the PT state, which, for all intents and purposes, functions as a marker for the first PC across different conditions. If, on the other hand, the main assertion is instead that the PT state is an essential single dimension defining many responses of yeast to perturbations, then this should be stated clearly and does not appear to need a new conceptual model for the genetic architecture of complex traits.

I found the network analysis (Fig. 4) conceptually elegant, but I have concerns with their approach. Could they simply plot the average network distance between pQTL targets and the effect size of the locus on PT score? This seems the most straightforward way to establish the validity of their model. More generally, I am concerned that there is a potential circularity in their data that is not discussed. It is a tautology that if a locus has a large influence on PT score, then it will necessarily have many pQTLs. Equivalently, if a locus does not have many pQTLs, it cannot have a large influence on PT score. They show that the network spread of the hotspot targets does not correlate with the number of targets, but the important question is whether, after controlling for the number of targets, the spread of a hotspot significantly predicts the effect of the locus on PT score. This would show specifically that it is the targeting of functionally distant genes that is critical for altering the PT "network state".

Finally, I would like to see a discussion of how they propose we use their proposed model for genetic effects. Is it predictive, in that we can know a priori local, from regional, from global effects just from, say, proteomic data? (A significant correlation between network spread and effect size for PT state would argue yes.) Should this model alter how we design experiments or analyses for complex traits? If so, how? Without such discussion, we are left wondering what the authors make of these observations.

Manuscript MSB-2022-11493

Dear Maria, dear Jingyi,

thank you very much for submitting our manuscript “Large-scale changes of molecular network states explain complex traits” to review and for inviting a revision!

We very much thank the three reviewers for their critical and constructive comments, which have greatly helped us to improve our manuscript. In order to address their concerns we have substantially revised the text to improve the presentation of our material, including the reorganization of the order in which we present the results. We have streamlined the flow to make the text more accessible and we have added new analyses. In particular, two of the reviewers had concerns regarding the network analysis. To clearly demonstrate that certain genetic effects cause global network re-organization, we have substantially expanded the analysis of ‘spreading’ effects in the network now including all genomic loci. Our new results confirm the notion that the ‘spreading’ of effects in molecular networks is not simply a function of effect size (stronger effects affecting more genes), but also depends on the specific mechanism of a polymorphism. To better represent the results of this revised analysis, we adapted the title of the manuscript and now refer to “genetic changes” instead of “large-scale changes”. Further, we have clarified that we do not assume that global effects necessarily operate via changing the PT network state. Instead, global network effects may also emerge through other central signaling pathways. This is certainly true in metazoan species with a substantially more complex organization of molecular network states due to the need to create a greater diversity of cell types.

Please, find below a point-by-point response to all of the reviewer comments. We hope that you can now accept our manuscript for publication and we are looking forward to the feedback from you and the reviewers.

Sincerely yours,
Andreas Beyer

Reviewer #1:

> *In this manuscript, the authors work to investigate the architecture of QTLs influencing a yeast population. They identify a common link to the PKA/TOR pathways and work to link the multi-omic analysis to a single model built on PKA/TOR status in the genotypes arguing that the QTLs are shifting a common cellular state.*
>
> *The introduction provides an image that this is a completely new concept but at some levels, this is very similar to plant multiomic analysis of QTLs and network QTL work (E.g. doi:10.1186/gb-2008-9-8-r129 and 10.1105/tpc.110.082065) indicating different levels of pleiotropy across major QTLs that may partly be shaped by network architecture and there can be a common mechanistic influence.*

We thank Reviewer #1 for pointing out these interesting and related studies. Our analysis seeks to understand the effects of genetic variants that shift the balance between various functional modules (as depicted in Figure 3). We present evidence that this shift results in a global reorganization of the molecular network. Indeed, both studies mentioned by Reviewer #1 report related observations:
In the study conducted by Vreugdenhil and co-workers (Keurentjes, 2008), variation in a gene with important functions during *Arabidopsis thaliana* development (ERECTA) is found to underlie large parts of the variation in primary carbon metabolism in a cross between the L*er* and Cvi accessions. Further loci might have shifted individuals along the same "axis" but were not detectable. While the study does not provide transcriptome-wide data to investigate this in more detail, a process linked to ERECTA could indeed constitute a similar network state-controlling mechanism as the one related to PKA/TOR-activity in yeast. Interestingly, these mechanisms share the property that related changes in central metabolic pathways occur both as a consequence of 'developmental progression' (allowing the diauxic shift in yeast to be understood as such) and of genetic variability.
Also of great interest for the concept of network states is the study conducted by Kliebenstein and co-workers (Kerwin, 2011). The authors developed a method to derive information about circadian clock configuration from single time-point microarray measurements. They subjected this "meta-trait" approximating circadian clock output to QTL mapping. As a result they found natural genetic variation in this trait that in part overlapped with metabolite QTL. Indeed, we believe that the PT network state is also strongly tied to the metabolic state of yeast and present an analysis of this state in Figure 5E.
These and other studies (our introduction highlights related studies in prokaryotes) show the existence of global network changes resulting from genetic variation. However, published work does not offer a conceptual framework that would explain why some genetic effects lead to large-scale network changes, while others do not. We would also like to point out that the concept of pleiotropy differs from the network state concept proposed here in that pleiotropy can have different causes and effects of which not all are mediated by network state regulation or via the "PT network state" in particular.
We now refer to the studies pointed out by Reviewer #1 in the introduction in the following way:

"Mechanisms that shift network states within the boundaries of viability have evolved to provide efficient modulation of cellular function. While environmental alterations may be a driving force to establish network state regulation in microorganisms (Balakrishnan et al., 2021), cells in adult multicellular organisms reside in relatively stable environments. Still, these cells undergo elaborate developmental paths during which cellular states have to be adjusted. Importantly, basic biochemical pathways such as the carbohydrate metabolism and developmental processes are functionally coupled and subject to genetic variation (ERECTA; (Keurentjes et al., 2008)). The circadian clock represents another network state-regulatory mechanism (Sun et al., 2021), which is also modulated through genetic variation (Jones et al., 2019; Kerwin et al., 2011)."

> *The focus on largely human and a few yeast citations doesn't provide the necessary information to place this work in a broader context nor allow the work to clearly delineate what it may add to our understanding of quantitative genetics.*

Reviewer #1 has pointed out two studies conducted in plants which indeed helped to place our work in a broader context. Genetic association studies conducted in humans are mainly referenced to illustrate the potential applications of the network state concept that we propose. We believe that the reduction of complexity afforded by the study of microorganisms helps us understand basic principles of the cellular molecular network. For example, the work of Terence Hwa is exceptional in its quantitative description of the allocation of an entire organismal proteome in response to environmental changes (You et al., 2013). We have re-written the discussion section to better delineate our work from earlier work, also in response to the last comment of Reviewer #3 (please, see below).

> *I'm partly struggling with the discussion of parsing loci into global, local and regional. While the mathematical analysis is fairly clear, there is one question that I'm not fully understanding. This may be because they are out of the scope of the paper but some discussion is warranted. What is the clear evidence that it is the mechanistic neighborhood of a causal variant that creates the difference in global/local/regional rather than variation in immediate effect sizes that may cause this difference. For example, a tiny effect variant in TOR may actually appear local if the variation of this effect propagates out such that there is increasing noise with each increasing step in causality. Is there any information that may pertain to this in the yeast mechanistic studies to help support the statistical arguments? I worry that simply building on the statistics to make these claims could be circular and global, local, regional may not be a true network mechanistic reflection. While cloning and validating every QTL would help to answer this question, that is easily outside of the realm of this work. Maybe analyzing the mechanistic multi-locus epistasis work by the Ehrenrich lab in yeast with this approach could help to support a mechanistic basis. Or alternatively a more cautious discussion on statistical to mechanistic inference.*

These are excellent points and similar concerns have been stated by Reviewer #3. Clearly, this point required additional analyses. Specifically, both reviewers were asking if the ‘spreading’ of genetic effects in networks may be dominated by the effect size (i.e. larger effects spreading further than smaller effects). We do agree that the ‘network spread’ (i.e. the range of a network affected by a variant) will scale with the effect size of a variant: larger effects will spread wider in the network. However, we also see variants with similar effect sizes (here measured as the number of target proteins) that have very different network spreads. Hence, the affected range of a network is not solely dependent on the effect size. Further, as the network spread increases with the effect size one would not necessarily expect that the <u>average</u> distance among those targets changes. We have substantially extended the network analysis (Figure 4) to address these points more thoroughly. Our interpretation of the results is that the average network distance between targets of a pQTL does not increase with the effect size. Rather, the average network distance for large numbers of proteins converges to slightly below the mean distance across the network (mean network distance: 3.188 edges; Fig. 4D). We further observed that targets of pQTL hotspots often displayed lower average network distances than random samples of the same size (i.e. below the network mean of 3.188). This is consistent with the notion that common targets of a hotspot should be functionally related and hence closer to each other in a network than a random sample of genes/proteins from the same network. The IRA2 hotspot is an exception from this trend, in that its targets display an exceptionally high average

network distance compared to random samples of the same size (Figure 4D). Hence, following our proposed classification, most strong genetic variant effects seem to be primarily ‘regional’. We further illustrate that large effect size is not a prerequisite for global network effects by analyzing five loci that were detectable at a relaxed threshold in our mapping of the PT score but were associated with low numbers of pQTL targets (Results, new section 4). The most affected proteins at these loci displayed higher average network distances than the targets of most pQTL hotspots. These results illustrate the concept of a global network effect and speak against variant effect size as the sole determinant of the spread of genetic effects.

> *Part of the above worry is actually illustrated in the graphical abstract. In that abstract, the nutrients are illustrated at upstream of PKA/TOR while downstream is metabolism. But in fact, the downstream metabolism is the self-same upstream nutrients and as such, this is not a linear system but is in fact a circular system. Thus, the idea that local effects are purely downstream doesn't truly fit to the reality.*

As described in the introduction, we assume that various mechanisms can affect network state regulation, including genetic variants that modulate the availability of specific intracellular nutrients that are being sensed as part of the network state-regulatory mechanism. Our model would attribute the effect of such variants on the network state to the ‘global’ type. We acknowledge that the graphical abstract suggested that local and global effects are in some kind of hierarchical relationship to each other, which they are not. We have changed the graphical abstract to avoid this source of misunderstanding.

> *Line 32 - Is the omnigenic model really in opposition to the polygenic model? From what I have read, the omingenic is an attempt to explain the effect size distributions from the poly/oligogenic observations. Not really a this-or-that concept as indicated by the use of opposition.*

In a perspective by Pritchard and colleagues, (Boyle, 2017) the “omnigenic model” of complex traits is delineated against the “polygenic model”. We agree with Reviewer #1 that the underlying observation may be in part identical with the distribution of effect sizes. However, the authors of the perspective propose that “all genes expressed in disease-relevant cells are liable to affect the functions of core disease-related genes” and contrast this with the concept that “one might expect disease-causing variants to cluster into key pathways that drive disease etiology”. It is this distinction that we refer to when setting polygenic and omnigenic models into opposition. Our proposed classification of genetic variant effects into local/regional/global modifies the polygenic model to include the possibility that peripheral genes can affect key pathways (or core genes) via an effect on a relevant network state and may in part explain the observations that led to the proposed omnigenic model. To mitigate the concern about the opposition between the two models, we have rephrased the corresponding sentence:

“Recent work has proposed an “omnigenic” <u>instead of</u> a “polygenic” model for the genetic architecture of complex traits in humans, [...]”

Line 61, I'm unsure if the equation of network state constraint to gene purifying selection is fully warranted. There are examples of network states, e.g. plant primary metabolism, where the networks appear constrained in natural accessions but upon crossing accessions, the progeny showed extensive variation. This indicated that the phenotypic constraint is not created by genetic constraint underlying the network state. It is possible to have constraint on the networks phenotypes while allowing the underlying genes to be less constrained.

Reviewer #1 is pointing out another interesting parallel between plant primary metabolism and the PT network state. The PT network state is also very similar between the two parental strains in the BYxRM cross but shows extensive variation in their progeny. We agree with the reviewer that genetic variation may not be counter-selected as long as the specific genotype maintains an optimal phenotype, which in our case corresponds to a specific network state. The two parental yeast strains BY and RM achieve an almost identical network state with two quite distinct genotypes. However, we would expect purifying selection to remove variants that would interfere with the network-state regulatory mechanism itself or which would strongly affect a module which requires balancing without allowing the regulatory mechanism to recover this balance. This is evidenced by the abundance of genetic effects with opposing network effects in this particular cross, i.e. the effect of one variant is balanced or ‘canceled out’ by one or more other variants.
To clarify the notion of fundamental constraints that shape the viable space, we have rephrased the corresponding paragraph in the introduction. We have removed the mention of purifying selection as it is not essential to understand the concept of a network state that we propose:

“Our model is based on the assumption that only a small fraction of all theoretically possible network states are physiologically feasible and favorable. The cellular network is globally constrained by basic biophysical principles, such as the need to balance anabolic and catabolic activities to preserve homeostasis and by cellular limits such as maximum proteome size, protein cost, molecular crowding or the availability of membrane space (Frei et al., 2020; Hu et al., 2020; Kleijn et al., 2022; Molenaar et al., 2009; Mori et al., 2021). Similar to requirements acting within functional modules, global constraints can result in coordinated changes across the entire network. Such network behavior has been demonstrated by studies on proteome allocation in Escherichia coli (Basan et al., 2015; Mori et al., 2021; You et al., 2013).“

Line 272, is there a biological reason that oleate may be an outlier? It appears from the figure that metabolites associated with B-oxidation (Pyruate, acetate and oleate) may be creating a different relationship between PT and PC1. For example, is TOR less responsive to variation in TCA/B-oxidation carbon/energy than it is to the sugar related carbon/energy from the other metabolties in Figure 2E. As such, the exclusion of oleate maybe helping to drive the impression of a singular relationship between PT and PC1. It would helpful to be more cautious in the interpretation of the capacity of this singular model to explain the variation in this cross.

We thank Reviewer #1 for the insightful comment on the link between oleate and β-oxidation. Indeed, we cannot rule out that variations in fatty acid metabolism might affect TOR in a manner that differs from what we observed across the other carbon sources

included in the dataset. Our interpretation is, as we mention in the paragraph explaining the PT score, that the PT network state is closely related to the diauxic shift from glucose to ethanol, which is a primary regulatory function of the PKA/TOR pathways. However, this does not preclude the involvement of the same pathways in other regulatory functions and hence, deviation from the "axis" defined by the poles of glucose and ethanol as carbon source. Nevertheless, the difference between the proteome of the oleate sample and the expected proteome given its PT score is not very large. To illustrate this, we now compare the configuration of the oleate sample (expressed as the difference of its protein levels from a virtual centroid defined by the mean of all samples in this dataset) to the effect of chemical inhibition of PKA/TOR as reported in (Kunkel, 2019). This comparison shows that the protein levels in this sample are still fairly well predicted by a general inhibition of PKA/TOR activities (adj. $R^2 = 0.28$, $p < 1E-15$, Figure EV2D) in comparison to the dataset centroid. Following the suggestion of Reviewer #1, we highlighted proteins that are annotated for fatty acid oxidation. These are strongly increased in comparison to the centroid. This is also the case for the glycerol sample, whereas there is no increase of fatty acid oxidation-annotated proteins during growth on acetate and pyruvate above the level expected based on the PT score for these samples (data not shown).
It would be of interest to test whether growth supported by different activities of fatty acid oxidation shifts cells on an axis that is (to some degree) orthogonal to the PT network state. To understand the resulting interplay of different network state-regulatory mechanisms would indeed further increase our understanding of this 'global' type of cellular regulation. We now calculate the linear model between the PT score and PC1 for the dataset from Paulo, 2016 without exclusion of the oleate sample. Further, we describe the deviation of this sample in some detail and mention the limitation of our analysis to a specific network state mechanism:

"The predictive accuracy of the PT score for PC1 scores in a linear model across conditions reached up to 93 % (adj. R^2, $p< 1E-5$, Figure 2D) and PC1 scores in this dataset reflected more than 60 % of total proteome variability. A noticeable exception was the sample grown on oleate, which deviated most from the observed correlation between PC1 and the PT score. Comparison of the proteomic state in this sample to the results of chemical inhibition of PKA and TOR still showed good agreement (linear model adj. $R^2 = 0.28$, $p < 1E-25$, Figure EV2D). However, proteins annotated for fatty acid oxidation (GO: 0019395) were more strongly increased than expected. Together with the deviation of this sample from the global trend, this suggests that growth on lipids could result in an alternative cellular configuration, which is not entirely captured in the PT score. Taken together, these comparisons indicate that the PT score captures a mode of cellular re-configuration that is related to the diauxic shift and to the changes occurring between a broad range of carbon sources."

> *Line 586-597, is the S. pombe comparison showing the broader utility of this approach or is it showing that Yeast have a similar niche and thereby similar architecture on TOR. Or is it showing that domestication of yeasts follows similar avenues? The application to another species is very interesting but I'm not sure that the use of S. pombe allows for the breadth of the introduction and the discussion. It is possible that the architecture of the idea is more generally applicable than the focus on TOR. TOR may have something to do with Yeast ecology that does not apply across other species. For example, in line 649, one could argue that yeasts are not a large evolutionary distance.*

We fully agree with the reviewer, “that the architecture of the idea is more generally applicable than the focus on TOR”. PKA and TOR signaling serve a very central and coordinating function in yeasts, which may very well be related to the specific ecological niches colonized by these species. Budding and fission yeast represent a large evolutionary distance since the *Schizosaccharomyces* clade was estimated to have split off from the *Saccharomyces cerevisiae* branch 250 mio. years ago (Rhind, 2011). What we found particularly striking in this regard is that aerobic fermentation or the Crabtree effect have apparently evolved in these clades by means of convergent evolution (Rhind, 2011). This suggests that the regulatory program associated with the switch from fermentative to respiratory energy metabolism also evolved independently. The ability to fine-tune central carbon metabolism and correspondingly adjust the cellular molecular network certainly represents an evolutionary advantage for both species. We do believe that the concept of network state-regulatory mechanisms is more broadly applicable. Metabolic adjustments of primary carbon metabolism by the ERECTA gene in *Arabidopsis thaliana* is an interesting example in this respect. However, including yet other regulatory mechanisms and covering metazoan species would be beyond scope for this manuscript. We have rephrased the corresponding statements to acknowledge the limitation of our study to yeast species:

Introduction: “*Finally, we show that the PT network state is conserved in fission yeast, which evolved aerobic alcohol fermentation in parallel to budding yeast over an estimated evolutionary distance of more than 200 million years (Rhind et al., 2011).”*
Results, section 7: “*We conclude that the PT network state explains long-range network effects and fitness consequences of genetic variants across a range of growth conditions and in distantly related species of yeast.”*

Reviewer #2:

In this paper, Weith et al. address an important question in complex trait genetics: which genetic perturbations lead to widespread changes in the cellular network versus those that are confined to a small subset of cellular functions? To do so, they introduce the concept of a single "PT" score meant to capture the activity of the PKA and TOR pathways and that is calculated as a weighted average of protein levels (or, later on, transcript levels) of key genes related to these pathways.
To arrive at the score, the authors begin by QTL-mapping the growth response of a yeast segregant panel to a sublethal heat stress and relate variation in heat recovery to proteome data the authors collected earlier. Using a series of analyses in numerous other yeast datasets, the authors show that the PT score captures much of the variation in proteomes in a variety of contexts, suggesting that the score is a useful measure of a critical axis (fermentation vs. respiration) along which cell state varies. The authors go on to describe how genetic variants impact the PT score by either modulating one or few genes, a single module, or variation across the entire cell network. The paper concludes by showing that the PT score is also a useful concept in yeast species other than cerevisiae.

We thank Reviewer #2 for their appreciation of our work and the precise summary.

Overall, I enjoyed this paper. It presents a comprehensive and detailed exploration of the authors' ideas and lays out a useful framework for thinking about complex traits. The text is easy to follow, and the figures are clear and attractive. The paper is a little unusual in how it begins with a relatively small QTL mapping experiment, spends most of its text on computational reanalyses of existing data, and then crams a fairly large amount of experimental work (a three-cross, two-condition eQTL mapping experiment in fission yeast) into a single section (out of 9 sections). However, this unusual structure ends up working well in weaving together the different elements of the paper.

We thank Reviewer #2 for this comment on the structure of the paper. Together with the following comment and a comment by Reviewer #3, we have taken this as an incentive to streamline our description of the PT network state. We have reduced the number of sections from 9 to 7 and have overall shortened the text. We hope that the experimental work in fission yeast is better visible in the new structure.

I only have a few minor comments:

1. The paper is quite long, and I suggest the authors find ways to shorten the main text. It probably isn't necessary to describe each of the many results in as much detail as currently presented. For example, there are a lot of results on the PT score, where exactly it maps, which proteomes it correlates with and how, etc. This could probably be streamlined - after all, it is not surprising that a score derived from protein abundances will, for example, map where the protein abundances map (Figure 2F / section 4), and correlate with protein abundances in other datasets. Some detail in this and other sections can probably be moved to Supplement.

We appreciate these suggestions by Reviewer #2 and have shortened the description of results pertaining directly to the PT score. We have moved the results for the QTL mapping of the PT score to section 4. Here, these results serve to illustrate how loci with and without high numbers of pQTL targets are involved in shaping the PT network state.

2. Line 272: the callout to Figure 2E does not seem to be correct, or rather, the panel labels in Figure 2 may not be correct. In the figure, "E" seems to label the phosphoproteomics results rather than the PC1/PK score panel, which seems to have no dedicated label. Rather than using "2D lower panel", I'd just use more lettered panels.

3. Line 281: Figure 2F does not show PT score correlations with "the first dimension in a PCA of the BYxRM proteomes" - it shows QTL traces. Mislabeled panel?

4. Line 319: callout to 2G should be to 2F?

5. Line 330: there is no panel 2H

6. Did panel 2E (the actual 2E, showing phosphoproteome results) ever get mentioned in the text?

We apologize for the mislabeling of panels in Figure 2 and have now corrected these along with the callouts in the main text. We have also changed the figure structures in general to prefer lettered panels.

> *7. L. 361: it is not clear to me across which data these correlations were computed. A given gene is either assigned to a given GO term or not - where does abundance variation come into play here?*

We thank Reviewer #2 for pointing out this lack of precision in the description of the results illustrated in Figure 3. These correlations pertain to the abundance variation of proteins assigned to the respective GO terms across strains of the BYxRM collection. We have changed the description of these results to be more precise:

"The analyses above were consistent with the coordination of a large diversity of cellular processes by the PKA/TOR-related regulatory program. In order to characterize the association between the PT score and cellular processes and functions, we first assigned proteins in our dataset for the BYxRM collection and corresponding phosphopeptides to the SGD-curated set of Gene Ontology (GO) slim terms (Cherry et al., 2012). We then correlated the individual proteins and peptides in each of these GO terms with the PT score. Each GO term is represented by an individual pie chart that shows the proportion of significantly predicted proteins and is positioned according to the average direction and accuracy of prediction by the PT score across the members of the term (Figure 3)."

> *8. L. 400: I don't understand the use of "adaption" of cellular processes here. Adaptation in what sense? Presumably not evolutionarily? Do the authors just mean "change", or "shifts"? The same comment applies to line 20 in the graphical abstract and line 414. There probably are more. Please change or explain what is meant here.*

We have followed the suggestion by Reviewer #2 and removed the term 'adaptation' in the presentation of network state-related changes. Here, we do not specifically refer to the evolutionary aspects of molecular network state regulation but rather describe the observation of network state differences upon specific perturbations or as a result of genetic variation in specific crosses.

Reviewer #3:

> *In this study, Weith et al. perform a series of physiological and multi-omic analyses in a mapping population of yeast strains (the BYxRM cross). They conclude that a "network state" corresponding to PKA/TOR signaling activity (the PT state) is the dominant axis of variation driving multiple physiological processes, including heat-induced lag and environmental changes in carbon sources. They summarize the PT state using a one-dimensional PT score, which they then genetically map. Much of the results section is devoted to motivating, defining, and characterizing the PT state. Their essential conceptual assertion is that genetic variants in this cross effectively cluster into three groups, which they call local, regional, and global. Global genetic effects are those that have a large influence on the PT score, which encodes complex constraints on the molecular physiology. Regional and local effects, by contrast, have detectable effects as protein abundance quantitative trait loci (pQTLs), but have only small effects on the PT*

score. In support of this claim, they perform a bioinformatic network analysis quantifying the network distances of the targets of pQTL hotspots. They show that pQTL hotspots that colocalize with QTLs for PT state (i.e. global effects) have more distant target genes than hotspots that do not correspond to PT state QTLs (i.e., local and regional effects). They interpret this result to mean that global effects that integrate up to cellular physiology must necessarily target a set of functionally distant genes.

Overall, I found the presentation challenging and had to dig through too many details to understand their essential point. In particular, there is an inconsistency in the writing between advocating for a novel conceptual model for genetic effects (local, regional, and global) vs. arguing for the importance of PT network state per se. The genetic analysis of low-dimensional representations of multi-omic data is not new, and their data show that PT state strongly correlates with principal components (PCs) of several omics signatures. Thus, the paper could be substantially streamlined by removing the detailed description and characterization of the PT state, which, for all intents and purposes, functions as a marker for the first PC across different conditions.

We thank Reviewer #3 for the precise summary and the comments on the overall structure of the manuscript. The description of the PT network state is intended to illustrate one mechanism by which genetic effects can propagate in a 'global' manner across the molecular network. In response to the criticism regarding a too detailed description of this particular regulatory mechanism, we have shortened the corresponding sections (Results, Section 2 and Section 3, see changes in the revised manuscript).
In principle, the low-dimensional representation of omics data is indeed not a new concept. However, our analysis goes beyond this data reduction step by developing a mechanistic explanation for the observed variation in the data. Specifically, we observe that the first PC strongly correlates with the PT score across many conditions and that a significant proportion of genetic variation between yeast strains can be attributed to PT network state differences. However, this is not universally true: for example, the PT score does not capture the first principal component of variation in the cross between the fission yeast strains L968 and Y0036 in the absence of oxidative stress (Figure EV5B). A new analysis of loci, which affect the PT network state without displaying high numbers of pQTL targets ('PT4' - 'PT8', new Section 4 in Results) indicate that such 'global' mechanisms can be moderate in size and hence, do not necessarily cause major variability.
It is important to clarify that our description of the PT network state is not intended to exhaustively describe all variation in the yeast crosses that we analyzed. Importantly, we do not exclude and indeed assume the existence of alternative, in part orthogonal network state-regulatory pathways. We now mention an observation related to fatty acid oxidation when comparing the PT score across the first principal component of samples grown on a range of carbon sources (see our response to Reviewer #1 above). Furthermore, we now state more clearly in Results, Section 4 that our analysis of global vs. local or regional effects is not limited to the PT network state in particular:

"[…] differences in the PT score were associated with effects in distant parts of the molecular network – which we term 'global network effects'. We speculate that this global network reorganization results from the functional requirement to adjust multiple cellular functions (or network modules) to each other. Importantly, we do not claim that such global network effects are limited to alterations of the PT signaling state. Instead, alterations of other central

signaling pathways may also exert effects on a broad range of coordinated cellular functions."

If, on the other hand, the main assertion is instead that the PT state is an essential single dimension defining many responses of yeast to perturbations, then this should be stated clearly and does not appear to need a new conceptual model for the genetic architecture of complex traits.

The main conceptual novelty in our work is to describe a model for network effects of genetic variants. We present the PT network state in some detail as a mechanistic example to describe and infer how global effects arise. To clarify these points, we performed a refined analysis of the network spread of genetic variants in order to generalize this concept and make it transferable to other organisms, in which other network state-regulatory mechanisms may be observable (new Results section 4).

I found the network analysis (Fig. 4) conceptually elegant, but I have concerns with their approach. Could they simply plot the average network distance between pQTL targets and the effect size of the locus on PT score? This seems the most straightforward way to establish the validity of their model.

We thank Reviewer #3 for this suggestion, which has resulted in a new panel (Figure 4D), where we are now plotting the average network distance among QTL targets as a function of the QTL effect size (number of targets). Additional analyses are shown below. This new analysis has resulted in a number of interesting findings, including the observation that targets of most QTL tend to be closer in the network than expected by chance and highlighting the distinct widespread network effects of the *IRA2* locus. Furthermore, we have identified other loci with more direct effects on the PT network state (loci 'PT4' - 'PT8' and the *IRA2* hotspot, see Results, new section 4) that also affected proteins that are more distant in the network than the global average, thereby corroborating the notion that large changes of the PT score associate with distant (global) network effects. However, the analysis of all pQTL loci is complicated by limited resolution of individual variants in our dataset. For loci with few pQTL targets, this results in an increased level of noise (high variability of average network distance among targets and variability of observed PT score differences due to LD with variants with strong effects). Loci with stronger effects can be analyzed in this fashion. However, for strong perturbations, the likelihood that other central signaling pathways are affected in addition to PKA/TOR signaling, increases. Hence, global effects can arise in a different manner.
Clearly, the PT network state is only an example for the global reorganization of molecular networks to genetic (and other) perturbations. However, in the budding- and fission yeast crosses that we have analyzed, it seems to explain major parts of the phenotypic variation. The mechanistic conclusions are further strengthened by a new analysis presented in Figure 4E: here we have compared the network distances between correlated proteins (proteins with correlated expression changes across the RMxBY collection) to their association with the PT score. In a nutshell, our analysis shows that proteins being subject to regional effects are often positively correlated to each other, while protein pairs being affected by long-range (global) effects are increasingly anti-correlated (negatively correlated). This is consistent with our notion that global effects arise from the adaptation of distant network modules to each other, such as glycolysis versus oxidative phosphorylation (Figure 3): if one goes up the

other one goes down. This yields anti-correlation for protein pairs across these two modules, whereas protein pairs within the modules will be positively correlated to maintain stoichiometry of the process.

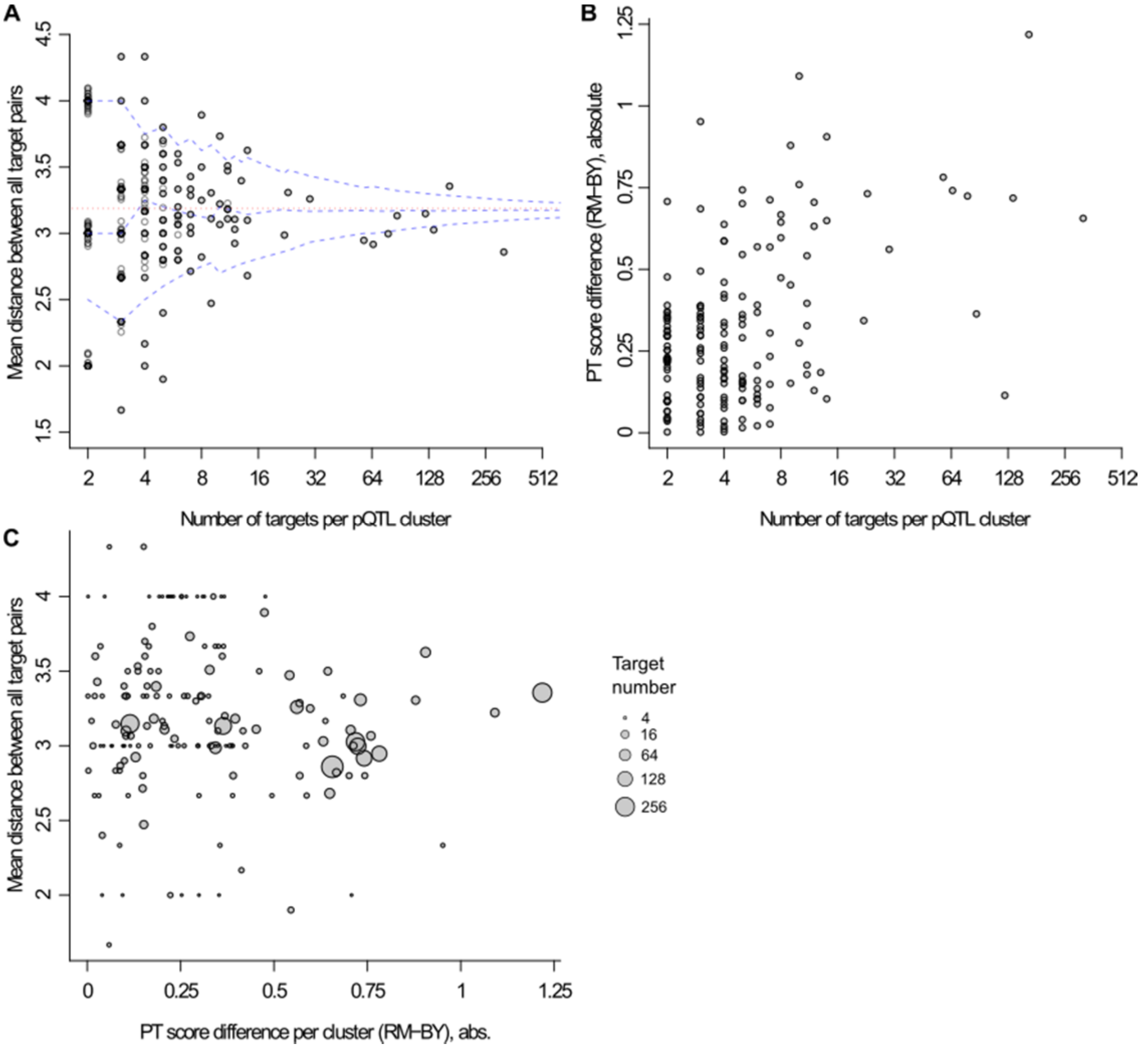

Figure: Comparison between number of targets, PT score difference and average target network distance.

A Comparison between number of targets and average target network distance for 265 pQTL clusters as in Figure 4D (revised manuscript). **B** Comparison between number of targets and PT score difference. **C** Comparison between PT score difference and average target network distance. Target number (log2) is illustrated by the diameter of the respective data point.

More generally, I am concerned that there is a potential circularity in their data that is not discussed. It is a tautology that if a locus has a large influence on PT score, then it will necessarily have many pQTLs. Equivalently, if a locus does not have many pQTLs, it cannot have a large influence on PT score. They show that the network spread of the hotspot targets does not correlate with the number of targets, but the important question is whether, after controlling for the number of targets, the spread of a hotspot significantly

predicts the effect of the locus on PT score. This would show specifically that it is the targeting of functionally distant genes that is critical for altering the PT "network state".

Since Reviewer #1 raised similar concerns about the analysis of network state differences, we have thoroughly reviewed the analysis of network distance among targets of different types of genetic variants (Results, Section 4). As we now show by the example of five loci (‘PT4’ - ‘PT8’), PT network effects also originate at loci with no or only few significantly affected targets. We agree that large effects on the PT network state will lead to many pQTL mapping to the respective locus. However, we observed a clear difference in the network spread of targets of loci with primarily regional effects, such as most pQTL hotspots, to the network spread of the IRA2 locus and PT4 through PT8. We present the results of our modified analysis in the revised manuscript and further describe our observations in the response to the comment by Reviewer #1 above.

Finally, I would like to see a discussion of how they propose we use their proposed model for genetic effects. Is it predictive, in that we can know a priori local, from regional, from global effects just from, say, proteomic data? (A significant correlation between network spread and effect size for PT state would argue yes.) Should this model alter how we design experiments or analyses for complex traits? If so, how? Without such discussion, we are left wondering what the authors make of these observations.

We very much thank the reviewer for this question as it is critical to define the relevance of our work. To address these important points we have expanded the discussion in the manuscript in the following way:

“Our work suggests a rational for the conditions under which genetic effects have long-range consequences in molecular networks: whenever distant network modules require a specific balance to maintain cellular homeostasis, long-range effects can ensue. This concept will aid future genetic mapping and association studies because it provides a mechanistic explanation for why certain genetic effects are pleiotropic. Notably, our concept differentiates between pleiotropism in terms of effect size and pleiotropism in terms of the spread of genetic effects across the network. […]

Our model helps explaining the joined effect of multiple genetic variants and quantification of the PT network state even enabled the prediction of outcomes of genetic configurations without pinpointing individual variants.

[…] the PT network state serves as an example of a – genetically controlled – network state associated with a global re-organization of the cell. We detected several loci with comparably weak effects (showing a low number of significantly affected protein targets) but detectable differences in the PT network state. The most affected proteins at these loci displayed larger network distances than the targets of most hotspots. Hence, weak effects can be pleiotropic in the sense that they result in changes in distant parts of the molecular network. Effect size and network spread are therefore not strictly coupled. We propose that far-reaching genetic effects, such as those postulated in the concept of ‘omnigenic inheritance’ (Boyle et al., 2017) may originate from adjustments in cellular physiology.

Our work provides a schema for causally linking genetic variation to general physiological programs: it begins with the identification and quantification of network states that serve as low-dimensional, biologically interpretable representations of complex cellular states, proceeds by characterizing their cellular function, analyzing genetic and environmental mechanisms affecting them, and ultimately synthesizing that information to explain differences between individuals. By developing molecular signatures that faithfully capture a particular network state, we will be able to interpret transcriptomic data (such as those collected by the GTEx consortium) in quantitative physiological terms and to explain the convergence of hitherto seemingly unrelated genetic variation on more general regulatory programs. Exemplarily, we confirmed conservation of the PT network state between Saccharomyces cerevisiae and Schizzosaccharomyces pombe despite millions of years and different paths of evolution that separate these species (Rhind et al., 2011). In mammalian cells, the transition between respiratory and glycolytic metabolism is known as the 'Warburg effect' (Liberti and Locasale, 2016), which was first observed in carcinogenesis but has since been described in many other circumstances such as cellular differentiation (Bhattacharya et al., 2020; Zhang et al., 2018) and ageing-associated disease (Traxler et al., 2022). For example, we have recently detected an association between ERK signaling (the mammalian equivalent of the PKA pathway) and the co-occurrence of aging-associated diseases (Fraser et al., 2022). Here, we have shown the utility of quantifying network states for explaining the consequences of genomic variability for cellular traits. We propose to test other regulatory mechanisms that exert developmental or metabolic control for network state-regulating properties and to chart their natural genetic variation. This framework will help to better understand (i) the polygenicity of complex traits, (ii) the propagation of genetic effects in molecular networks and thereby (iii) pleiotropic effects."

References:

Boyle, E.A., Li, Y.I., and Pritchard, J.K. (2017). An Expanded View of Complex Traits: From Polygenic to Omnigenic. Cell *169*, 1177-1186. 10.1016/j.cell.2017.05.038.

Kerwin, R.E., Jimenez-Gomez, J.M., Fulop, D., Harmer, S.L., Maloof, J.N., and Kliebenstein, D.J. (2011). Network Quantitative Trait Loci Mapping of Circadian Clock Outputs Identifies Metabolic Pathway-to-Clock Linkages in Arabidopsis. The Plant Cell *23*, 471-485. 10.1105/tpc.110.082065.

Keurentjes, J.J.B., Sulpice, R., Gibon, Y., Steinhauser, M.-C., Fu, J., Koornneef, M., Stitt, M., and Vreugdenhil, D. (2008). Integrative analyses of genetic variation in enzyme activities of primary carbohydrate metabolism reveal distinct modes of regulation in Arabidopsis thaliana. Genome Biology *9*. 10.1186/gb-2008-9-8-r129.

Kunkel, J., Luo, X., and Capaldi, A.P. (2019). Integrated TORC1 and PKA signaling control the temporal activation of glucose-induced gene expression in yeast. Nat Commun *10*, 3558. 10.1038/s41467-019-11540-y

Liu, X., Li, Y.I., and Pritchard, J.K. (2019). Trans Effects on Gene Expression Can Drive Omnigenic Inheritance. Cell *177*, 1022-1034 e1026. 10.1016/j.cell.2019.04.014.

Rhind, N., Chen, Z., Yassour, M., Thompson, D.A., Haas, B.J., Habib, N., Wapinski, I., Roy, S., Lin, M.F., Heiman, D.I., et al. (2011). Comparative functional genomics of the fission yeasts. Science *332*, 930-936. 10.1126/science.1203357.

You, C., Okano, H., Hui, S., Zhang, Z., Kim, M., Gunderson, C.W., Wang, Y.P., Lenz, P., Yan, D., and Hwa, T. (2013). Coordination of bacterial proteome with metabolism by cyclic AMP signalling. Nature *500*, 301-306. 10.1038/nature12446.

30th May 2023

Manuscript Number: MSB-2022-11493R
Title: Genetic effects on molecular network states explain complex traits

Dear Andreas,

Thank you for sending us your revised manuscript. We have now heard back from the three reviewers who were asked to evaluate your revised study. As you will see below, the reviewers are satisfied with the performed revisions and support publication. They do raise however some remaining minor concerns, which we would ask you to address by editing the text. We would also ask you to address some remaining editorial issues listed below.

- Our data editors have noted some missing information in the figure legends, please see the attached .doc file. Please make all requested text changes using the attached file and *keeping the "track changes" mode* so that we can easily access the edits made.

- Please include 5 keywords.

- There is a callout for Fig. 3C, but no such panel exists, please check.

- All fields of the author checklist need to be completed, please upload an updated checklist file.

- Four EV Figures have been uploaded but the numbers are EV1, EV2, EV4 and EV6. Please check and either provide the missing Figures EV3 and EV5 or renumber the four EV figures to: EV1, EV2, EV3 and EV4. The callouts in the text need to be corrected accordingly.

- Table EV1 should be renamed to Dataset EV1 with the appropriate callout, and the legend should be provided in a separate sheet in the Excel file.

- We typically do not encourage Supplementary Text. If it is impossible to incorporate it in the main text (either in the Results or in the Methods), we would ask you to provide it in an Appendix PDF (a single file) as Appendix Text 1 and Appendix Text 2.

Please resubmit your revised manuscript online, with a covering letter listing amendments and responses to each point raised by the referees. Please resubmit the paper **within one month** and ideally as soon as possible. If we do not receive the revised manuscript within this time period, the file might be closed and any subsequent resubmission would be treated as a new manuscript. Please use the Manuscript Number (above) in all correspondence.

When you resubmit your manuscript, please download our CHECKLIST (https://bit.ly/EMBOPressAuthorChecklist) and include the completed form in your submission. *Please note* that the Author Checklist will be published alongside the paper as part of the transparent process (https://www.embopress.org/page/journal/17444292/authorguide#transparentprocess)

Click on the link below to submit your revised paper.

https://msb.msubmit.net/cgi-bin/main.plex

As a matter of course, please make sure that you have correctly followed the instructions for authors as given on the submission website.

Thank you for submitting this paper to Molecular Systems Biology.

Kind regards,

Maria

Maria Polychronidou, PhD
Senior Editor
Molecular Systems Biology

If you do choose to resubmit, please click on the link below to submit the revision online before 29th Jun 2023.

https://msb.msubmit.net/cgi-bin/main.plex

IMPORTANT:
Please note that corresponding authors are required to supply an ORCID ID for their name upon submission of a revised manuscript (EMBO Press signed a joint statement to encourage ORCID adoption).
(https://www.embopress.org/page/journal/17444292/authorguide#editorialprocess)
Currently, our records indicate that the ORCID for your account is 0000-0002-3891-2123.

Please click the link below to modify this ORCID:
Link Not Available

The system will prompt you to fill in your funding and payment information. This will allow Wiley to send you a quote for the article processing charge (APC) in case of acceptance. This quote takes into account any reduction or fee waivers that you may be eligible for. Authors do not need to pay any fees before their manuscript is accepted and transferred to the publisher.

*** PLEASE NOTE *** As part of the EMBO Press transparent editorial process initiative (see our Editorial at https://dx.doi.org/10.1038/msb.2010.72 , Molecular Systems Biology will publish online a Review Process File to accompany accepted manuscripts. When preparing your letter of response, please be aware that in the event of acceptance, your cover letter/point-by-point document will be included as part of this File, which will be available to the scientific community. More information about this initiative is available in our Instructions to Authors. If you have any questions about this initiative, please contact the editorial office (msb@embo.org).

Reviewer #1:

The authors have worked to reply to the variety of requests. It still is a very heavy read which may be a component of the topic. Similarly, a focus on human papers as a way to provide application potential actually overlooks the vast potential applications in synthetic biology, fungal engineering and plant and animal breeding where if anything quantitative network biology is of more immediate use than human biology. Although, the human opacity in these introductions is a field standard. Given the responses and adjustments, I have no further significant suggestions.

Reviewer #2:

Great job on the revision. I only have a few remaining comments:

Line 601: "rational" should be "rationale"

Line 606: "pleiotropism" should be "pleiotropy"

Finally, I was unable to follow the description of the results in Figure 4E (lines 430 to 445). Because I don't understand it I'm also not sure how to fix it, but maybe the authors can take another swing at making this section more accessible. Maybe it can also just be deleted.

Reviewer #3:

I credit the authors for the substantial work on the manuscript, which has significantly improved its rigor and readability. In particular, in the current version "loci with large effects on PT state" is not as easily conflated (by the reader) with "loci with global effects", as their example of PTQTL2 and vs. PTQTLs 4-8 now make clear. Thus, their classification presents an orthogonal concept to the idea of large-effect QTLs driving system-level expression programs. I have no major concerns, but I believe the following points could do with further refinement:

1) In their introduction (line 102) they describe global effects intuitively as "variants affecting the balance between seemingly unrelated network modules". It is clear from what follows that this really means that, while molecular networks are organized into modules, some variants influence the expression of proteins in modules that are distant from each other in the network. At the risk of being pedantic, I think the phrasing "seemingly unrelated" is too subjective here and reminds me of similar language that was used decades ago to operationally define "pleiotropy", which has caused no end of confusion. I think it makes sense here to use a more objective phrase, such as "molecularly distant" or similar.

2) Their new observation that protein pairs with high network distance have a higher frequency of anti-correlation at the protein

level is intriguing and strongly argues for their view that "global effects" relate to fundamental physiological constraints; when some things go up, others must come down. However, it seems natural that there is no clear dividing line separating "few" vs. "many" anti-correlated pairs for a particular pQTL hotspot; in other words, no hard and fast distinction between regional vs. global effects. It is more likely a spectrum, that could potentially even be quantified with a network statistic (e.g., are the sign patterns modular? frustrated? etc.). While such an extended characterization is clearly beyond the scope of this work, it seems worth discussing the possibility of characterizing such patterns at the level of pQTL hotspots a priori, before (or in parallel) with physiological patterns.

3) Finally, the use of network distance on its own is appealing and clearly suited to their purpose, the existence of small-effect-size global variants seems to call out for a network-based explanation. For example, are the causal variants in genes with high betweeness for the target proteins? Can we characterize why such effects are global despite being small effect? A further analysis is beyond the scope of of this work, but a typical reader of Molecular Systems Biology will be primed to imagine some kind of network-based statistic that may be predictive of regional- vs. global-effect variants and a discussion of this may be warranted.

Reviewer #1:

The authors have worked to reply to the variety of requests. It still is a very heavy read which may be a component of the topic. Similarly, a focus on human papers as a way to provide application potential actually overlooks the vast potential applications in synthetic biology, fungal engineering and plant and animal breeding where if anything quantitative network biology is of more immediate use than human biology. Although, the human opacity in these introductions is a field standard. Given the responses and adjustments, I have no further significant suggestions.

We thank reviewer #1 for the positive overall evaluation of the revised manuscript. Due to space limitations and since it is beyond our expertise, we do not discuss applications in the fields mentioned by reviewer #1 but we highly appreciate this comment.

Reviewer #2:

Great job on the revision. I only have a few remaining comments:

Line 601: "rational" should be "rationale"

Line 606: "pleiotropism" should be "pleiotropy"

We thank reviewer #2 for these observations and have adjusted the text accordingly.

Finally, I was unable to follow the description of the results in Figure 4E (lines 430 to 445). Because I don't understand it I'm also not sure how to fix it, but maybe the authors can take another swing at making this section more accessible. Maybe it can also just be deleted.

We have rephrased the description of the results in Figure 4E as suggested by reviewer #2. We believe that these results are important to further elucidate the mechanism of global effect variants and therefore refrained from deleting this section. We hope that the rephrased version as shown below is more accessible:

“The global character of PTQTL effects suggested that two proteins that are under the common control of the PT network state can be correlated even if they are only distantly related in the network. To test this, we computed pair-wise correlations among all proteins and binned all pairs by network distance. Next, we quantified to what extent a correlated pair of proteins (|r| >0.5) was under the control of (or associated with) the PT network state. To quantify this, the correlation of the individual proteins with the PT score was calculated. For anti-correlated pairs, one of the correlation coefficients was sign-inverted. Finally, the absolute value of the mean of both individual correlations served as a measure of ‘compatibility’ with PT network state alteration as a driver of the correlation for each protein pair. This analysis revealed that correlated protein pairs falling into low distance bins (bins 1-3) were less correlated with the PT score (|mean (sign-corrected) r of individual (paired) proteins with PT score| > 0.5 for 46 % of 48722 pairs) than protein pairs in higher distance bins (71 % of 10892 pairs, p < 1e-15, Fisher’s exact test). Furthermore, anti-correlated protein pairs were more often associated with PT score differences than correlated protein pairs and anti-correlation was more prominent in higher network distance bins than in lower network distance bins (Figure 4E). Hence, the proportions of correlation and anti-correlation among targets of a QTL might be helpful to characterize genetic effects in terms of their spread across the network.”

Reviewer #3:

I credit the authors for the substantial work on the manuscript, which has significantly improved its rigor and readability. In particular, in the current version "loci with large effects

on PT state" is not as easily conflated (by the reader) with "loci with global effects", as their example of PTQTL2 and vs. PTQTLs 4-8 now make clear. Thus, their classification presents an orthogonal concept to the idea of large-effect QTLs driving system-level expression programs. I have no major concerns, but I believe the following points could do with further refinement:

1) In their introduction (line 102) they describe global effects intuitively as "variants affecting the balance between seemingly unrelated network modules". It is clear from what follows that this really means that, while molecular networks are organized into modules, some variants influence the expression of proteins in modules that are distant from each other in the network. At the risk of being pedantic, I think the phrasing "seemingly unrelated" is too subjective here and reminds me of similar language that was used decades ago to operationally define "pleiotropy", which has caused no end of confusion. I think it makes sense here to use a more objective phrase, such as "molecularly distant" or similar.

We thank reviewer #3 for the positive comment on the revised manuscript. The phrasing used to describe global effects in this section of the introduction was indeed imprecise. The text now reads as follows:

"[…] (3) variants affecting the balance between seemingly unrelated network modules that reside in regions of the network which are less connected to each other by (macro)molecular interactions."

2) Their new observation that protein pairs with high network distance have a higher frequency of anti-correlation at the protein level is intriguing and strongly argues for their view that "global effects" relate to fundamental physiological constraints; when some things go up, others must come down. However, it seems natural that there is no clear dividing line separating "few" vs. "many" anti-correlated pairs for a particular pQTL hotspot; in other words, no hard and fast distinction between regional vs. global effects. It is more likely a spectrum, that could potentially even be quantified with a network statistic (e.g., are the sign patterns modular? frustrated? etc.). While such an extended characterization is clearly beyond the scope of this work, it seems worth discussing the possibility of characterizing such patterns at the level of pQTL hotspots a priori, before (or in parallel) with physiological patterns.

The suggestion by reviewer #3 to establish further quantitative criteria to distinguish regional from global effects, for example based on the patterns of correlation vs. anti-correlation is intriguing. While we feel that we have reached the limitations of our dataset with the presented analyses, we now highlight this potential for future research. We further share the expectation of reviewer #3 that there is likely no strict boundary separating local, regional and global effects and mention this in the discussion:

Results section:

"Furthermore, anti-correlated protein pairs were more often associated with PT score differences than correlated protein pairs and anti-correlation was more prominent in higher network distance bins than in lower network distance bins (Figure 4E). Hence, the proportions of correlation and anti-correlation among targets of a QTL might be helpful to characterize genetic effects in terms of their spread across the network."

Discussion:

"Among proteins whose abundance correlated with the PT network state, anti-correlation was more frequent than among proteins which correlated independently of the PT network

state. Network-based statistics such as ‘betweenness’ could further serve as quantifiable criteria to determine the type of genetic effects in an a priori manner when individual variants and hence, variant genes can be identified. Unfortunately, the genetic resolution of our data was insufficient to confidently assign sufficient numbers of QTL to individual variants or genes to allow further analysis in this direction. It is also important to note that there may be no objective, quantifiable boundaries separating local from regional and regional from global effects. Instead, we expect a continuum of network changes and the terms 'local', 'regional' and 'global' should help to conceptualize the different nature of QTL effects on molecular networks. For example, we propose that far-reaching genetic effects, such as those postulated in the concept of ‘omnigenic inheritance’ (Boyle et al., 2017) may originate from adjustments in cellular physiology, caused by genetic variants with global effect components.”

3) Finally, the use of network distance on its own is appealing and clearly suited to their purpose, the existence of small-effect-size global variants seems to call out for a network-based explanation. For example, are the causal variants in genes with high betweeness for the target proteins? Can we characterize why such effects are global despite being small effect? A further analysis is beyond the scope of of this work, but a typical reader of Molecular Systems Biology will be primed to imagine some kind of network-based statistic that may be predictive of regional- vs. global-effect variants and a discussion of this may be warranted.

We appreciate the suggestion of reviewer #3 to use network statistics for the immediate target genes as another quantifiable measure to enhance the distinction between the different proposed types of genetic variants. Due to the limited size of our yeast collection, we only have limited confidence in the assignment of QTL to individual genes, especially for variants with weak effects. Therefore, we refrained from attempts that would require this direct assignment when no further evidence was present. Hence, we were unable to determine such network statistics for the weak PTQTL that we describe (PT4-PT8). However, we now highlight the potential to apply such analyses as a complementary approach to the network distance measure in the discussion section cited above.

4th Jul 2023

Manuscript number: MSB-2022-11493RR
Title: Genetic effects on molecular network states explain complex traits

Dear Andreas,

Thank you again for sending us your revised manuscript. We are now satisfied with the modifications made and I am pleased to inform you that your paper has been accepted for publication.

*** PLEASE NOTE *** As part of the EMBO Publications transparent editorial process initiative (see our Editorial at https://dx.doi.org/10.1038/msb.2010.72), Molecular Systems Biology publishes online a Review Process File with each accepted manuscripts. This file will be published in conjunction with your paper and will include the anonymous referee reports, your point- by-point response and all pertinent correspondence relating to the manuscript. If you do NOT want this File to be published, please inform the editorial office at msb@embo.org within 14 days upon receipt of the present letter.

Should you be planning a Press Release on your article, please get in contact with msb@wiley.com as early as possible, in order to coordinate publication and release dates.

LICENSE AND PAYMENT:
All articles published in Molecular Systems Biology are fully open access: immediately and freely available to read, download and share.

Molecular Systems Biology charges an article processing charge (APC) to cover the publication costs. You, as the corresponding author for this manuscript, should have already received a quote with the article processing fee separately. Please let us know in case this quote has not been received.

Once your article is at Wiley for editorial production you will receive an email from Wiley's Author Services system, which will ask you to log in and will present you with the publication license form for completion. Within the same system the publication fee can be paid by credit card, an invoice or pro forma can be requested.

Payment of the publication charge and the signed Open Access Agreement form must be received before the article can be published online.

Molecular Systems Biology articles are published under the Creative Commons licence CC BY, which facilitates the sharing of scientific information by reducing legal barriers, while mandating attribution of the source in accordance to standard scholarly practice.

Proofs will be forwarded to you within the next 2-3 weeks.

Thank you very much for submitting your work to Molecular Systems Biology.

Kind regards,

Maria

Maria Polychronidou, PhD
Senior Editor
Molecular Systems Biology

EMBO Press Author Checklist

Corresponding Author Name: Dr. Andreas Beyer	
Journal Submitted to: Molecular Systems Biology	
Manuscript Number: MSB-2022-11493	

USEFUL LINKS FOR COMPLETING THIS FORM
The EMBO Journal - Author Guidelines
EMBO Reports - Author Guidelines
Molecular Systems Biology - Author Guidelines
EMBO Molecular Medicine - Author Guidelines

Reporting Checklist for Life Science Articles (updated January
This checklist is adapted from Materials Design Analysis Reporting (MDAR) Checklist for Authors. MDAR establishes a minimum set of requirements in transparent reporting in the life sciences (see Statement of Task: 10.31222/osf.io/9sm4x). Please follow the journal's guidelines in preparing your
Please note that a copy of this checklist will be published alongside your article.

Abridged guidelines for figures
1. Data
The data shown in figures should satisfy the following conditions:
- the data were obtained and processed according to the field's best practice and are presented to reflect the results of the experiments in an accurate and unbiased manner.
- ideally, figure panels should include only measurements that are directly comparable to each other and obtained with the same assay.
- plots include clearly labeled error bars for independent experiments and sample sizes. Unless justified, error bars should not be shown for technical
- if n<5, the individual data points from each experiment should be plotted. Any statistical test employed should be justified.
- Source Data should be included to report the data underlying figures according to the guidelines set out in the authorship guidelines on Data

2. Captions
Each figure caption should contain the following information, for each panel where they are relevant:
- a specification of the experimental system investigated (eg cell line, species name).
- the assay(s) and method(s) used to carry out the reported observations and measurements.
- an explicit mention of the biological and chemical entity(ies) that are being measured.
- an explicit mention of the biological and chemical entity(ies) that are altered/varied/perturbed in a controlled manner.
- the exact sample size (n) for each experimental group/condition, given as a number, not a range;
- a description of the sample collection allowing the reader to understand whether the samples represent technical or biological replicates (including how many animals, litters, cultures, etc.).
- a statement of how many times the experiment shown was independently replicated in the laboratory.
- definitions of statistical methods and measures:
  - common tests, such as t-test (please specify whether paired vs. unpaired), simple χ2 tests, Wilcoxon and Mann-Whitney tests, can be unambiguously identified by name only, but more complex techniques should be described in the methods section;
  - are tests one-sided or two-sided?
  - are there adjustments for multiple comparisons?
  - exact statistical test results, e.g., P values = x but not P values < x;
  - definition of 'center values' as median or average;
  - definition of error bars as s.d. or s.e.m.

Please complete ALL of the questions below.
Select "Not Applicable" only when the requested information is not relevant for your study.

Materials

Newly Created Materials	Information included in the manuscript?	In which section is the information available? (Reagents and Tools Table, Materials and Methods, Figures, Data Availability Section)
New materials and reagents need to be available; do any restrictions apply?	Not Applicable	

Antibodies	Information included in the manuscript?	In which section is the information available? (Reagents and Tools Table, Materials and Methods, Figures, Data Availability Section)
For **antibodies** provide the following information:<br>- Commercial antibodies: RRID (if possible) or supplier name, catalogue number and or/clone number<br>- Non-commercial: RRID or citation	Not Applicable	

DNA and RNA sequences	Information included in the manuscript?	In which section is the information available? (Reagents and Tools Table, Materials and Methods, Figures, Data Availability Section)
**Short novel DNA or RNA including primers, probes**: provide the sequences.	Yes	Materials and Methods

Cell materials	Information included in the manuscript?	In which section is the information available? (Reagents and Tools Table, Materials and Methods, Figures, Data Availability Section)
**Cell lines:** Provide species information, strain. Provide accession number in repository **OR** supplier name, catalog number, clone number, and/**OR** RRID.	Not Applicable	
**Primary cultures:** Provide species, strain, sex of origin, genetic modification status.	Not Applicable	
Report if the cell lines were recently **authenticated** (e.g., by STR profiling) and tested for mycoplasma contamination.	Not Applicable	

Experimental animals	Information included in the manuscript?	In which section is the information available? (Reagents and Tools Table, Materials and Methods, Figures, Data Availability Section)
**Laboratory animals or Model organisms:** Provide species, strain, sex, age, genetic modification status. Provide accession number in repository **OR** supplier name, catalog number, clone number, **OR** RRID.	Not Applicable	
**Animal observed in or captured from the field:** Provide species, sex, and age where possible.	Not Applicable	
Please detail **housing and husbandry conditions**.	Not Applicable	

Plants and microbes	Information included in the manuscript?	In which section is the information available? (Reagents and Tools Table, Materials and Methods, Figures, Data Availability Section)
**Plants:** provide species and strain, ecotype and cultivar where relevant, unique accession number if available, and source (including location for collected wild specimens).	Not Applicable	
**Microbes:** provide species and strain, unique accession number if available, and source.	Yes	Materials and Methods

Human research participants	Information included in the manuscript?	In which section is the information available? (Reagents and Tools Table, Materials and Methods, Figures, Data Availability Section)
If collected and within the bounds of privacy constraints report on age, sex and gender or ethnicity for all study participants.	Not Applicable	

Core facilities	Information included in the manuscript?	In which section is the information available? (Reagents and Tools Table, Materials and Methods, Figures, Data Availability Section)
If your work benefited from core facilities, was their service mentioned in the acknowledgments section?	Not Applicable	

Design

Study protocol	Information included in the manuscript?	In which section is the information available? (Reagents and Tools Table, Materials and Methods, Figures, Data Availability Section)
If study protocol has been **pre-registered, provide DOI in the manuscript**. For clinical trials, provide the trial registration number **OR** cite DOI.	Not Applicable	
Report the **clinical trial registration number** (at ClinicalTrials.gov or equivalent), where applicable.	Not Applicable	

Laboratory protocol	Information included in the manuscript?	In which section is the information available? (Reagents and Tools Table, Materials and Methods, Figures, Data Availability Section)
Provide DOI OR other citation details if **external detailed step-by-step protocols** are available.	Not Applicable	

Experimental study design and statistics	Information included in the manuscript?	In which section is the information available? (Reagents and Tools Table, Materials and Methods, Figures, Data Availability Section)
Include a statement about **sample size** estimate even if no statistical methods were used.	Yes	Figures and main text
Were any steps taken to minimize the effects of subjective bias when allocating animals/samples to treatment (e.g. **randomization procedure**)? If yes, have they been described?	Not Applicable	
Include a statement about **blinding** even if no blinding was done.	Not Applicable	
Describe **inclusion/exclusion criteria** if samples or animals were excluded from the analysis. Were the criteria pre-established?<br><br>If sample or data points were omitted from analysis, report if this was due to attrition or intentional exclusion and provide justification.	Not Applicable	
For every figure, are **statistical tests** justified as appropriate? Do the data meet the assumptions of the tests (e.g., normal distribution)? Describe any methods used to assess it. Is there an estimate of variation within each group of data? Is the variance similar between the groups that are being statistically compared?	Yes	Figures and main text

Sample definition and in-laboratory replication	Information included in the manuscript?	In which section is the information available? (Reagents and Tools Table, Materials and Methods, Figures, Data Availability Section)
In the figure legends: state number of times the experiment was **replicated** in laboratory.	Yes	Figures and main text
In the figure legends: define whether data describe **technical or biological replicates.**	Yes	Figures and main text

Ethics

Ethics	Information included in the manuscript?	In which section is the information available? (Reagents and Tools Table, Materials and Methods, Figures, Data Availability Section)
Studies involving **human participants**: State details of **authority granting ethics approval** (IRB or equivalent committee(s), provide reference number for approval.	Not Applicable	
Studies involving **human participants**: Include a statement confirming that **informed consent** was obtained from all subjects and that the experiments conformed to the principles set out in the WMA Declaration of Helsinki and the Department of Health and Human Services Belmont Report.	Not Applicable	
Studies involving **human participants:** For publication of **patient photos**, include a statement confirming that consent to publish was obtained.	Not Applicable	
Studies involving experimental **animals**: State details of **authority granting ethics approval** (IRB or equivalent committee(s), provide reference number for approval. Include a statement of compliance with ethical regulations.	Not Applicable	
Studies involving **specimen and field samples:** State if relevant **permits** obtained, provide details of authority approving study; if none were required, explain why.	Not Applicable	

Dual Use Research of Concern (DURC)	Information included in the manuscript?	In which section is the information available? (Reagents and Tools Table, Materials and Methods, Figures, Data Availability Section)
Could your study fall under dual use research restrictions? Please check biosecurity documents and list of **select agents and toxins** (CDC): https://www.selectagents.gov/sat/list.htm	Not Applicable	
If you used a select agent, is the security level of the lab appropriate and reported in the manuscript?	Not Applicable	
If a study is subject to dual use research of concern regulations, is the name of the **authority granting approval and reference number** for the regulatory approval provided in the manuscript?	Not Applicable	

Reporting

The MDAR framework recommends adoption of discipline-specific guidelines, established and endorsed through community initiatives. Journals have their own policy about requiring specific guidelines and recommendations to complement MDAR.

Adherence to community standards	Information included in the manuscript?	In which section is the information available? (Reagents and Tools Table, Materials and Methods, Figures, Data Availability Section)
State if relevant guidelines or checklists (e.g., **ICMJE, MIBBI, ARRIVE, PRISMA**) have been followed or provided.	Not Applicable	
For **tumor marker prognostic studies,** we recommend that you follow the **REMARK** reporting guidelines (see link list at top right). See author guidelines, under 'Reporting Guidelines'. Please confirm you have followed these guidelines.	Not Applicable	
For **phase II and III randomized controlled trials**, please refer to the **CONSORT** flow diagram (see link list at top right) and submit the CONSORT checklist (see link list at top right) with your submission. See author guidelines, under 'Reporting Guidelines'. Please confirm you have submitted this list.	Not Applicable	

Data Availability

Data availability	Information included in the manuscript?	In which section is the information available? (Reagents and Tools Table, Materials and Methods, Figures, Data Availability Section)
Have **primary datasets** been deposited according to the journal's guidelines (see 'Data Deposition' section) and the respective accession numbers provided in the Data Availability Section?	Yes	Data Availability Section
Were **human clinical and genomic datasets** deposited in a public access-controlled repository in accordance to ethical obligations to the patients and to the applicable consent agreement?	Not Applicable	
Are **computational models** that are central and integral to a study available without restrictions in a machine-readable form? Were the relevant accession numbers or links provided?	Not Applicable	
If publicly available data were reused, provide the respective **data citations in the reference list**.	Yes	Materials and Methdos, Figures, main text, References
